# A multi-day and multi-band dataset for a steady-state visual-evoked potential–based brain-computer interface

**DOI:** 10.1093/gigascience/giz133

**Published:** 2019-11-25

**Authors:** Ga-Young Choi, Chang-Hee Han, Young-Jin Jung, Han-Jeong Hwang

**Affiliations:** 1 Department of Medical IT Convergence Engineering, Kumoh National Institute of Technology, Daehak-ro 61, Gumi 39177, Republic of Korea; 2 Machine Learning Group, Berlin Institute of Technology (TU Berlin), Marchstrasse 23, Berlin 10587, Germany; 3 Department of Radiological Science, Dongseo University, Jurye-ro 47, Busan 47011, Republic of Korea

**Keywords:** steady-state visual-evoked potential (SSVEP), brain-computer interface (BCI), electroencephalography (EEG), physiological data

## Abstract

**Background:**

A steady-state visual-evoked potential (SSVEP) is a brain response to visual stimuli modulated at certain frequencies; it has been widely used in electroencephalography (EEG)-based brain–computer interface research. However, there are few published SSVEP datasets for brain–computer interface. In this study, we obtained a new SSVEP dataset based on measurements from 30 participants, performed on 2 days; our dataset complements existing SSVEP datasets: (i) multi-band SSVEP datasets are provided, and all 3 possible frequency bands (low, middle, and high) were used for SSVEP stimulation; (ii) multi-day datasets are included; and (iii) the EEG datasets include simultaneously obtained physiological measurements, such as respiration, electrocardiography, electromyography, and head motion (accelerator).

**Findings:**

To validate our dataset, we estimated the spectral powers and classification performance for the EEG (SSVEP) datasets and created an example plot to visualize the physiological time-series data. Strong SSVEP responses were observed at stimulation frequencies, and the mean classification performance of the middle frequency band was significantly higher than the low- and high-frequency bands. Other physiological data also showed reasonable results.

**Conclusions:**

Our multi-band, multi-day SSVEP datasets can be used to optimize stimulation frequencies because they enable simultaneous investigation of the characteristics of the SSVEPs evoked in each of the 3 frequency bands, and solve session-to-session (day-to-day) transfer problems by enabling investigation of the non-stationarity of SSVEPs measured on different days. Additionally, auxiliary physiological data can be used to explore the relationship between SSVEP characteristics and physiological conditions, providing useful information for optimizing experimental paradigms to achieve high performance.

## Data Description

### Background and purpose

A brain–computer interface (BCI) is a non-muscular communication method that uses brain activity, such as the electroencephalogram (EEG), to assist individuals with disabilities who are unable to voluntarily control their bodies [1, 2]. Two approaches have been used to develop EEG-based BCIs; the difference between these 2 approaches is the presence of external stimuli [3]. Endogenous BCIs use mental imagery tasks to induce certain brain patterns, whereas exogenous BCIs use external stimuli to evoke certain brain patterns.

A representative endogenous BCI paradigm is motor imagery, which is defined as the mental simulation of motor behaviors, e.g., left/right hand movement [4, 5]. Owing to the event-related (de)synchronization phenomenon, different motor imagery tasks can be discriminated by using machine learning techniques; the discrimination results can then be used for BCI applications [6, 7]. To date, a large number of motor imagery BCI datasets have been published [8–14], and they have significantly contributed to the advancement of BCI research. Other endogenous types of BCI datasets are also available, such as slow cortical potential, readiness potential [8], and mental arithmetic datasets [13, 14].

There are 2 representative exogenous BCI paradigms: event-related potentials (ERPs) and steady-state visual-evoked potentials (SSVEPs). An ERP is a time-locked brain response that is evoked in response to specific visual, auditory, and/or tactile stimuli, whereas an SSVEP is a period brain response to a visual stimulus modulated at a certain frequency. ERPs have mostly been used in the development of row/column matrix spellers [15], whereas SSVEPs have been used in the development of a variety of BCI applications, such as robotic arm control [16], exoskeletons [17], functional electrical stimulation [18], and word spellers [19, 20]. Many ERP BCI datasets have become publicly available since the first ERP BCI dataset was published in 2003 [8]. However, it was not until 2017 that a freely accessible SSVEP BCI dataset was published for the first time [21]; it was followed by the second dataset in 2019, although the SSVEP paradigm has been widely used in BCI applications because high performance can be achieved with minimal training [22].

Because the number of SSVEP BCI datasets is small compared with the number of datasets based on other BCI paradigms, it would be beneficial for BCI researchers to provide a new SSVEP BCI dataset that can complement the existing SSVEP BCI datasets. The first SSVEP dataset was created from the data of 35 participants who used a 40-target BCI speller; the SSVEP stimulation frequencies ranged from 8 to 15.8 Hz, with a span of 0.2 Hz [21]. The second SSVEP dataset was acquired based on the data from 54 participants who used a 4-class BCI system over 2 sessions; 5.45, 6.67, 8.57, and 12 Hz were used as stimulation frequencies [22].

In this study, we created a new SSVEP BCI dataset that can contribute to SSVEP-based BCI research in 3 ways. First, our SSVEP dataset consists of 3 sub-datasets, each with a different frequency band: low (1–12 Hz), middle (12–30 Hz), and high (30–60 Hz). It is well documented that SSVEPs are elicited over a wide range of frequencies, from 1 to 90 Hz [23], and that the frequencies can be divided into 3 sub-frequency bands (i.e., low, middle, and high), as mentioned above [24]. The 2 previous SSVEP datasets were acquired by applying stimulation frequencies in certain frequency bands, i.e., 8–15.8 Hz in the low- and middle-frequency bands [21] and 5.45–12 Hz in the low-frequency band [22]. Considering that the choice of the stimulation frequency band is an important factor that significantly affects SSVEP-based BCI performance [25], the characteristics of the SSVEPs evoked in each of the 3 frequency bands should be investigated in coincidence with the corresponding signal-to-noise ratio (SNR) and classification performance. In particular, the high-frequency band is currently receiving increasing attention as an alternative to the low- and middle-frequency bands, despite it being associated with relatively low performance, because it results in less visual fatigue [26]. However, no SSVEP BCI studies have provided available datasets for the high-frequency band. Thus, it is necessary to have access to an SSVEP dataset that includes high-frequency band data, in addition to low- and middle-frequency band data, to investigate the aforementioned peculiarities. Our SSVEP dataset satisfies this requirement because it includes data for each of the 3 frequency bands that were independently acquired from the same participants. Second, we provide a multi-session (multi-day) dataset that was recorded over 2 different days from the same participants. Thus, our SSVEP dataset can be used to study session-to-session transfer, which is a challenging problem in BCI research [27–29]. A multi-session SSVEP dataset was also provided in [22]; however, it was acquired on the same day, with only a short break (i.e., 3 min). Therefore, our dataset can offer more profound insight into the non-stationary nature of EEG signals and thereby provide useful solutions to session-to-session (day-to-day) transfer problems. Last, we provide other physiological data for the dataset, i.e., data that have not been included in the 2 previously published SSVEP datasets [22, 23], in addition to the EEG dataset, to evaluate changes to the physiological condition of participants during the experiment, such as respiration, electrocardiography (ECG), neck electromyography (EMG), and head motion. The auxiliary physiological data can be used to explore the relationships between SSVEP characteristics (e.g., SNR) and various physiological variables, thereby providing information that can be used to design experimental paradigms to achieve high performance.

To create a novel SSVEP BCI dataset that is complementary to the 2 currently available SSVEP BCI datasets, we designed a 4-class SSVEP paradigm that is similar to that used to acquire the second SSVEP BCI dataset [22]. Three sets of 4 stimulation frequencies were used for the low-, middle-, and high-frequency bands, respectively. The SSVEP BCI dataset was created using data that were collected from 30 participants on 2 different days. For data validation, we applied a standard analysis method to our SSVEP dataset and analyzed the baseline results in consideration of all of the aforementioned physiological data that were obtained in this study.

### Experimental design

#### Participants

A total of 30 participants (9 women and 21 men; mean [SD] age, 23.8 [1.3] years) were recruited for this study. The number of participants was set at 30 because a sample size of 30 is sufficient to apply parametric statistical tests for analysis of the results. Note that parametric statistical tests provide more statistical power than non-parametric ones and thereby ensure more reliable validation of our SSVEP dataset. No participant had any history of psychiatric disease that could have affected the research results. Seven of the 30 participants had prior BCI experience, but they participated in endogenous BCI experiments that required them to perform a mental arithmetic task. Thus, it was assumed that their prior BCI experience would not significantly affect the research results. Before the experiment, they were given the details of the experimental procedures and signed a form providing informed consent for study participation and the anonymous release of their data to the public. Adequate reimbursement was provided for their participation after the experiment. This study was approved by the Institutional Review Board of Kumoh National Institute of Technology (No. 6250) and was conducted in accordance with the principles of the declaration of Helsinki.

#### Stimulator

**Figure 1:**
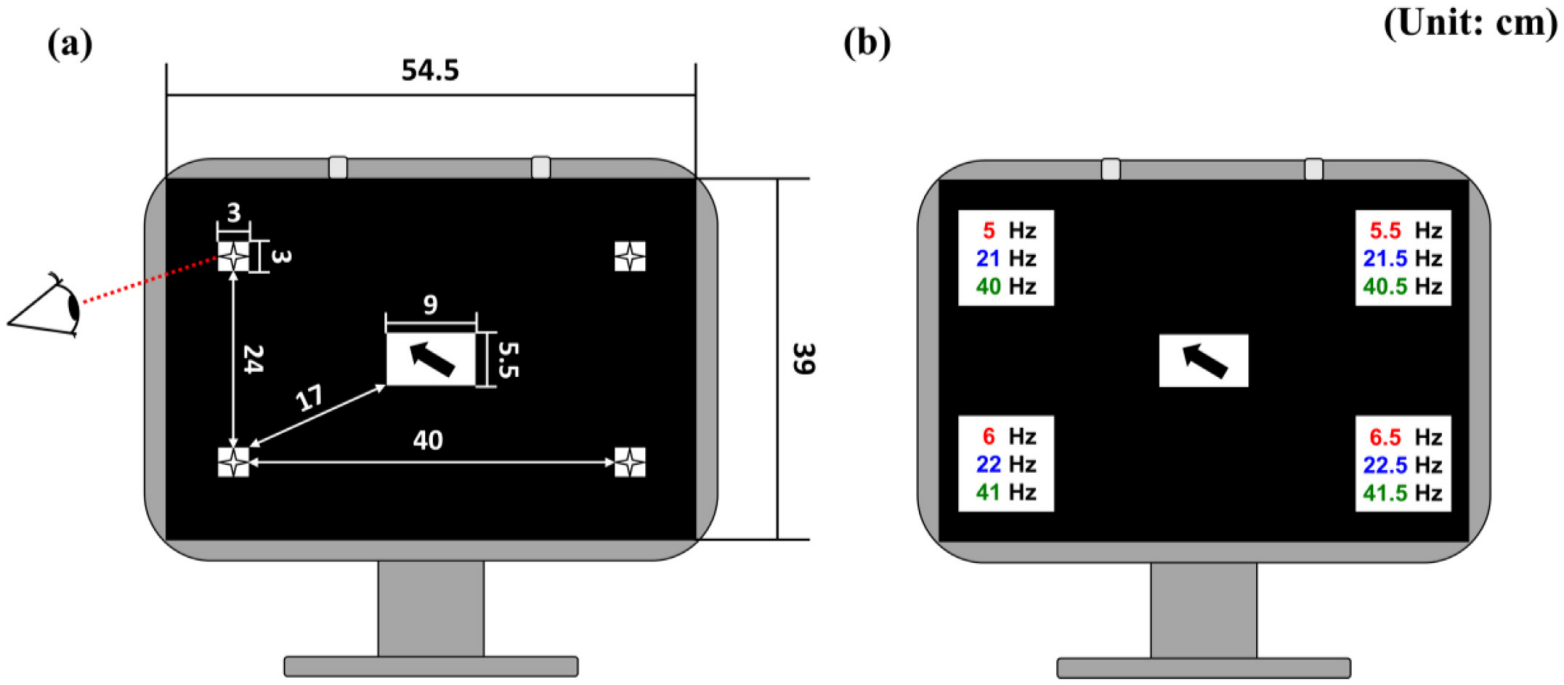
(a) Schematic diagram of the SSVEP stimulator (unit: cm). (b) Placement of the 4 stimulation frequencies for each of the 3 stimulation frequency bands (5.0, 5.5, 6.0, and 6.5 Hz for the low-frequency band; 21.0, 21.5, 22.0, and 22.5 Hz for the middle-frequency band; 40.0, 40.5, 41.0, and 41.5 Hz for the high-frequency band).

The SSVEP stimulator was made of 2 square pieces of styrofoam, a sheet of thick black paper, an opaque film, 4 LEDs, and an LED controller. We first cut 1 of the styrofoam pieces to make 5 sections, 4 of which were 3 cm × 3 cm and purposed for the LED display; the other was 9 cm × 5.5 cm and purposed to show instructions during the experiment (Fig. 1a). After that, we inserted 4 LEDs into the 4 square holes that were punctured through the 3 cm × 3 cm sections (part No.: T03WC01; operating current: 20 mA; viewing angle: θ/2 = 100°; luminous intensity: 2,000 mcd; emitting color: white; Yinhui Photoelectric Technology Co. Ltd, Shandong, China) and attached another styrofoam piece to the back of the sectioned styrofoam. The front part of the stimulator was covered with an opaque film to diffuse the light, and then we attached a piece of black paper with 5 square holes, which were exactly matched to those punctured through the front styrofoam piece, to the opaque film for better visibility. The stimulator was attached to a 21-inch LCD monitor (Trigem Computer Inc., Seoul, Republic of Korea) and an instruction, i.e., on which LED the participant should focus, was presented by means of an arrow from the monitor through the center square hole of the 9 cm × 5.5 cm styrofoam piece. A schematic diagram of the SSVEP stimulator is shown in Fig.   1a. The distance between each LED and instruction arrow presented at the center of the monitor was 17 cm. To control the stimulator, we used a LAUNCHXL-F28027 Board powered by C2000 MCU (Texas Instruments, Dallas, TX 75243, US) . The duty cycle was set at 50%, meaning that the LED had 50% on-time and 50% off-time.

As mentioned above, 3 different frequency bands (low: 1–12 Hz, middle: 12–30 Hz, and high: 30–60 Hz [30]) were individually applied for SSVEP stimulation to obtain multi-band SSVEP datasets in this study. Three sets of 4 stimulation frequencies were implemented for each frequency band, as follows: 5.0, 5.5, 6.0, and 6.5 Hz for the low-frequency band; 21.0, 21.5, 22.0, and 22.5 Hz for the middle-frequency band; and 40.0, 40.5, 41.0, and 41.5 Hz for the high-frequency band. The 4 stimulation frequencies for each frequency band were selected such that the harmonic frequencies of the 4 frequencies in the low-frequency band would not overlap with any of the 4 frequencies in the middle- or high-frequency bands, and the harmonic frequencies of the 4 frequencies in the middle-frequency band would not overlap with the 4 frequencies in the high-frequency band. This was done because simultaneous implementation of the harmonic frequencies as different stimulation frequencies can significantly decrease the performance of SSVEP-based BCI systems [31]. Additionally, the α frequency band was not considered because its use can produce a considerable number of false-positive results [30, 32], even though using the α band for SSVEP stimulation tends to yield a high SNR. We assigned 4 stimulation frequencies to 4 LEDs, depending on the stimulation frequency band, as shown in Fig. 1b.

#### Experimental paradigm

During the experiment, each participant sat in a comfortable armchair that was placed 1 m from the SSVEP stimulator, which was attached to a 21-inch monitor, and was instructed to remain relaxed without any movement. Note that all instructions were presented at the center of the monitor, and the participants could view them through the center hole of the stimulator. For each trial, a blank screen was presented for 5 s, and then an arrow indicating 1 of the 4 LEDs was presented for 6 s; during this time, the participant was asked to gaze at the target LED, as instructed by the direction of the arrow. Subsequently, a white plus sign was presented for 6 s to indicate a short break before the next trial. A short beep sound was also presented with every visual stimulus transition to explicitly capture the attention of the participants. The direction of each arrow was randomly presented 20 times (20 trials) for each direction, resulting in a total of 80 trials; this was repeated for each frequency band (i.e., low, middle, and high). To prevent excessive fatigue, a minimum 5-min break was allotted to each participant after every 40 trials (40 trials equate to 1 session); irregular breaks were also allowed as requested by the participants during the experiment. Each participant performed 6 sessions of the SSVEP experiment (i.e., 2 sessions × 3 frequency bands) twice on different days, with an interval of ≥1 day. The order of the stimulation frequency band trials was varied for counterbalancing between participants. In particular, all possible order combinations of the 3 frequency bands were as follows: low-middle-high, low-high-middle, middle-low-high, middle-high-low, high-low-middle, and high-middle-low. Each order combination was randomly assigned to 5 participants (6 combinations × 5 participants = 30 participants), and the same order was used on both days once it was assigned to the participant on the first day of the experiment. The entire experiment lasted ∼2 h each day, including the time for EEG preparation.

#### Data recording

**Figure 2:**
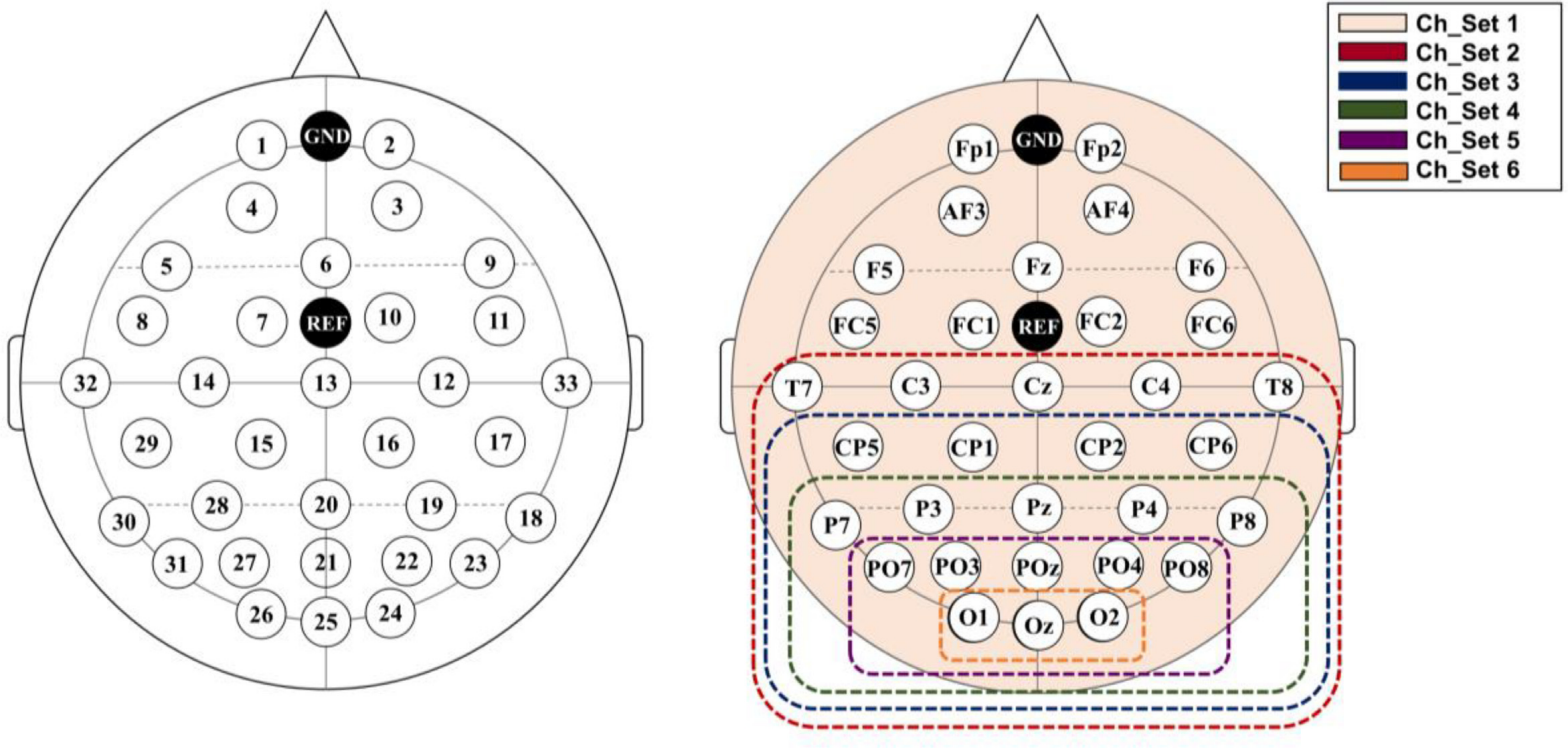
Electrode positions used in the experiment with respect to the (a) number and (b) position name. Note that 6 different channel sets were used for data analysis to evaluate the impact of the number of electrodes on classification performance.

The EEG signals were measured by using a BrainAmp EEG amplifier (Brain products, GmbH Ltd., Gilching, Germany) with a sampling rate of 1,000 Hz; the ground and reference electrodes were respectively attached at Fpz and FCz sites (Fig. 2). We used 33 active electrodes, which were mounted according to the International 10-10 system, to measure EEG signals (FP1, FP2, AF4, AF3, F5, Fz, FC1, FC5, F6, FC2, FC6, C4, Cz, C3, CP1, CP2, CP6, P8, P4, Pz, POz, PO4, PO8, O2, Oz, O1, PO3, P3, CP5, P7, PO7, T7, and T8); electrodes were more densely mounted around occipital areas, relative to other areas, because the SSVEPs mainly originated from the occipital lobe. We did not control for changes to electrode locations between the 2 days; we instead tried to maintain the conditions of EEG measurement between the 2 days for each participant. This is because slight changes to electrode locations are inevitable, as it would happen with daily BCI use; thus, our dataset can effectively address session-to-session (day-to-day) transfer problems. Note that electrode location change between days is an important factor in EEG non-stationarity between days [33].

We also measured various physiological signals as the EEG signals were measured, i.e., respiration, ECG, neck EMG, and head motion, to investigate physiological changes. To measure these physiological signals, we attached a respiratory belt to the chest, 3 ECG sensors on lead-I position (Einthoven's triangle), 2 EMG sensors on the right and left sides of the neck, and an inertial measurement unit (IMU) sensor on the top of the head between Cz and CPz. The same amplifier that was used for measuring EEG signals was used to record the physiological signals at the same sampling rate of 1,000 Hz; thus, all of the measured data were synchronized. The physiological data can be used to investigate the relationships between changes in brain activity and various physiological variables, as well as to develop artifact correction algorithms. For example, some researchers previously simultaneously used EEG and ECG to evaluate the psychological state and stress level/mental effort of participants [34, 35], whereas others used motion data to remove motion-related artifacts from EEG data [36, 37].

#### Data format and structure

Because data analysis was performed using Matlab R2013b (MathWorks, Natick, MA, USA), we provide our dataset in the form of Matlab files (.mat). Each data folder has 2 subfolders, each containing a subdataset corresponding to 1 of the 2 experimental days (i.e., Day 1 and Day 2). Each subfolder has cnt and mrk files, which contain continuous time-series data for all physiological measurements (cnt) and the corresponding data with the trigger information (mrk), respectively. The cnt and mrk files have suffixes corresponding to 3 frequency bands and session numbers. For example, cnt_Low(1) denotes time-series data that were obtained by using the low-frequency band for SSVEP stimulation in the first session. Thus, the subfolder for each participant contains the following 6 pairs of cnt and mrk files: cnt_Low(1), mrk_Low(1), cnt_Low(2), mrk_Low(2), cnt_Middle(1), mrk_Middle(1), cnt_Middle(2), mrk_Middle(2), cnt_High(1), mrk_High(1), cnt_High(2), and mrk_High (2). All data were down-sampled to 200 Hz when the raw data were converted to Matlab-compatible files. Table 1 lists all of the data files provided for each subfolder.

**Table 1:** Data format

Frequency band	Stimulation frequency (Hz)	Data format (*.mat)
Low	5.0	cnt_Low(1), cnt_Low(2) mrk_Low(1), mrk_Low(2)
	5.5	
	6.0	
	6.5	
Middle	21.0	cnt_Middle(1), cnt_Middle(2) mrk_Middle(1), mrk_Middle(2)
	21.5	
	22.0	
	22.5	
High	40.0	cnt_High(1), cnt_High(2) mrk_High(1), mrk_High(2)
	40.5	
	41.0	
	41.5	

Each data folder for each participant has 2 subfolders that contain 2 subdatasets that correspond to measurements taken on 2 different days, and each subfolder has 6 pairs of cnt and mrk files (shown below) that have been labeled according to the stimulation frequency band and session number.

#### Questionnaire

We asked participants to fill out 2 different questionnaires before and after the experiment. Table 2 presents 2 sets of questionnaires. Seven (A1– A7) and 3 (B1–B3) questions were asked before the experiment to record the demographics and initial physical condition of the participant, and after the experiment to check the physical condition of the participant (i.e., the level of drowsiness, concentration, and eye strain), respectively. The answers to the questionnaires have been provided in a supplementary file (questionnaires_answers.xlsx). Note that, because all participants were university students in their twenties who did not take any medication or drink alcohol 24 h before the experiment, we did not include the related information (i.e., A2: Age Group, A5: Drinking Alcohol, and A7: Medicine) in the supplementary file.

**Table 2:** Two sets of questionnaires answered before and after the experiment

No.	Questionnaire	Answer
Before Experiment		
A1	Sex	Male = 1; Female = 2
A2	Age group	10s = 1; 20s = 2; 30s = 3; ≥40s = 4
A3	Job	Middle/high school student = 1; Undergraduate = 2; Postgraduate = 3; Others = 4
A4	Sleeping hours	Less than 5 h = 1; 6 h = 2; 7 h = 3; 8 h = 4; ≥9 h = 5
A5	Alcohol consumption	No = 1; Yes = 2
A6	Overall body condition	(Good) 1 2 3 4 5 6 7 8 9 10 (Bad)
A7	Medication use	No = 1; Yes = 2
After Experiment		
B1	Drowsiness	(Good) 1 2 3 4 5 6 7 8 9 10 (Bad)
B2	Concentration	(Bad) 1 2 3 4 5 6 7 8 9 10 (Good)
B3	Eye strain	(Good) 1 2 3 4 5 6 7 8 9 10 (Bad)

## Data Validation

### Methods

Because our main concern was the EEG dataset measured during the SSVEP experiment, we provide detailed results of analysis for the EEG dataset, and example time-series data for the other physiological datasets.

The EEG data were first band-pass–filtered with different cutoff frequencies according to the stimulation frequency band, as follows: 3–9, 18–24, and 38–44 Hz for the low-, middle-, and high-frequency bands, respectively. From the band-pass–filtered data, we extracted 6-s epochs that were measured while the participants were focusing on each of the target LEDs, and used them for further analysis. To visualize the SSVEP responses, spectral powers were estimated for each channel by applying a moving-window technique (2.5-s window size with 90% overlap). The SSVEP SNR was also calculated by dividing the SSVEP amplitude at the stimulation frequency by the mean spectral amplitude of 6 adjacent frequencies to demonstrate the reliability of our SSVEP dataset [38].

(1) \begin{equation*} {\rm{SNR}} = \frac{{n \times y\,(f)}}{{\sum\nolimits_{k = 1}^{n/2} {[y(f + 0.5 \times k) + y(f - 0.5 \times k)]} }}, \end{equation*}

where *n* is the number of adjacent points (6 in this study), *y* is the spectral amplitude, and *f* is the stimulation frequency. Canonical correlation analysis, which is the most widely used method for classifying SSVEP data, was used for 4-class classification [39].

Each of the 5 types of physiological data was linearly detrended to remove baseline drift. The respiratory rate and heart rate were respectively estimated using the respiration and ECG data based on the peak information for each frequency band and each session to evaluate the ranges of the respiratory and heart rates. The mean and standard deviation values were estimated for each trial for the other types of physiological signals (i.e., EMG1, EMG2, and IMU) to evaluate changes in each set of physiological data.

### Results

**Figure 3:**
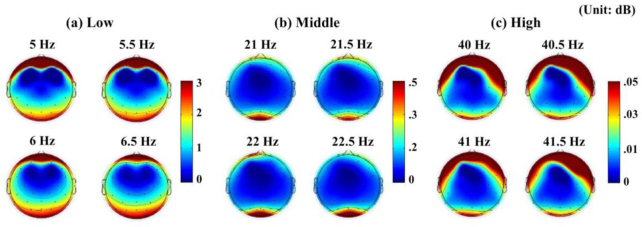
Topographic maps at the SSVEP frequencies averaged over 2 days with all participants for the 4 stimulation frequencies of (a) the low-, (b) middle-, and (c) high-frequency band.

Fig. 3 shows topographic maps corresponding to the SSVEP frequencies, as averaged using the data collected over 2 days for all participants and the 4 stimulation frequencies in each frequency band. As expected, strong SSVEPs were observed near occipital areas in all cases. High spectral powers were also observed near frontotemporal areas, which would be derived from electro-oculography. As is well known, absolute spectral powers decrease from the low-frequency band to the high-frequency band (see the color bar range in Fig. 3). The occipital SSVEPs were high relative to those observed in the other brain areas when the middle-frequency band was applied; a spatially high SSVEP SNR was observed.

**Figure 4:**
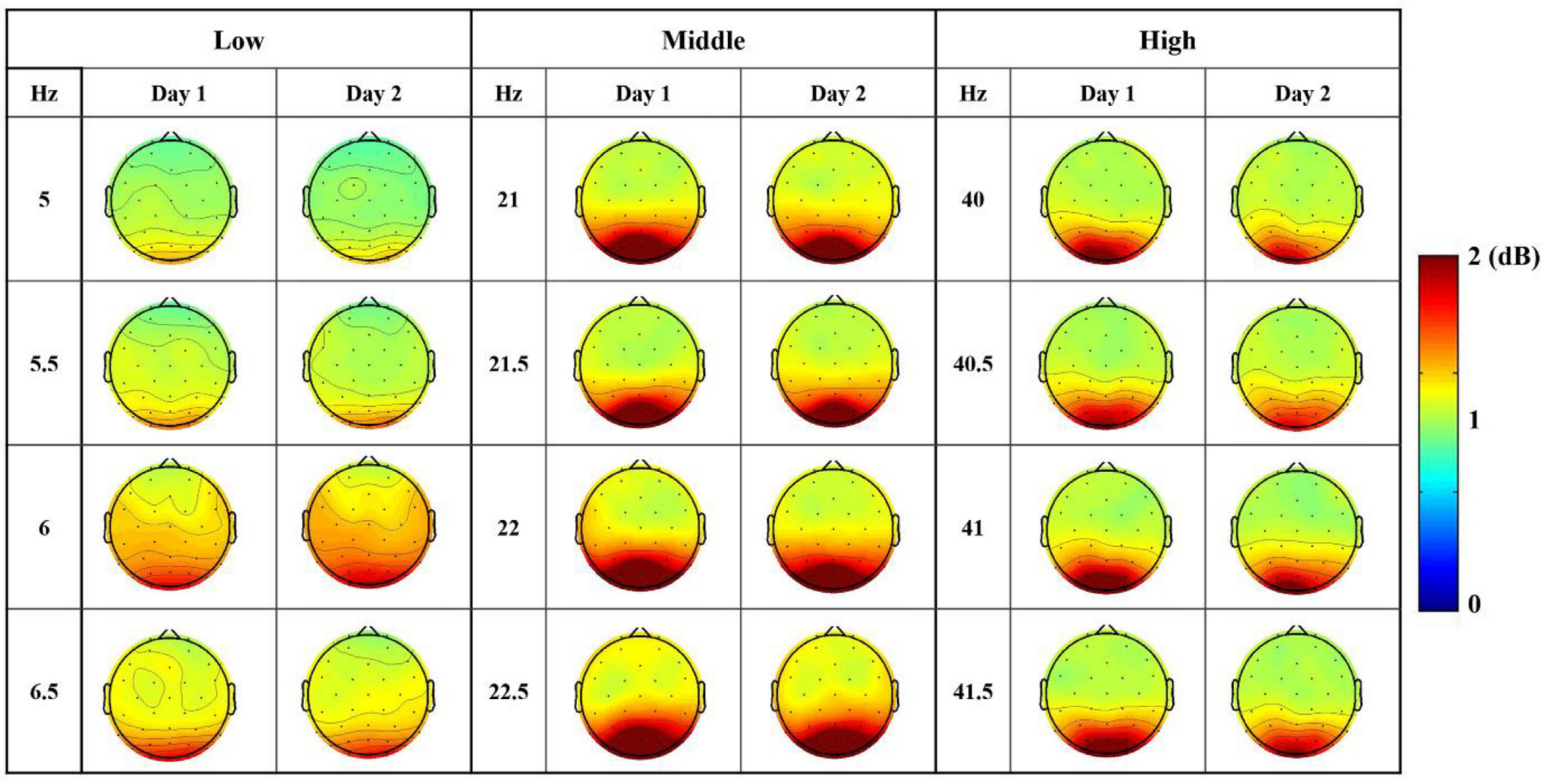
SSVEP SNR topographic maps averaged for each day with all participants for the 4 stimulation frequencies of the low-, middle-, and high-frequency band, respectively. The cross-correlation coefficients are >0.99 for all cases.

Fig. 4 shows SSVEP SNR topographic maps that were averaged using the single-day data for the 4 stimulation frequencies of each frequency band for all participants. Most channels achieved SSVEP SNRs that were >1 for all stimulation frequencies, with parieto-occipital channels achieving high SSVEP SNRs that exceed 2, demonstrating the reliability of our SSVEP datasets. Additionally, the Day 1 and Day 2 SSVEP topographic maps appear to be very similar, corresponding to a high cross-correlation (*r* > 0.99) for all comparison cases. This result demonstrates a small discrepancy between the electrode locations on the first and second days. All SSVEP SNRs are provided with 12 supplementary files (4 stimulation frequencies × 3 frequency bands) for each day, and each supplementary file contains the SSVEP SNR data for each channel and trial for all participants. The cross-correlation analysis results for each participant are also provided for the 4 stimulation frequencies in each frequency band with a supplementary file (SNR_CrossCorrelation.xlsx).

**Figure 5:**
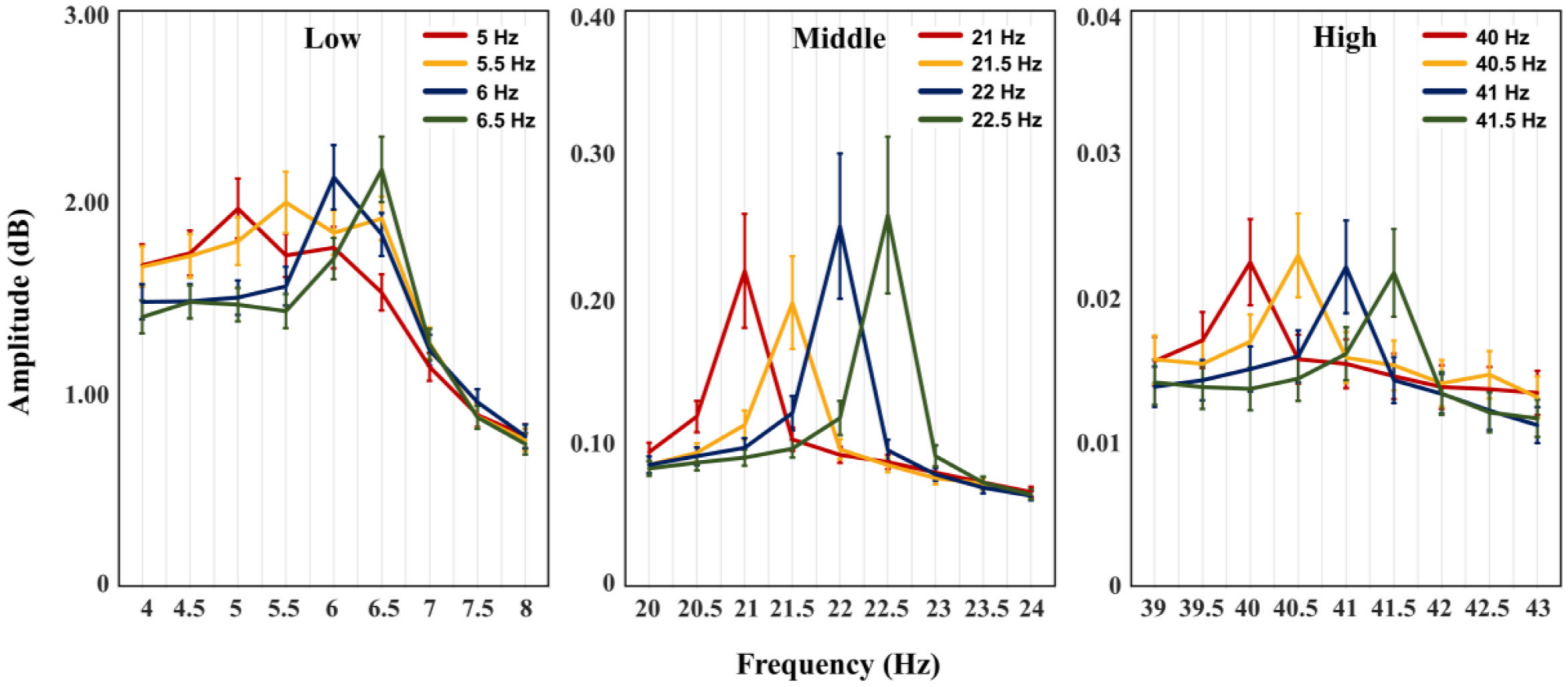
Grand-average SSVEP responses estimated based on the data from 13 parieto-occipital channels (Ch_Set4) for each frequency band. Spectral peaks are observed at each stimulation frequency. The vertical bars indicate the standard errors of the spectral powers for each frequency.

Fig. 5 shows the grand-average spectral powers, as estimated by using the EEG data measured from 13 parieto-occipital channels (Ch_Set4) during visual stimulation for the 4 stimulation frequencies in the 3 frequency bands. Spectral peaks can be observed at the stimulation frequencies, regardless of the frequency band. Note that, among the 60 subdatasets (30 participants × 2 d), 10 datasets were excluded for this analysis because these datasets contained data showing extremely large SSVEP amplitudes at non-stimulation frequencies for some trials, and thus distorted the grand-average results (excluded datasets: Day 1 and Day 2 for S2; Day 2 for S10; Day 1 and Day 2 for S11; Day 2 for S13; Day 1 for S18; Day 1 for S20; Day 1 and Day 2 for S29).

**Figure 6:**
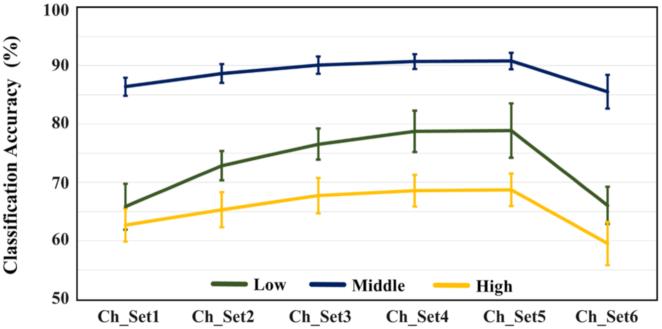
Changes in classification accuracy in terms of channel configuration for each stimulation frequency band. Eight channels attached to occipital areas (Ch_Set5) achieved the highest mean classification accuracy for all 3 frequency bands. The vertical bars indicate the standard deviations of classification accuracies for each channel set.

The classification accuracy results are presented for each stimulation frequency band in Fig. 6 with respect to the channel configuration shown in Fig. 2. The classification accuracy gradually increased as the number of channels used for classification was reduced to 8 channels in frontal areas (Ch_Set5), regardless of the frequency band; this means that occipital areas are most associated with visual information processing and thus provide the most discriminative information. However, the classification performance considerably deteriorated when only 3 electrodes (Ch_Set6: O1, O2, and Oz) were attached above occipital areas because less information was obtained.

**Figure 7:**
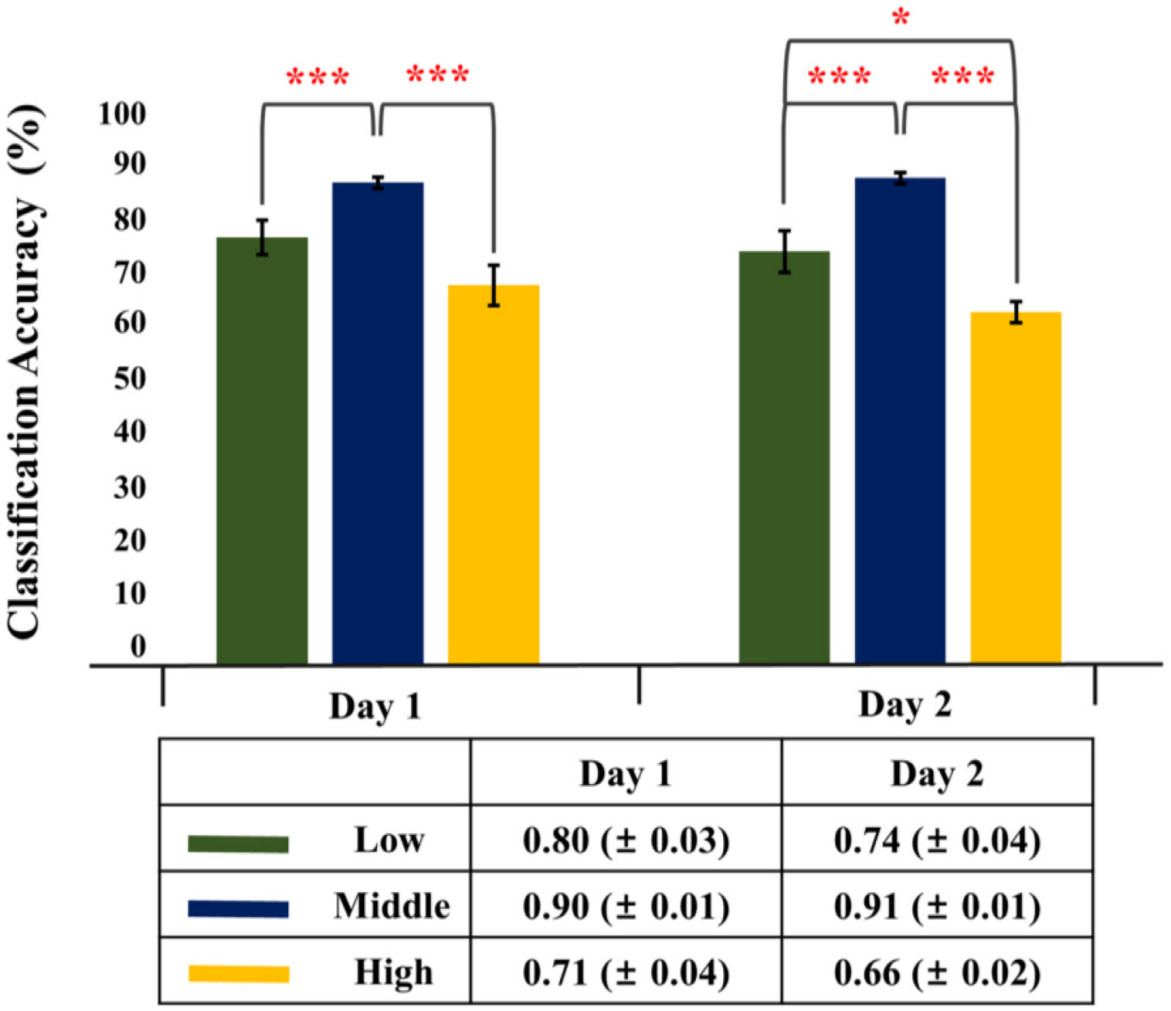
Mean classification accuracies for the 3 frequency bands for each day (RM-ANOVA: F(2, 29) = 19.87, *P* < 0.01; paired *t*-test Bonferroni-corrected *P* < 0.05: middle > low = high for the first day; RM-ANOVA: F(2, 29) = 23.09, *P* < 0.01; paired *t*-test Bonferroni-corrected *P* < 0.05: middle > low > high for the second day). The vertical bars indicate the standard deviations of classification accuracies for each frequency band.

Fig. 7 shows the mean classification accuracies for each frequency band on each experimental day; the results were obtained by using the best channel configuration (Ch_Set5) in terms of classification accuracy, as shown in Fig. 6. A similar statistical trend is shown for each experimental day; the mean classification accuracy for the middle-frequency band was significantly higher than those for the low- and high-frequency bands, and the mean classification accuracy for the low-frequency band was only found to be higher than that for the high-frequency band on the second day (RM-ANOVA: F(2, 29) = 19.87, *P* < 0.01; paired *t*-test Bonferroni-corrected *P* < 0.05: middle > low = high on the first day; RM-ANOVA: F(2, 29) = 23.09, *P* < 0.01; paired *t*-test Bonferroni-corrected *P* < 0.05: middle > low > high on the second day). No significant difference was observed between the 2 days with respect to the stimulation frequency band.

**Figure 8:**
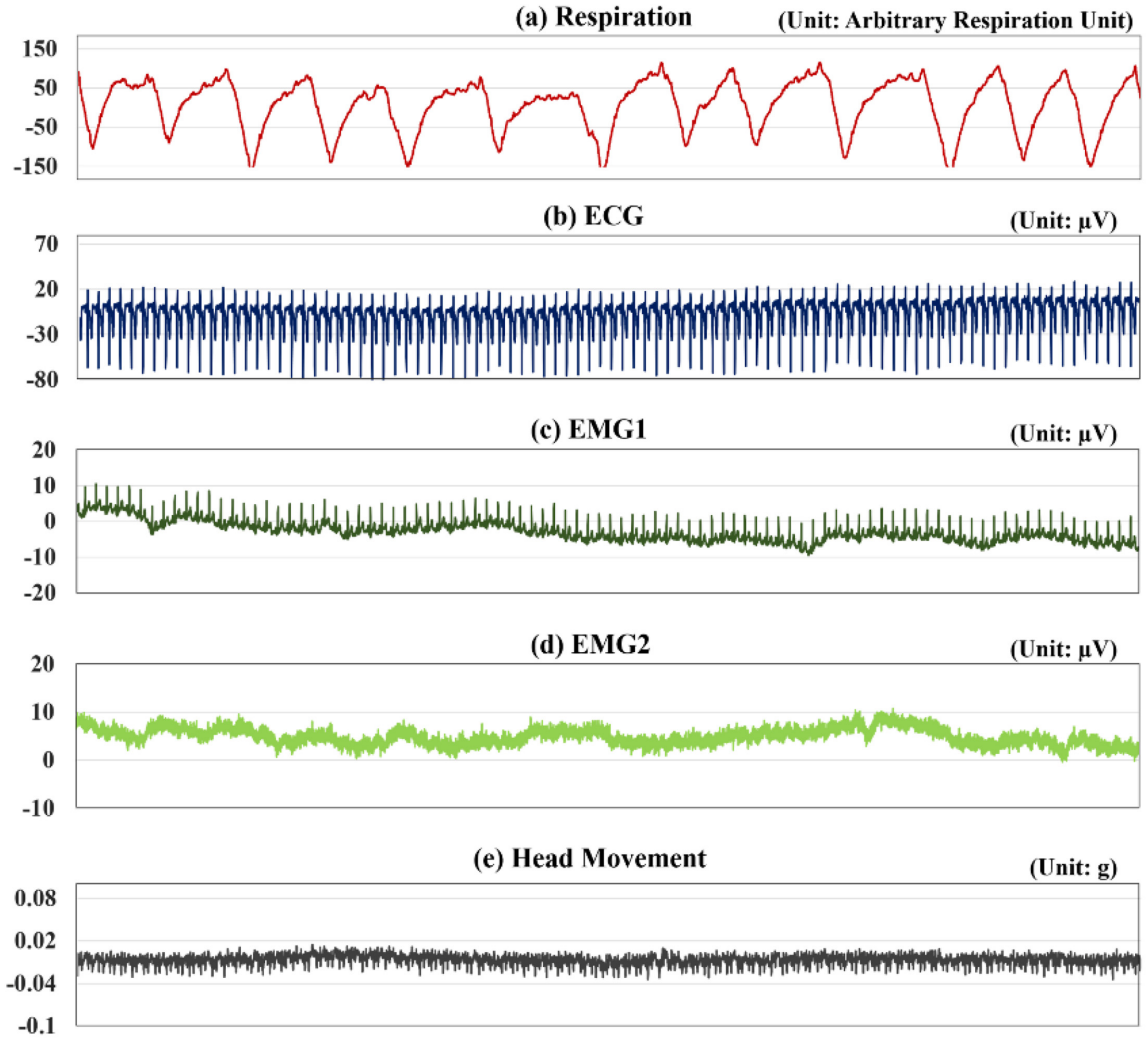
Examples of 5 types of physiological measurement data, with vendor-specific units: (a) respiration (arbitrary respiration unit), (b) ECG (μV), (c) EMG1 (left, posterior side of the neck) (μV), (d) EMG2 (right posterior side of the neck) (μV), and (e) head movement (*g* ≈ 9.81 m/s^2^).

Examples of the 5 types of physiological signals that were measured along with the EEG signals are presented in Fig.   8. Because the physiological data show high inter- and intra-participant variability, representative examples are provided for each of the 5 types of physiological data; detailed results are provided as 5 supplementary figures (Supplementary Figs. 1-5), and in 10 supplementary files. The example data were measured from S2 during their first trial, when the participant started to focus on an LED that was modulated at 5 Hz; the duration was 60 s. In particular, 13 breaths and 93 heartbeats were clearly observed over the 60-s period in the respiratory (Fig. 8a) and ECG data (Fig. 8b), respectively; these numbers are within the normal ranges for the adult respiratory rate (12–18) [40] and heart rate (60–100) [41]. The 2 sets of example EMG data (Fig. 8c and d) and example head motion (Fig. 8e) data show that no significant movement was made; heartbeats were also observed in both sets of EMG data (Fig. 8c and d). Most participants showed similar trends for each corresponding type of physiological signal, with the exception of a few cases (see Supplementary Figs. 1-5 and Supplementary Files 1-10) .

### Reuse potential

Although the SSVEP is one of the most widely used BCI paradigms [42], publicly available SSVEP BCI datasets are still scarce. In this study, we created multi-band and multi-day SSVEP BCI datasets for the first time and validated their feasibility through SSVEP spectral power and classification analyses. All of the results were found to be consistent with those reported in previous studies; particularly, SSVEP responses were mainly observed near occipital areas, with spectral peaks occurring at the stimulation frequencies regardless of the stimulation frequency band; additionally, the classification accuracy for the middle-frequency band was higher than those for the low- and high-frequency band [25, 43]. Our multi-band SSVEP datasets can be used to investigate participant-specific stimulation frequencies because they enable comparison of the characteristics of the SSVEPs evoked in each of the 3 frequency bands, which can thus be used to improve the performance of SSVEP-based BCIs. Additionally, the multi-day SSVEP datasets can be used to develop advanced solutions for session-to-session (day-to-day) transfer problems because they provide data that can be used to investigate how SSVEP characteristics can differ on different days, the analysis of which can be used to enhance the reliability of SSVEP-based BCIs.

All other physiological signals that were simultaneously measured with the EEG signals also yielded reasonable results, even though only a representative example of each type of signal result was shown because there was high inter- and intraparticipant variability. The physiological data can be used not only to investigate the relationship between brain activity and various physiological variables but also to develop artifact correction methods for SSVEPs. Particularly for the latter case, IMU and EMG data can be used to detect head/neck movements that would degrade the quality of EEG data and then to correct them based on advanced algorithms.

## Availability of Supporting Data and Materials

The data supporting this paper, including the EEG and other physiological datasets, and the questionnaire results, are available in the *GigaScience* database, GigaDB [44].

## Additional files

**Supplementary Figure 1**. Respiratory rates of each participant recorded on 2 different days. For detailed information, refer to Supplementary Files 1 and 2 (Respiration_Day1.xlxs and Respiration_Day2.xlsx).

**Supplementary Figure 2**. Heart rates of each participant recorded on 2 different days. For detailed information, refer to Supplementary Files 3 and 4 (ECG_Day1.xlxs and ECG_Day2.xlsx).

**Supplementary Figure 3**. Mean trial EMG values for each participant estimated using the EMG1 channel data. For detailed information, refer to Supplementary Files 5 and 6 (EMG1_Day1.xlxs and EMG1_Day2.xlsx).

**Supplementary Figure 4**. Mean trial EMG values for each participant estimated using the EMG2 channel data. For detailed information, refer to Supplementary Files 7 and 8 (EMG2_Day1.xlxs and EMG2_Day2.xlsx).

**Supplementary Figure 5**. Mean trial IMU values for each participant and each day. For detailed information, refer to Supplementary Files 9 and 10 (HeadMovement_Day1.xlxs and HeadMovement_Day2.xlsx).

Supplementary Files.

Supplementary File 1. Detailed information for Supplmentary Figure 1 (Respiration_Day1.xlxs).

Supplementary File 2. Detailed information for Supplmentary Figure 1 (Respiration_Day2.xlxs).

Supplementary File 3. Detailed information for Supplmentary Figure 2 (ECG_Day1.xlxs).

Supplementary File 4. Detailed information for Supplmentary Figure 2 (ECG_Day2.xlxs).

Supplementary File 5. Detailed information for Supplmentary Figure 3 (EMG1_Day1.xlxs).

Supplementary File 6. Detailed information for Supplmentary Figure 3 (EMG1_Day2.xlxs).

Supplementary File 7. Detailed information for Supplmentary Figure 3 (EMG2_Day1.xlxs).

Supplementary File 8. Detailed information for Supplmentary Figure 3 (EMG2_Day2.xlxs).

Supplementary File 9. Detailed information for Supplmentary Figure 3 (HeadMovement_Day1.xlxs).

Supplementary File 10. Detailed information for Supplmentary Figure 3 (HeadMovement_Day2.xlxs).

giz133_GIGA-D-19-00116_Original_Submission

giz133_GIGA-D-19-00116_Revision_1

giz133_GIGA-D-19-00116_Revision_2

giz133_Response_to_Reviewer_Comments_Original_Submission

giz133_Response_to_Reviewer_Comments_Revision_1

giz133_Reviewer_1_Report_Original_Submission Dan Zhang -- 5/15/2019 Reviewed

giz133_Reviewer_1_Report_Revision_1 Dan Zhang -- 8/29/2019 Reviewed

giz133_Reviewer_2_Report_Original_Submission Hohyun Cho -- 5/28/2019 Reviewed

giz133_Reviewer_2_Report_Revision_1 Hohyun Cho -- 9/11/2019 Reviewed

giz133_Supplemental_Files

## Abbreviations

BCI: brain-computer interface; ECG: electrocardiography; EEG: electroencephalogram; EMG: electromyography; ERP: event-related potential; IMU: inertial measurement unit; LCD: liquid crystal display; LED: light-emitting diode; RM-ANOVA: repeated-measures analysis of variance; SNR: signal-to-noise ratio; SSVEP: steady-state visual-evoked potential.

## Ethical Approval

This study was approved by the Institutional Review Board of Kumoh National Institute of Technology (No. 6250).

## Competing Interests

The authors declare that they have no competing interests.

## Funding

This work was supported by the Institute for Information & Communications Technology Planning & Evaluation (IITP) grant funded by the Korean government (No. 2017-0-00451; Development of BCI based Brain and Cognitive Computing Technology for Recognizing User's Intentions using Deep Learning).

## Authors' Contributions

G.-Y.C. and H.-J.H. designed the experiment, Y.-J.J. implemented an SSVEP stimulator, G.-Y.C. acquired the data, G.-Y.C., C.-H.H., and Y.-J.J. performed data analysis, and H.-J.H. supervised this study. All authors wrote and reviewed the manuscript.

## References

[1] Pfurtscheller G, Flotzinger D, Kalcher J Brain-computer interface-a new communication device for handicapped persons. J Microcomput Appl. 1993;16(3):293–9.

[2] Wolpaw JR, Birbaumer N, McFarland DJ, et al. Brain-computer interfaces for communication and control. Clin Neurophysiol. 2002;113(6):767–91. 12048038 10.1016/s1388-2457(02)00057-3

[3] Nicolas-Alonso LF, Gomez-Gil J Brain computer interfaces, a review. Sensors. 2012;12(2):1211–79. 22438708 10.3390/s120201211 PMC3304110

[4] Decety J, Ingvar DH Brain structures participating in mental simulation of motor behavior: A neuropsychological interpretation. Acta Psychol. 1990;73(1):13–34. 10.1016/0001-6918(90)90056-l 2180254

[5] Jeannerod M, Frak V Mental imaging of motor activity in humans. Curr Opin Neurobiol. 1999;9(6):735–9. 10607647 10.1016/s0959-4388(99)00038-0

[6] Pfurtscheller G, Brunner C, Schlögl A, et al. Mu rhythm (de) synchronization and EEG single-trial classification of different motor imagery tasks. Neuroimage. 2006;31(1):153–9. 16443377 10.1016/j.neuroimage.2005.12.003

[7] Pfurtscheller G, Neuper C Motor imagery and direct brain-computer communication. Proc IEEE. 2001;89(7):1123–34.

[8] Blankertz B, Muller K-R, Curio G, et al. The BCI competition 2003: progress and perspectives in detection and discrimination of EEG single trials. IEEE Trans Biomed Eng. 2004;51(6):1044–51. 15188876 10.1109/TBME.2004.826692

[9] Blankertz B, Muller K-R, Krusienski DJ, et al. The BCI competition III: Validating alternative approaches to actual BCI problems. IEEE Trans Neural Syst Rehabil Eng. 2006;14:(2):153–9. 16792282 10.1109/TNSRE.2006.875642

[10] Cho H, Ahn M, Ahn S, et al. EEG datasets for motor imagery brain computer interface. Gigascience. 2017;6(7), doi:10.1093/gigascience/gix034. PMC5493744 28472337

[11] Sajda P, Gerson A, Muller K-R, et al. A data analysis competition to evaluate machine learning algorithms for use in brain-computer interfaces. IEEE Trans Neural Syst Rehabil Eng. 2003;11(2):184–5. 12899269 10.1109/TNSRE.2003.814453

[12] Tangermann M, Muller K-R, Aertsen A, et al. Review of the BCI competition IV. Front Neurosci. 2012;6:55. 22811657 10.3389/fnins.2012.00055 PMC3396284

[13] Shin J, von Luhmann A, Blankertz B, et al. Open access dataset for EEG+ NIRS single-trial classification. IEEE Trans Neural Syst Rehabil Eng. 2017;25(10):1735–45. 10.1109/TNSRE.2016.2628057 27849545

[14] BNCI Horizon 2020 Datasets. http://bnci-horizon-2020.eu/database/data-sets. Accessed 25 March 2019.

[15] Farwell LA, Donchin E Talking off the top of your head: Toward a mental prosthesis utilizing event-related brain potentials. Electroencephalogr Clin Neurophysiol. 1988;70(6):510–23. 2461285 10.1016/0013-4694(88)90149-6

[16] Sakurada T, Kawase T, Takano K, et al. A BMI-based occupational therapy assist suit: Asynchronous control by SSVEP. Front Neurosci. 2013;7:172. 24068982 10.3389/fnins.2013.00172 PMC3779864

[17] Kwak N-S, Müller K-R, Lee S-W A lower limb exoskeleton control system based on steady state visual evoked potentials. J Neural Eng. 2015;12(5):056009. 26291321 10.1088/1741-2560/12/5/056009

[18] Gollee H, Volosyak I, McLachlan AJ, et al. An SSVEP-based brain-computer interface for the control of functional electrical stimulation. IEEE Trans Biomed Eng. 2010;57(8):1847–55. 20176528 10.1109/TBME.2010.2043432

[19] Hwang H-J, Lim J-H, Jung Y-J, et al. Development of an SSVEP-based BCI spelling system adopting a QWERTY-style LED keyboard. J Neurosci Methods. 2012;208(1):59–65. 22580222 10.1016/j.jneumeth.2012.04.011

[20] Lim J-H, Lee J-H, Hwang H-J, et al. Development of a hybrid mental spelling system combining SSVEP-based brain-computer interface and webcam-based eye tracking. Biomed Signal Process Control. 2015;21:99–104.

[21] Wang Y, Chen X, Gao X, et al. A benchmark dataset for SSVEP-based brain-computer interfaces. IEEE Trans Neural Syst Rehabil Eng. 2017;25(10):1746–52. 27849543 10.1109/TNSRE.2016.2627556

[22] Lee M-H, Kwon O, Kim Y-J, et al. EEG dataset and OpenBMI toolbox for three BCI paradigms: An investigation into BCI illiteracy. Gigascience. 2019;8(5):giz002. 30698704 10.1093/gigascience/giz002 PMC6501944

[23] Herrmann CS Human EEG responses to 1–100 Hz flicker: resonance phenomena in visual cortex and their potential correlation to cognitive phenomena. Exp Brain Res. 2001;137(3-4):346–53. 11355381 10.1007/s002210100682

[24] Galloway N Human brain electrophysiology: Evoked potentials and evoked magnetic fields in science and medicine. Br J Ophthalmol. 1990;74(4):255.

[25] Volosyak I, Valbuena D, Luth T, et al. BCI demographics II: How many (and what kinds of) people can use a high-frequency SSVEP BCI?. IEEE Trans Neural Syst Rehabil Eng. 2011;19(3):232–9. 21421448 10.1109/TNSRE.2011.2121919

[26] Sakurada T, Kawase T, Komatsu T, et al. Use of high-frequency visual stimuli above the critical flicker frequency in a SSVEP-based BMI. Clin Neurophysiol. 2015;126(10):1972–8. 25577407 10.1016/j.clinph.2014.12.010

[27] Cho H, Ahn M, Kim K, et al. Increasing session-to-session transfer in a brain-computer interface with on-site background noise acquisition. J Neural Eng. 2015;12(6):066009. 26447843 10.1088/1741-2560/12/6/066009

[28] Krauledat M, Tangermann M, Blankertz B, et al. Towards zero training for brain-computer interfacing. PLoS One. 2008;3(8):e2967. 18698427 10.1371/journal.pone.0002967 PMC2500157

[29] Samek W, Meinecke FC, Müller K-R Transferring subspaces between subjects in brain-computer interfacing. IEEE Trans Biomed Eng. 2013;60(8):2289–98. 23529075 10.1109/TBME.2013.2253608

[30] Zhu D, Bieger J, Molina GG, et al. A survey of stimulation methods used in SSVEP-based BCIs. Comput Intell Neurosci. 2010;2010:12. 10.1155/2010/702357 PMC2833411 20224799

[31] Hwang HJ, Kim DH, Han CH, et al. A new dual-frequency stimulation method to increase the number of visual stimuli for multi-class SSVEP-based brain–computer interface (BCI). Brain Res. 2013;1515:66–77. 23587933 10.1016/j.brainres.2013.03.050

[32] Cheng M, Gao X, Gao S et al. Design and implementation of a brain-computer interface with high transfer rates. IEEE Trans Biomed Eng. 2002;49(10):1181–6. 12374343 10.1109/tbme.2002.803536

[33] Park SA, Hwang HJ, Lim JH, et al. Evaluation of feature extraction methods for EEG-based brain–computer interfaces in terms of robustness to slight changes in electrode locations. Med Biol Eng Comput. 2013;51(5):571–9. 23325145 10.1007/s11517-012-1026-1

[34] González-Franco M, Yuan P, Zhang D, et al. Motor imagery based brain-computer interface: A study of the effect of positive and negative feedback, Conf Proc IEEE Eng Med Biol Soc 2011; 6323–6. 22255784 10.1109/IEMBS.2011.6091560

[35] Pfurtscheller G, Solis Escalante T, Barry RJ et al. Brisk heart rate and EEG changes during execution and withholding of cue-paced foot motor imagery. Front Hum Neurosci. 2013;7:379. 23908614 10.3389/fnhum.2013.00379 PMC3726939

[36] Gwin JT, Gramann K, Makeig S et al. Removal of movement artifact from high-density EEG recorded during walking and running. J Neurophysiol. 2010;103:(6):3526–34. 20410364 10.1152/jn.00105.2010 PMC3774587

[37] O'Regan S, Faul S, Marnane W., Automatic detection of EEG artefacts arising from head movements using EEG and gyroscope signals, Med Eng Phys, 2013; 35:(7): 867–74. 23018030 10.1016/j.medengphy.2012.08.017

[38] Vialatte F-B, Maurice M, Dauwels J, et al. Steady state visual evoked potentials in the delta range (0.5–5 Hz). In: Köppen M, Kasabov N, Coghill G, eds. Proceedings of 15th International Conference on Advances in Neuro-information Processing. Springer; 2009:400–7.

[39] Lin Z, Zhang C, Wu W, et al. Frequency recognition based on canonical correlation analysis for SSVEP-based BCIs. IEEE Trans Biomed Eng. 2006;53:(12):2610–4. 17152442 10.1109/tbme.2006.886577

[40] Barrett KE, Barman SM, Boitano S, et al. Ganong's Review of Medical Physiology. New York, NY: McGraw-Hill Medical; 2009: 23.

[41] Aladin AI, Whelton SP, Al-Mallah MH, et al. Relation of resting heart rate to risk for all-cause mortality by gender after considering exercise capacity (the Henry Ford exercise testing project). Am J Cardiol. 2014;114(11):1701–6. 25439450 10.1016/j.amjcard.2014.08.042

[42] Hwang H-J, Kim S, Choi S et al. EEG-based brain-computer interfaces: a thorough literature survey. Int J Hum-Comput Interact. 2013; 29:(12): 814–26.

[43] Müller SMT, Diez PF, Bastos-Filho TF et al. Robotic wheelchair commanded by people with disabilities using low/high-frequency SSVEP-based BCI. In: Jaffray D, ed. World Congress on Medical Physics and Biomedical Engineering, Toronto, Ontario, Canada. Cham: Springer, 2015: 1177–80.

[44] Choi G-Y, Han C-H, Jung Y-J, Hwang H-J. Supporting data for .“A multi-day and multi-band dataset for steady-state visual evoked potenial-based brain-computer interface”. GigaScience Database. 2019 10.5524/100660. PMC6876666 31765472

