# Supplementary material for: A multi-day and multi-band dataset for a steady-state visual-evoked potential–based brain-computer interface
Source: Gigascience. 2019 Nov 25;8(11):giz133. doi: 10.1093/gigascience/giz133 (PMC6876666; doi:10.1093/gigascience/giz133)
Supplement: giz133_GIGA-D-19-00116_Original_Submission [file giz133_giga-d-19-00116_original_submission.pdf]

GigaScience

A multi-day and multi-band dataset for steady-state visual evoked potential-based brain-computer interface

--Manuscript Draft--

**Manuscript Number:**	GIGA-D-19-00116
**Full Title:**	A multi-day and multi-band dataset for steady-state visual evoked potential-based brain-computer interface
**Article Type:**	Data Note
**Funding Information:**	Institute for Information and Information & Communications Technology Planning & Evaluation (2017-0-00451) — Prof. Han-Jeong Hwang
**Abstract:**	Background<br><br>Steady-state visual evoked potential (SSVEP) is a brain response to a visual stimulus modulated at a certain frequency, and it has been widely used in electroencephalography (EEG)-based brain-computer interface (BCI) research. However, accessible SSVEP datasets for BCI have been rarely published. In this study, we provide a new SSVEP dataset measured from thirty subjects for two different days, which complements existing SSVEP datasets for the three following aspects: i) multi-band SSVEP datasets are provided by using all possible three frequency bands (low, middle, and high) used for SSVEP stimulation, ii) multi-day datasets are provided, and iii) EEG datasets are provided along with physiological data, such as respiration, electrocardiography, electromyography, head motion and body temperature.<br><br>Findings<br><br>To validate our dataset, we estimated spectral powers and classification performance for the EEG (SSVEP) datasets, and showed example time-series data for physiological data. Strong SSVEP responses were observed at stimulation frequencies, and the mean classification performance of the middle frequency band was significantly higher than that of the low- and high-frequency bands. Other physiolgical data also showed reasonalbe results.<br><br>Conclusions<br><br>Our multi-band and multi-day SSVEP datasets can be used to optimize stimulation frequencies by simultaneously investigating the characteristics of SSVEPs evoked in each of the three frequency bands and solve session-to-session (day-to-day) transfer issues by investigating non-stationarity of SSVEPs measured from different days, respectivley. Also, auxiliary physiological data can be used to explore the relation between SSVEP characteristics and physiological conditions, thereby providing useful information in optimizing experimental paradigms to attain high performance.
**Corresponding Author:**	Han-Jeong Hwang<br>Kumoh National Institute of Technology<br>Gumi, Gyeongsangbuk-do KOREA, REPUBLIC OF
**Corresponding Author Secondary Information:**	
**Corresponding Author's Institution:**	Kumoh National Institute of Technology
**Corresponding Author's Secondary Institution:**	
**First Author:**	Ga-Young Choi
**First Author Secondary Information:**	
**Order of Authors:**	Ga-Young Choi

Powered by Editorial Manager® and ProduXion Manager® from Aries Systems Corporation

	Chang-Hee Han
	Young-Jin Jung
	Han-Jeong Hwang
**Order of Authors Secondary Information:**	
**Additional Information:**	
**Question**	**Response**
Are you submitting this manuscript to a special series or article collection?	No
**Experimental design and statistics**<br><br>Full details of the experimental design and statistical methods used should be given in the Methods section, as detailed in our Minimum Standards Reporting Checklist. Information essential to interpreting the data presented should be made available in the figure legends.<br><br>Have you included all the information requested in your manuscript?	Yes
**Resources**<br><br>A description of all resources used, including antibodies, cell lines, animals and software tools, with enough information to allow them to be uniquely identified, should be included in the Methods section. Authors are strongly encouraged to cite Research Resource Identifiers (RRIDs) for antibodies, model organisms and tools, where possible.<br><br>Have you included the information requested as detailed in our Minimum Standards Reporting Checklist?	Yes
**Availability of data and materials**<br><br>All datasets and code on which the conclusions of the paper rely must be either included in your submission or deposited in publicly available repositories (where available and ethically	No

appropriate), referencing such data using a unique identifier in the references and in the “Availability of Data and Materials” section of your manuscript.<br><br>Have you have met the above requirement as detailed in our Minimum Standards Reporting Checklist?	
If not, please give reasons for any omissions below.<br><br>as follow-up to "**Availability of data and materials**<br><br>All datasets and code on which the conclusions of the paper rely must be either included in your submission or deposited in publicly available repositories (where available and ethically appropriate), referencing such data using a unique identifier in the references and in the “Availability of Data and Materials” section of your manuscript.<br><br>Have you have met the above requirement as detailed in our Minimum Standards Reporting Checklist?<br><br>"	As far as I know, we can make our data open to reviewers after an initial check. Thus, I will provide our data after an initial check.

Powered by Editorial Manager® and ProduXion Manager® from Aries Systems Corporation

Manuscript

Click here to download Manuscript GigaSicence_manuscript_20190405.docx

A multi-day and multi-band dataset for steady-state visual evoked potential-based brain-computer interface

Ga-Young Choi[1], Chang-Hee Han[2], Young-Jin Jung[3], Han-Jeong Hwang[1,*]

E-mail: cgy326@naver.com, zeros8706@naver.com, microbme@outlook.com, h2j@ kumoh.ac.kr

[1]Department of Medical IT Convergence Engineering, Kumoh National Institute of Technology, Gumi 39177, Republic of Korea

[2]Machine Learning Group, Berlin Institute of Technology (TU Berlin), 10623 Berlin, Germany

[3]Department of Radiological Science, Dongseo University, Busan 47011, Republic of Korea

Number of Pages: 22

Number of Figures: 7

Number of Tables: 2

Corresponding Author Information:

Name: Han-Jeong Hwang

Address: Kumoh National Institute of Technology, 39177, Gumi-si, Gyeongsangbuk-do, Republic of Korea

Tel.: +82-054-478-7783

E-mail: h2j@kumoh.ac.kr

Abstract

Background: Steady-state visual evoked potential (SSVEP) is a brain response to a visual stimulus modulated at a certain frequency, and it has been widely used in electroencephalography (EEG)-based brain-computer interface (BCI) research. However, accessible SSVEP datasets for BCI have been rarely published. In this study, we provide a new SSVEP dataset measured from thirty subjects for two different days, which complements existing SSVEP datasets for the three following aspects: i) multi-band SSVEP datasets are provided by using all possible three frequency bands (low, middle, and high) used for SSVEP stimulation, ii) multi-day datasets are provided, and iii) EEG datasets are provided along with physiological data, such as respiration, electrocardiography, electromyography, head motion and body temperature.

Findings: To validate our dataset, we estimated spectral powers and classification performance for the EEG (SSVEP) datasets, and showed example time-series data for physiological data. Strong SSVEP responses were observed at stimulation frequencies, and the mean classification performance of the middle frequency band was significantly higher than that of the low- and high-frequency bands. Other physiolgical data also showed reasonalbe results.

Conclusions: Our multi-band and multi-day SSVEP datasets can be used to optimize stimulation frequencies by simultaneously investigating the characteristics of SSVEPs evoked in each of the three frequency bands and solve session-to-session (day-to-day) transfer issues by investigating non-stationarity of SSVEPs measured from different days, respectivley. Also, auxiliary physiological data can be used to explore the relation between SSVEP characteristics and physiological conditions, thereby providing useful information in optimizing experimental paradigms to attain high performance.

Key words: steady-state visual evoked potential (SSVEP); brain-computer interface (BCI); electroenchephalography (EEG); physiologcal data

Data Description

Background and purpose

Brain-computer interface (BCI) is a non-muscular communication method using brain activity, such as electroencephalograhy (EEG), for handicapped individuals who are unable to voluntarily control their bodies [1, 2]. Two approaches have been employed to develop EEG-based BCIs whether external stimuli are used or not [3]; an endogenous BCI uses mental imagery tasks while an exogenous BCI uses external stimuli in order to induce and evoke certain brain patterns, respectivley.

A representative endogenous BCI paradigm is motor imagery that is defined as mental simulation of motor behaviors, e.g., left/right hand movement [4, 5]. Thanks to event-related (de)synchroniztion (ERD/S) phenomenon, different motor imagery tasks can be discriminated using machine learning techniques, and then it can be used for BCI purpose [6. 7]. So far, a large number of motor imagery BCI datasets have been published [8-14], and they have contributed to significant advancement in the BCI community. Other endogenous BCI datasets are also available, such as slow cortical potential (SCP), readiness potential [8], and mental arithmetic [13, 14].

There are two representative exogenous BCI paradigms: event-related potential (ERP) and steady-state visual evoked potential (SSVEP). ERP is a time-locked brain response evoked in response to specific visual, auditory, and tactile stimuli while SSVEP is a period brain response to a visual stimulus modulated at a certain frequency. ERP has been mostly used in developing row/column matrix spellers [15], while SSVEP has been used in developing a variety of BCI applications, such as control of a robotic arm [16], an exoskeleton [17], and a functional

electrical stimulation (FES) [18], and spelling of a character [19, 20]. Many ERP BCI datasets have become publicly available since the first ERP BCI dataset was publised in 2003 [8]. However, it was not until 2017 that a freely accessible SSVEP BCI dataset was published for the first time [21], and it was followed by the second one in 2019 even though the SSVEP paradigm has been widely used in BCI research due to high performance with little user training [22].

Due to the lack of SSVEP BCI datasets compared to those based on other BCI paradigms, it would be benificial for BCI researchers to provide a new SSVEP BCI dataset that can complement the existing SSVEP BCI datasets. The first SSVEP dataset was measured from 35 subjects with a 40-target BCI speller, where SSVEP stimulation frequencies ranged from 8 to 15.8 Hz with a span of 0.2 Hz [21]. The second SSVEP dataset was acquired from 54 subjects with a 4-class BCI system over two sessions, where 5.45, 6.67, 8.57, and 12 Hz were used as stimluation frequencies [22].

In this study, we provide a new SSVEP BCI dataset that can contribute to SSVEP-based BCI research in three main aspects. First, our SSVEP dataset consists of three sub-datasets that are measured with three different frequency bands, respectively: low (1 – 12 Hz), middle (12 – 30 Hz), and high (30 – 60 Hz) frequency band. It is well documented that SSVEP is elicited in a wide rage of frequencies, rainging from 1 to 90 Hz [23], and the frequencies can be divided into three sub-frequency bands as mentioned above (low, middle, and high) [24]. The two previous SSVEP datasets were acquired using stimulation frequencies in certain frequency bands, i.e., 8 – 15.8 Hz in the low and middle frequeny bands [21] and 5.45 – 12 Hz in the low frequency band [22]. Considering that choie of the stimluation frequency band is one of the important factors that significantly affect the performance of SSVEP-based BCIs [25], the

characteristics of SSVEPs evoked in each of the three frequency bands should be simultaneously investigated in terms of their signal-to-noise ratio (SNR) and classification performance. In particular, the high frequency band has been recevied increasing attention as an alternative to the low and middle frequency bands due to less visual fatigue despite relatively low performance [26]. However, no SSVEP BCI studies have provided available datasets for the high frequency band. Thus, it is necessray to provide an SSVEP dataset recorded using the high frequency band along with those recorded using the low and middle frequency bands to simultaneously investigate the mentioned issues. Our SSVEP dataset can make it possible because it was measured by using the three frequency bands independently from same subjects. Secondly, we provide a multi-session (day) dataset that was recorded over two different days from same subjects. Thus, our SSVEP dataset can be used to study session-to-session transfer which is an challening issue in BCI research [27-29]. A multi-session SSVEP dataset was also provided in [22], but which was acquried on the same day with a short break (i.e., 3 min). Therefore, our dataset can be more usefully used to get profound insights into the non-stationary nature of EEG, thereby providing useful solutions to overcome session-to-session (day-to-day) transfer issues. Finally, we provide other physiological datasets, which were not presented in the two previous SSVEP datasets [22, 23], along with the EEG dataset to check changes in physiolgical conditions of subjects during the experiment, such as respiration, electrocardiography (ECG), neck electromyography (EMG), head motion, and body temperature. The auxiliary physiological data can be used to explore the relationship between SSVEP characteristics (e.g., SNR) and physiological conditions, thereby providing useful information in designing experimental paradigms to attain high performance.

In order to provide a novel SSVEP BCI dataset complementary to the previous two SSVEP

BCI datasets, we designed a 4-class SSVEP paradigm as that used for acquring the second SSVEP BCI dataset [22]. Three sets of four stimulation frequencies were employed for the low, middle, and high frequency bands, respectively. The SSVEP BCI dataset was acquired from thiry subjects from two different days. For data validation, we applied a standard analysis method to our SSVEP dataset, and provide baseline results along with all of the mentioned physiological datasets in this study.

Experimental design

Subjects

Thirty subjects (9 females and 21 males; 23.8 ± 1.3 years) were recruited for this study. They had no history of psychiatric diseases that might affect research results. They were given the details of experimental procedures, and signed an informed consent for study participation and anonymously data open to the public before the experiment. Adequate reimbursement was provided for their participation after the experiment. This study was approved by the Institutional Review Board (IRB) of Kumoh National Institute of Technology (No.6250), and was conducted in accordance with the principles of the declaration of Helsinki.

Stimulator

An SSVEP stimulator was made of two square styrofoams, a black thick paper, an opaque film, four LEDs and an LED controller. We first cut out five parts of a styrofoam, four of which was 3 x 3 cm for LED display, and the other was 9 x 5.5 cm to show an instruction during the experiment (Figure 1(a)). After that, we inserted four LEDs into the four square wholes of 3 x 3 cm (part number: T03WC01; operating current: 20 mA; viewing angle: θ/2 = 100º; luminous

intensity: 2,000 mcd; emitting color: white), and attached another styrofoam to the back of the first styrofoam. The front part of the stimulator was covered with an opaque film to diffuse the light, and then we attached a black paper with five square wholes that were exactly matched to those in the front styrofoam on to the opaque film for better visibility. The stimulator was attached to a 21-inch LCD monitor, and an instruction which LED the subject should focus on was presented with an arrow from the monitor through the center square whole of 9 x 5.5 cm. A schematic diagram of the SSVEP stimulator is shown in figure 1(a). The distance between each LED and an instruction arrow presented on the center of the monitor was 17 cm. In order to control the four LEDs, we used a LAUNCHXL-F28027 Board powered by C2000 MCU (Texas Instrument).

As mentioned above, three different frequency bands were independently used for SSVEP stimulation to get a multi-band SSVEP dataset in this study. Three sets of four stimulation frequencies selected for each frequency band were as follows: 5, 5.5, 6, and 6.5 Hz for the low frequency band; 21, 21.5, 22, 22.5 Hz for the middle frequency band; 40, 40.5, 41, and 41.5 Hz for the high frequency band. We assigned four stimulation frequencies to four LEDs, depending on the stimulation frequency band, as shown in figure 1(b).

[Figure 1 here]

Experimental paradigm

During the experiment, the subjects sat on a comfortable arm chair in front of the SSVEP stimulator attached on a 21-inch monitor away from about 1 m, and they were instructed to remain relaxed without any movements. Note that all instructions were presented on the center of the monitor and the subjects could perceive them through the center whole of the stimulator.

For each trial, a blank screen was presented for 5 s, and then an arrow indicating one of the four LEDs was presented for 6 s, during which the subject was asked to gaze at a target LED according to the direction of an arrow. After that, a white cross mark ('+') was presented for 6 s as a short break for the next trial. A short beep sound was also presented for every transition of visual stimuli in order to lead to more explicit attention of the subjects. The direction of an arrow was randomly presented 20 times (20 trials) for each direction, resulting in a total of 80 trials, which was repeated for each frequency band (low, middle, and high), respectively. To avoid excessive fatigue, a break was regularly given to each subject after acquiring every 40 trials (defined as one session) for at least 5 min, and also irregularly whenever the subjects wanted during the experiment. Each subject performed six sessions of an SSVEP experiment (2 sessions x 3 frequency bands), which was conducted twice for different days with an interval of at least one day. The three stimulation frequency bands were alternatively used for counterbalancing between subjects. In particular, all possible order combinations of the three frequency bands were as follows: (low-middle-high), (low-high-middle), (middle-low-high), (middle-high-low), (high-low-middle), (high-middle-low). Each order was randomly assigned to five subjects, respectively (6 orders x 5 subjects = 30 subjects), and a same order was used for two days once an order was assigned to each subject in the first day experiment. The experiment lasted about 2 h, including EEG preparation for each day.

Data recording

EEG data were measured using a BrainAmp EEG amplifier (Brain products, GmbH Ltd, Germany) with a sampling rate of 1,000 Hz, where the ground and reference electrodes were attached on Fpz and FCz, respectively (figure 2). We used thirty-three active electrodes

mounted based on the international 10-10 system to measure EEGs (FP1, FP2, AF4, AF3, F5, Fz, FC1, FC5, F6, FC2, FC6, C4, Cz, C3, CP1, CP2, CP6, P8, P4, Pz, POz, PO4, PO8, O2, Oz, O1, PO3, P3, CP5, P7, PO7, T7, and T8), where electrodes were more densely mounted around occipital areas than the others because SSVEP is mainly originated from the occipital lobe.

[Figure 2 here]

We also measured various bio-signals simultaneously with the EEG data to check changes in physiological conditions, such as respiration, ECG, neck EMG, head motion, and body temperature. In order to measure these physiological data, we attached a respiratory belt to the chest, three ECG sensors on lead-I position (Einthoven's triangle), two EMG sensors on the right and left side of the neck, an inertial measurement unit (IMU) sensor on the top of the head between Cz and CPz, and a temperature sensor under the armpit, respectively. The same amplifier used for measuring EEG was used for recording the physiological data with a same sampling rate of 1,000 Hz, and thus all of the measured data were synchronized.

Data format and structure

Because data analysis was performed using Matlab R2013b (MathWorks, Natick, MA, USA), we provide our dataset in the form of Matlab files (.mat). Each data folder named as subject initial (e.g., S1) has two sub-folders for two sub-datasets measured from two different days (i.e., day1 and day2). Each sub-folder has *cnt* and *mrk* files, which contain continuous time series data for all physiological measurement (*cnt*) and the trigger information of the corresponding data (*mrk*), respectively. The *cnt* and *mrk* files have suffixes corresponding to

three frequency bands and session numbers. For example, *cnt*_Low(1) means times series data measured using the low frequency band for SSVEP stimulation in the first session. Thus, the sub-folder of each subject contains the following six pairs of *cnt* and *mrk* files: *cnt*_Low(1), *mrk*_Low(1), *cnt*_Low(2), *mrk*_Low(2), *cnt*_Middle(1), *mrk*_Middle(1), *cnt*_Middle(2), *mrk*_Middle(2), *cnt*_High(1), *mrk*_High(1), *cnt*_High(2), and *mrk*_High (2). All data were down-sampled to 200 Hz when converting the raw data into Matlab compatible files. Table 1 shows all data files contained in each sub-folder.

Table 1. Data format. Each data folder of all subjects has two sub-folders for two sub-datasets measured from two different days, and each sub-folder has six pairs of *cnt* and *mrk* files (shown below) according to stimulation frequency band and session number.

Frequency Band	Stimulation Frequency	Data format (*.mat)
**Low**	5 Hz	*cnt*_Low(1), *cnt*_Low(2)<br>*mrk*_Low(1), *mrk*_Low(2)
	5.5 Hz	
	6 Hz	
	6.5 Hz	
**Middle**	21 Hz	*cnt*_Middle(1), *cnt*_Middle(2)<br>*mrk*_Middle(1), *mrk*_Middle(2)
	21.5 Hz	
	22 Hz	
	22.5 Hz	
**High**	40 Hz	*cnt*_High(1), *cnt*_High(2)<br>*mrk*_High(1), *mrk*_High(2)
	40.5 Hz	
	41 Hz	
	41.5 Hz	

Questionnaires

We asked subjects to fill out two different questionnaires before and after the experiment. Table 2 presents two sets of questionnaires. Seven questions (from A1 to A7) and other three questions (from B1 to B3) were asked before the experiment to record the demographics and

initial physical condition of the subject and after the experiment to check physical conditions of the subject (e.g., drowsiness, concentration, and eye strain), respectively. The answers of the questionnaires are provided with a supplementary file (questionnaires_answers.xlsx). Note that because all subjects were 20s university students and they did not take any medication and drink alcohol 24 h before the experiment, we did not include the related information (A-2: Age Group, A-5: Drinking Alcohol, and A-7: Drug) in the supplementary file.

Table 2. Two sets of questionnaires performed before and after the experiment.

**Before Experiment**		
**NUMBER**	**QUESTIONNAIRE**	**ANSWER**
A-1	Gender	Male = 1/ Female = 2
A-2	Age Group	10s = 1/ 20s = 2/ 30s = 3/ More than 40s = 4
A-3	Job	Middle/High school student = 1/ Undergraduate = 2/ Postgraduate = 3/ The others = 4
A-4	Sleeping Hours	Less than 5 h = 1/ 6 h =2/ 7 h = 3/ 8 h = 4/ More than 9 h = 5
A-5	Drinking Alcohol	No = 1/ Yes = 2
A-6	Overall Body Condition	(Good) 1 2 3 4 5 6 7 8 9 10 (Bad)
A–7	Drug	No = 1/ Yes = 2
**After Experiment**		
**NUMBER**	**QUESTIONNAIRE**	**ANSWER**
B-1	Drowsiness	(Good) 1 2 3 4 5 6 7 8 9 10 (Bad)
B-2	Concentration	(Bad) 1 2 3 4 5 6 7 8 9 10 (Good)
B-3	Eye Strain	(Good) 1 2 3 4 5 6 7 8 9 10 (Bad)

Data Validation

Methods

Because our main concern is the EEG dataset measured during the SSVEP experiment, we provide detailed analysis results for the EEG dataset while example time series results for the other physiological dataset. The EEG data were first band-pass filtered with different cutoff frequencies according to the stimulation frequency band: 3 – 9 Hz, 18 – 24 Hz, and 38 – 44 Hz for low-, middle-, and high-frequency band, respectively. From the band-pass filtered data, we extracted 6 s epochs measured while the subjects were focusing on each target LED, and used them for further analysis. In order to visually observe SSVEP responses, spectral powers were estimated for each channel by applying a moving-window technique (2.5 s window size with 90 % overlap). Canonical correlation analysis (CCA) that has been the most widely used method for classification of SSVEP data was used for 4-class classification [30].

Results

Figure 3 shows SSVEP topographic maps averaged over two days with all subjects for the four stimulation frequencies of each frequency band. As expected, strong SSVEPs are observed around occipital areas for all cases. High spectral powers are also observed around fronto-temporal areas, which would be derived from electrooculography (EOG). As well known, absolute spectral powers decrease from the low frequency band to the high frequency band (see the color bar range in figure 3). Occipital SSVEPs are relatively high as compared to those of the other brain areas in the middle frequency band, showing spatially high SSVEP SNR.

[Figure 3 here]

Figure 4 shows grand-average spectral powers estimated using EEGs measured from 13 parieto-occipital channels (Ch_Set4) during visual stimulation for the four stimulation frequencies of three frequency bands. Spectral peaks are observed at stimulation frequencies regardless of frequency band. Note that among 60 sub-datasets (30 subjects x 2 days), 10 datasets were excluded for this analysis because these datasets show extremely large SSVEP amplitudes at non-stimulation frequencies for some trials and thereby distorting the grand-average results (Day 1 and 2 for S2; Day 2 for S10; Day1 and 2 for S11; Day2 for S13; Day1 for S18; Day1 for S20; Day1 and 2 for S29).

[Figure 4 here]

Changes in classification accuracy are presented for each stimulation frequency band in figure 5 with respect to channel configuration shown in figure 2. Classification accuracy gradually increases as the number of channels decreases from frontal areas until 8 channels (Ch_Set5) are employed for classification regardless of frequency band, meaning that occipital areas are most associated with visual information processing and thus provide most discriminative information. However, classification performance considerably drops when using only 3 channels (Ch_Set6: O1, O2, and Oz) attached on occipital areas due to fewer information.

[Figure 5 here]

Figure 6 shows the mean classification accuracies of each frequency band for two different days, which were obtained using the best channel configuration (Ch_Set5) in terms of classification accuracy shown in figure 5. A similar trend is shown for two different days from the statistical point of view; the mean classification accuracy of the middle frequency band is significantly higher than those of the low- and high-frequency bands, and the mean classification accuracy of the low frequency band does than that of the high frequency band only for the second day (RM-ANOVA: $F(2, 29) = 19.36$, $p < 0.01$; paired t-test Bonferroni corrected $p < 0.05$: middle > low = high for the first day; RM-ANOVA: $F(2, 29) = 22.88$, $p < 0.01$; paired t-test Bonferroni corrected $p < 0.05$: middle > low > high for the second day). No significant difference is observed between two days in terms of stimulation frequency band.

[Figure 6 here]

Examples of six physiological data measured with EEGs are presented in figure 7. Because physiological data show high inter- and intra-subject variability, representative examples are provided for each of six physiological data. The example data were acquired from S2 when the subject focused on an LED modulated at 5 Hz for 6 s during the first trial. In particular, two breathings and seven heartbeats are clearly observed for 6 s from respiration (figure 7(a)) and ECG data (figure 7(b)), respectively. Two EMG (figures 7(c) and (d)) and head motion (figure 7(e)) data show that no significant movement was made during the first trial, and heartbeats are also observed from two EMG data (figures 7(c) and (d)). Body temperature monotonically increases, but not significant (figure 7(f)).

[Figure 7 here]

Re-use potential

Although SSVEP is one of the most widely used BCI paradigms [31], publicly available SSVEP BCI datasets have been rarely provided to date. In this study, we provided a multi-band and multi-day SSVEP BCI dataset for the first time, and validated its feasibility based on SSVEP spectral power and classification analysis. All results were coincide with those reported in previous studies; SSVEP responses are mainly observed around occipital areas with spectral peaks at stimulation frequencies regardless of stimulation frequency band, and the classification accuracy of the middle frequency band was higher than those of the low- and high-frequency band [25, 32]. Our multi-band SSVEP dataset can be used to investigate subject-specific stimulation frequencies by comparing characteristics of SSVEPs evoked in each of the three frequency bands, thereby improving the performance of SSVEP-based BCIs. Also, the multi-day SSVEP dataset can be used to develop advanced solutions for session-to-session (day-to-day) transfer issues by investigating changes in SSVEP characteristics over different days, thereby enhancing the reliabity of SSVEP-based BCIs.

All other physiological data simultaneously measured with the EEG data also showed reasonable results even though example results were only shown due to high inter- and intra-subject variability. The physiological data can be used not only for investigating the relation between changes in brain activity and physiological condition, but also for developing artifact correction methods for SSVEP. For the latter case, IMU and EMG data can be used in particular to detect head/neck movements that would worsen the quality of EEG data, and then to correct them based on advanced algorithms.

Availability requirements

It will be filled out later after an initial editorial assessment for scope and scale.

Availability of supporting data

The data supporting this paper, including EEG and other physiological datasets and questionnaire results, are available in the GigaScience database, GigaDB.

Declarations

List of abbreviations

SSVEP: steady-state visual evoked potential; BCI: brain-computer interface; ERP: event-related potential; SNR: signal-to-noise ratio; CCA: canonical correlation analysis; electromyography: EMG; electrocardiography: ECG; inertial measurement unit: IMU; electrooculography: EOG.

Ethical approval

This study was approved by the Institutional Review Board (IRB) of Kumoh National Institute of Technology (No.6250).

Competing interests

The authors declare that they have no competing interests.

Authors' contributions

G.-Y.C., and H.-J.H., designed the experiment, Y.-J.J., implemented an SSVEP stimulator, G.-Y.C., acquired the data, and G.-Y.C., C.-H.H., Y.-J.J., performed data analysis, and H.-J.H., supervised this study. All authors wrote and reviewed the manuscript.

Acknowledgements

This work was supported by the Institute for Information & Communications Technology Planning & Evaluation (IITP) grant funded by the Korea government (No. 2017-0-00451; Development of BCI based Brain and Cognitive Computing Technology for Recognizing User's Intentions using Deep Learning).

References

1. Pfurtscheller G, Flotzinger D and Kalcher J. Brain-computer interface-a new communication device for handicapped persons. J Microcinoyt Appl. 1993;16(3):293-9.

2. Wolpaw JR, Birbaumer N, McFarland DJ, Pfurtscheller G and Vaughan TM. Brain-computer interfaces for communication and control. Clin Neurophysiol. 2002;113(6):767-91.

3. Nicolas-Alonso LF and Gomez-Gil J. Brain computer interfaces, a review. Sensors. 2012;12(2):1211-79.

4. Decety J and Ingvar DH. Brain structures participating in mental simulation of motor behavior: A neuropsychological interpretation. Acta psychol. 1990;73(1):13-34.

5. Jeannerod M and Frak V. Mental imaging of motor activity in humans. Curr Opin Neurobiol. 1999;9(6):735-9.

6. Pfurtscheller G, Brunner C, Schlögl A and Da Silva FL. Mu rhythm (de) synchronization and EEG single-trial classification of different motor imagery tasks. NeuroImage. 2006;31(1):153-9.

7. Pfurtscheller G and Neuper C. Motor imagery and direct brain-computer communication. Proc IEEE. 2001;89(7):1123-34.

8. Blankertz B, Müller K-R, Curio G, Vaughan TM, Schalk G, Wolpaw JR, et al. The BCI competition 2003: Progress and perspectives in detection and discrimination of EEG single trials. IEEE Trans Biomed Eng. 2004;51(6):1044-51.

9. Blankertz B, Müller K-R, Krusienski DJ, Schalk G, Wolpaw JR, Schlogl A, et al. The BCI competition III: Validating alternative approaches to actual BCI problems. IEEE Trans Neural Syst Rehabil Eng. 2006;14(2):153-9.

10. Cho H, Ahn M, Ahn S, Kwon M and Jun SC. EEG datasets for motor imagery brain computer interface. Gigascience. 2017:6(7):gix034.

11. Sajda P, Gerson A, Müller K-R, Blankertz B and Parra L. A data analysis competition to evaluate machine learning algorithms for use in brain-computer interfaces. IEEE Trans Neural Syst Rehabil Eng. 2003;11(2):184-5.

12. Tangermann M, Müller K-R, Aertsen A, Birbaumer N, Braun C, Brunner C, et al. Review of the BCI competition IV. Front Neurosci. 2012;6:55.

13. Shin J, von Luhmann A, Blankertz B, Kim D-W, Jeong J, Hwang H-J, and Müller K-R.

Open access dataset for EEG+ NIRS single-trial classification. IEEE Trans Neural Syst Rehabil Eng. 2017;25(10):1735-45.

14. BNCI Horizon 2020 Datasets;. Accessed: 2019-03-25. http://bnci-horizon-2020.eu/database/data-sets.

15. Farwell LA and Donchin E. Talking off the top of your head: toward a mental prosthesis utilizing event-related brain potentials. Elecroen Clin Neuro. 1988;70(6):510-23.

16. Sakurada T, Kawase T, Takano K, Komatsu T and Kansaku K. A BMI-based occupational therapy assist suit: Aasynchronous control by SSVEP. Front Neurosci. 2013;7:172.

17. Kwak N-S, Müller K-R and Lee S-W. A lower limb exoskeleton control system based on steady state visual evoked potentials. J Neural Eng. 2015;12(5):056009.

18. Gollee H, Volosyak I, McLachlan AJ, Hunt KJ and Gräser A. An SSVEP-based brain-computer interface for the control of functional electrical stimulation. IEEE Trans Biomed Eng. 2010;57(8):1847-55.

19. Hwang H-J, Lim J-H, Jung Y-J, Choi H, Lee SW and Im C-H. Development of an SSVEP-based BCI spelling system adopting a QWERTY-style LED keyboard. J Neurosci Methods. 2012;208(1):59-65.

20. Lim J-H, Lee J-H, Hwang H-J, Kim DH and Im C-H. Development of a hybrid mental spelling system combining SSVEP-based brain-computer interface and webcam-based eye tracking. Biomed Signal Process Control. 2015;21:99-104.

21. Wang Y, Chen X, Gao X and Gao S. A benchmark dataset for SSVEP-based brain-computer interfaces. IEEE Trans Neural Syst Rehabil Eng. 2017;25(10):1746-52.

22. Lee M-H, Kwon O, Kim Y-J, Kim H-K, Lee Y-E, Williamson J, et al. EEG Dataset and OpenBMI Toolbox for Three BCI Paradigms: An Investigation into BCI Illiteracy. GigaScience. 2019:giz002.

23. Herrmann CS. Human EEG responses to 1-100 Hz flicker: resonance phenomena in visual cortex and their potential correlation to cognitive phenomena. Exp Brain Res. 2001;137(3-4):346-53.

24. Galloway N. Human brain electrophysiology: Evoked potentials and evoked magnetic fields in science and medicine. Br J Ophthalmol. 1990;74(4):255.

25. Volosyak I, Valbuena D, Luth T, Malechka T and Graser A. BCI demographics II: How many (and what kinds of) people can use a high-frequency SSVEP BCI? IEEE Trans Neural

Syst Rehabil Eng. 2011;19(3):232-9.

26. Sakurada T, Kawase T, Komatsu T and Kansaku K. Use of high-frequency visual stimuli above the critical flicker frequency in a SSVEP-based BMI. Clin Neurophysiol. 2015;126(10):1972-8.

27. Cho H, Ahn M, Kim K and Jun SC. Increasing session-to-session transfer in a brain-computer interface with on-site background noise acquisition. J Neural Eng. 2015;12(6):066009

28. Krauledat M, Tangermann M, Blankertz B and Müller K-R. Towards zero training for brain-computer interfacing. PloS one. 2008;3(8):e2967.

29. Samek W, Meinecke FC and Müller K-R. Transferring subspaces between subjects in brain-computer interfacing. IEEE Trans Biomed Eng. 2013;60(8):2289-98.

30. Lin Z, Zhang C, Wu W and Gao X. Frequency recognition based on canonical correlation analysis for SSVEP-based BCIs. IEEE Trans Biomed Eng. 2006;53(12):2610-4.

31. Hwang H-J, Kim S, Choi S, Im C-H. EEG-based brain-computer interfaces: A thorough literature survey. Int J Hum-Comput Interact. 2013;29(12): 814-826.

32. Müller SMT, Diez PF, Bastos-Filho TF, Sarcinelli-Filho M, Mut V, Laciar E, and Avila E. Robotic wheelchair commanded by people with disabilities using low/high-frequency ssvep-based BCI. In World Congress on Medical Physics and Biomedical Engineering, 2015. pp. 1177-1180.

Figures

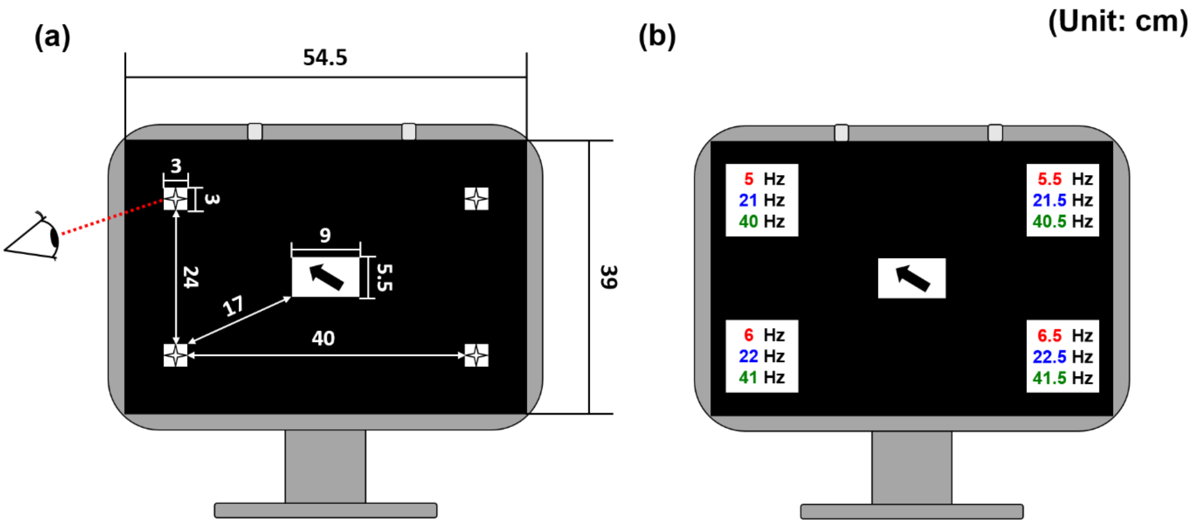

Figure 1. (a) Schematic diagram of the SSVEP stimulator (unit: cm). (b) Placement of four stimulation frequencies for each of three stimulation frequency bands (5, 5.5, 6, and 6.5 Hz for the low frequency band; 21, 21.5, 22, 22.5 Hz for the middle frequency band; 40, 40.5, 41, and 41.5 Hz for the high frequency band).

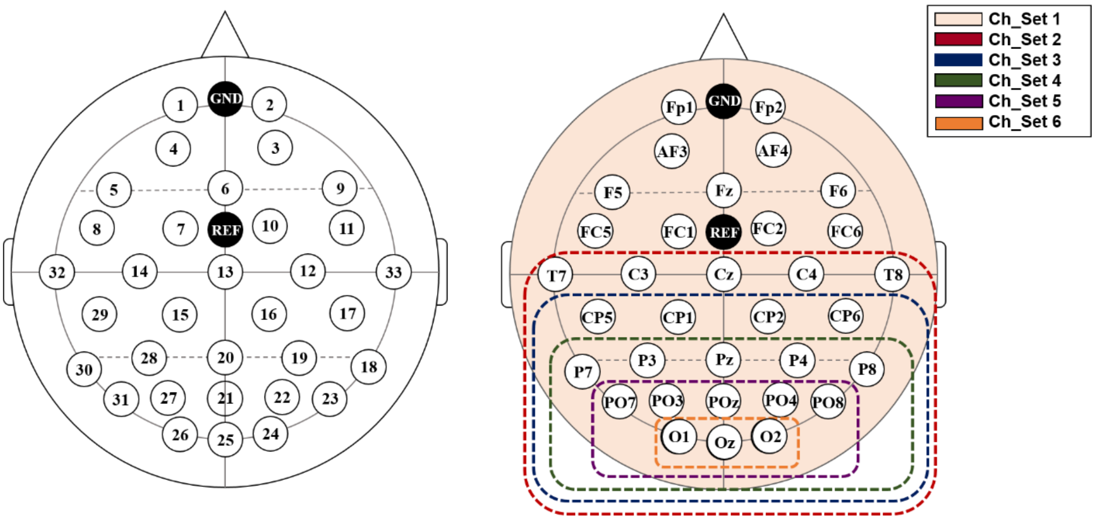

Figure 2. Electrode position used in the experiment with respect to (a) number and (b) position name. Note that six different channel sets are used for data analysis to see the impact of the number of electrodes on classification performance.

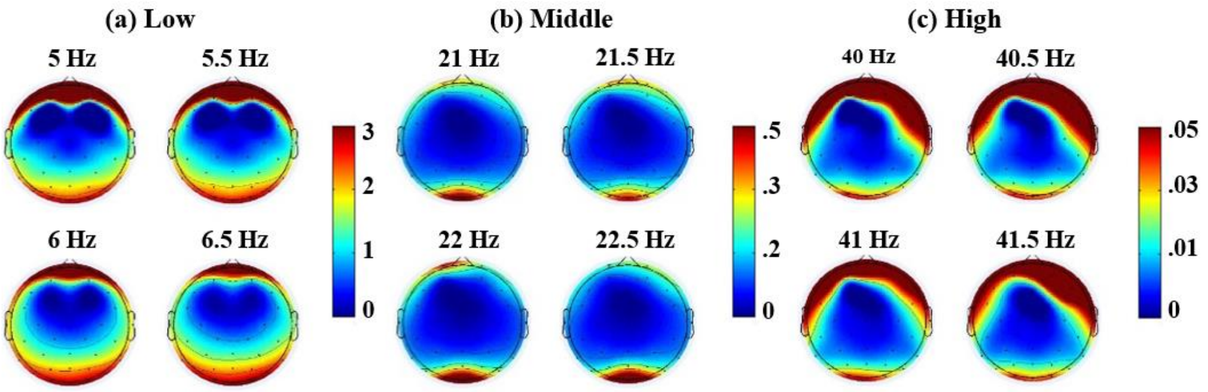

Figure 3. SSVEP topographic maps averaged over two days with all subjects for the four stimulation frequencies of (a) the low, (b) middle, and (c) high frequency band.

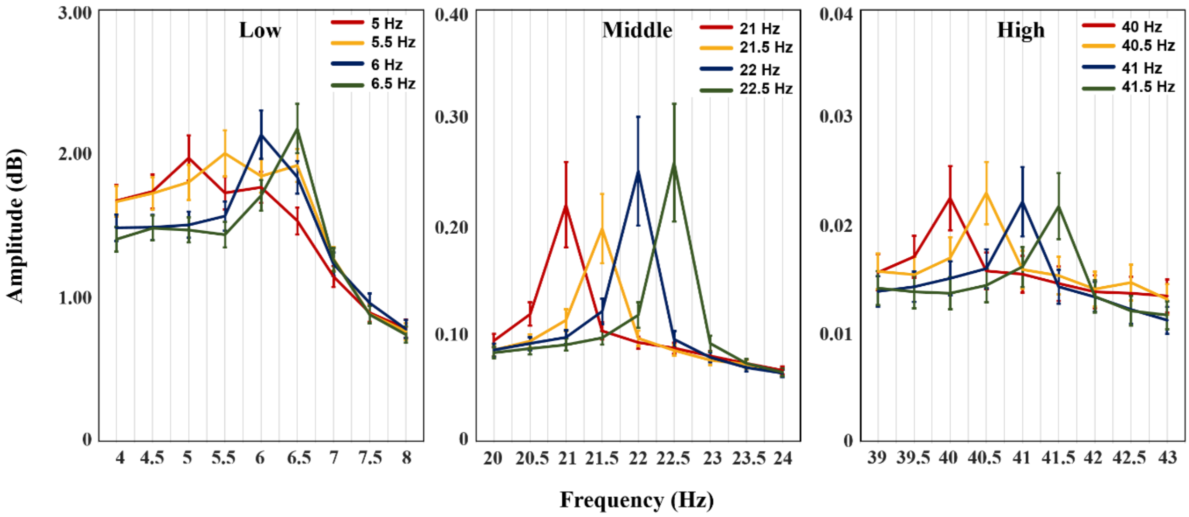

Figure 4. Grand-average SSVEP responses estimated using 13 parieto-occipital channels (Ch_Set 4) for each frequency band. Spectral peaks are observed at each of stimulation frequencies. The vertical bars indicate the standard errors of spectral powers for each frequency.

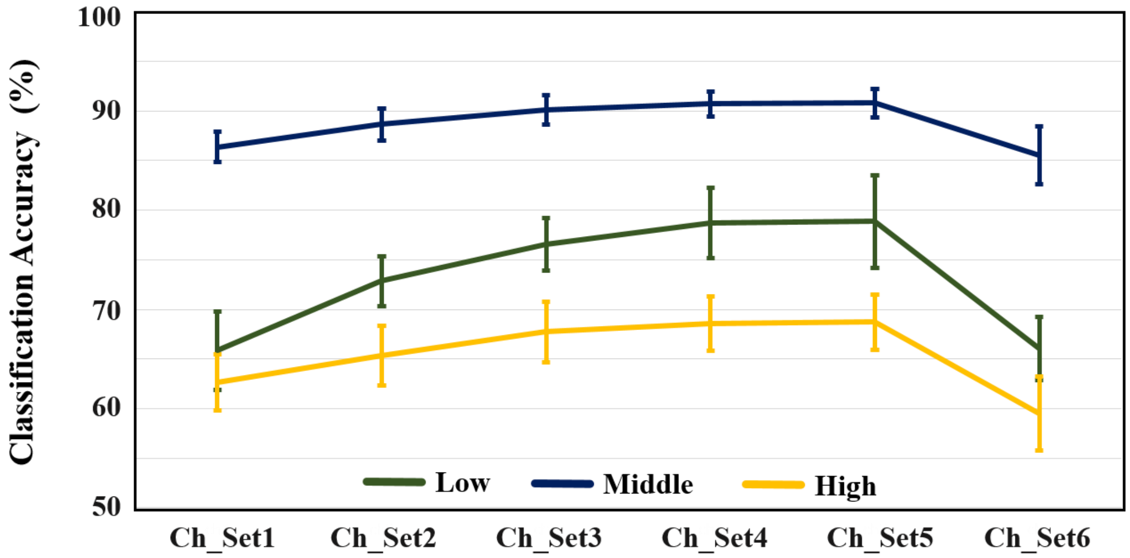

Figure 5. Changes in classification accuracy in terms of channel configuration for each stimulation frequency band. Eight channels attached on occipital areas (Ch_Set5) show the highest mean classification accuracy for all of the three frequency bands. The vertical bars indicate the standard deviations of classification accuracies for each channel set.

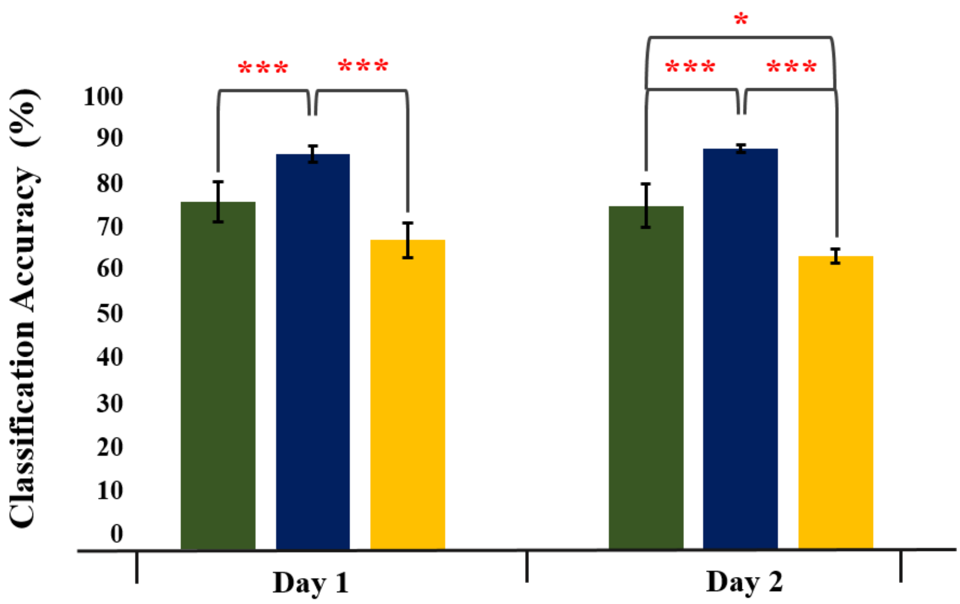

	Day 1	Day 2
Low	0.79 (± 0.05)	0.78 (± 0.05)
Middle	0.90 (± 0.02)	0.91 (± 0.01)
High	0.70 (± 0.04)	0.67 (± 0.02)

Figure 6. Mean classification accuracies of three frequency bands for two different days (RM-ANOVA: $F(2, 29) = 19.36$, $p < 0.01$; paired t-test Bonferroni corrected $p < 0.05$: middle > low = high for the first day; RM-ANOVA: $F(2, 29) = 22.88$, $p < 0.01$; paired t-test Bonferroni corrected $p < 0.05$: middle > low > high for the second day). The vertical bars indicate the standard deviations of classification accuracies for each frequency band.

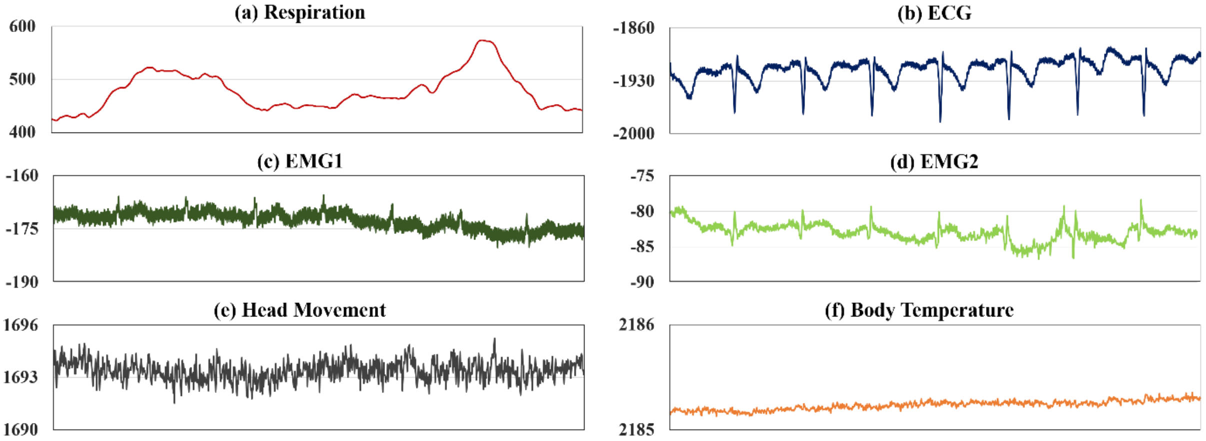

Figure 7. Examples of six physiological data with vendor-specific units: (a) respiration, (b) ECG, (c) EMG1 (left side of the back of the neck), (d) EMG2 (right side of the back of the neck), (e) head movement, and (f) body temperature, respectively.

Click here to access/download
Supplementary Material
questionnaires_answers.xlsx
