# Supplementary material for: A multi-day and multi-band dataset for a steady-state visual-evoked potential–based brain-computer interface
Source: Gigascience. 2019 Nov 25;8(11):giz133. doi: 10.1093/gigascience/giz133 (PMC6876666; doi:10.1093/gigascience/giz133)
Supplement: giz133_GIGA-D-19-00116_Revision_1 [file giz133_giga-d-19-00116_revision_1.pdf]

GigaScience

A multi-day and multi-band dataset for steady-state visual evoked potential-based brain-computer interface

--Manuscript Draft--

**Manuscript Number:**	GIGA-D-19-00116R1
**Full Title:**	A multi-day and multi-band dataset for steady-state visual evoked potential-based brain-computer interface
**Article Type:**	Data Note
**Funding Information:**	Institute for Information and Information & Communications Technology Planning & Evaluation (2017-0-00451) — Prof. Han-Jeong Hwang
**Abstract:**	Background Steady-state visual evoked potential (SSVEP) is a brain response to a visual stimulus modulated at a certain frequency, and it has been widely used in electroencephalography (EEG)-based brain-computer interface (BCI) research. However, accessible SSVEP datasets for BCI have been rarely published. In this study, we provide a new SSVEP dataset measured from thirty subjects for two different days, which complements existing SSVEP datasets for the three following aspects: i) multi-band SSVEP datasets are provided by using all possible three frequency bands (low, middle, and high) used for SSVEP stimulation, ii) multi-day datasets are provided, and iii) EEG datasets are provided along with physiological data, such as respiration, electrocardiography, electromyography, head motion and body temperature. Findings To validate our dataset, we estimated spectral powers and classification performance for the EEG (SSVEP) datasets, and showed example time-series data for physiological data. Strong SSVEP responses were observed at stimulation frequencies, and the mean classification performance of the middle frequency band was significantly higher than that of the low- and high-frequency bands. Other physiolgical data also showed reasonable results. Conclusions Our multi-band and multi-day SSVEP datasets can be used to optimize stimulation frequencies by simultaneously investigating the characteristics of SSVEPs evoked in each of the three frequency bands and solve session-to-session (day-to-day) transfer issues by investigating non-stationarity of SSVEPs measured from different days, respectivley. Also, auxiliary physiological data can be used to explore the relation between SSVEP characteristics and physiological conditions, thereby providing useful information in optimizing experimental paradigms to attain high performance.
**Corresponding Author:**	Han-Jeong Hwang<br>Kumoh National Institute of Technology<br>Gumi, Gyeongsangbuk-do KOREA, REPUBLIC OF
**Corresponding Author Secondary Information:**	
**Corresponding Author's Institution:**	Kumoh National Institute of Technology
**Corresponding Author's Secondary Institution:**	
**First Author:**	Ga-Young Choi
**First Author Secondary Information:**	
**Order of Authors:**	Ga-Young Choi<br>Chang-Hee Han<br>Young-Jin Jung<br>Han-Jeong Hwang
**Order of Authors Secondary Information:**	
**Response to Reviewers:**	We responded to all comments the reviewers made in the Personal Cover file.

Powered by Editorial Manager® and ProduXion Manager® from Aries Systems Corporation

Additional Information:	
Question	Response
Are you submitting this manuscript to a special series or article collection?	No
**Experimental design and statistics**<br><br>Full details of the experimental design and statistical methods used should be given in the Methods section, as detailed in our Minimum Standards Reporting Checklist. Information essential to interpreting the data presented should be made available in the figure legends.<br><br>Have you included all the information requested in your manuscript?	Yes
**Resources**<br><br>A description of all resources used, including antibodies, cell lines, animals and software tools, with enough information to allow them to be uniquely identified, should be included in the Methods section. Authors are strongly encouraged to cite Research Resource Identifiers (RRIDs) for antibodies, model organisms and tools, where possible.<br><br>Have you included the information requested as detailed in our Minimum Standards Reporting Checklist?	Yes
**Availability of data and materials**<br><br>All datasets and code on which the conclusions of the paper rely must be either included in your submission or deposited in publicly available repositories (where available and ethically appropriate), referencing such data using a unique identifier in the references and in the “Availability of Data and Materials” section of your manuscript.	No

Have you have met the above requirement as detailed in our Minimum Standards Reporting Checklist?	
If not, please give reasons for any omissions below.<br><br>as follow-up to "**Availability of data and materials**<br><br>All datasets and code on which the conclusions of the paper rely must be either included in your submission or deposited in publicly available repositories (where available and ethically appropriate), referencing such data using a unique identifier in the references and in the "Availability of Data and Materials" section of your manuscript.<br><br>Have you have met the above requirement as detailed in our Minimum Standards Reporting Checklist?<br><br>"	As far as I know, we can make our data open to reviewers after an initial check. Thus, I will provide our data after an initial check.

Manuscript

Click here to access/download;Manuscript;GigaSicence_RevisedManuscript

Click here to view linked References

A multi-day and multi-band dataset for steady-state visual evoked potential based Brain-Computer Interface

Ga-Young Choi[1], Chang-Hee Han[2], Young-Jin Jung[3], Han-Jeong Hwang[1,*]

E-mail: cgy326@naver.com, zeros8706@naver.com, microbme@outlook.com, h2j@ kumoh.ac.kr

[1]Department of Medical IT Convergence Engineering, Kumoh National Institute of Technology, Gumi 39177, Republic of Korea

[2]Machine Learning Group, Berlin Institute of Technology (TU Berlin), 10623 Berlin, Germany

[3]Department of Radiological Science, Dongseo University, Busan 47011, Republic of Korea

Number of Pages: ~~22~~30

Number of Figures: ~~7~~8

Number of Tables: 2

Corresponding Author Information:

Name: Han-Jeong Hwang

Address: Kumoh National Institute of Technology, 350-27, Gumi-si, Gyeongsangbuk-do, Republic of Korea

Tel.: +82-054-478-7783

E-mail: h2j@kumoh.ac.kr

Abstract

Background: Steady-state visual evoked potential (SSVEP) is a brain response to a visual stimulus modulated at a certain frequency, and it has been widely used in electroencephaography (EEG)-based brain-computer interface (BCI) research. However, accessable SSVEP datasets for BCI have been rarely published. In this study, we provide a new SSVEP dataset measured from thirty subjects for two different days, which complements existing SSVEP datasets for the three following aspects: i) multi-band SSVEP datasets are provided by using all possible three frequency bands (low, middle, and high) used for SSVEP stimluation, ii) multi-day datasets are provided, and iii) EEG datasets are provided along with physiolgical data, such as respiration, electrocardiography, electromyography, head motion (accelerator), and body temperature.

Findings: To validate our dataset, we estimated spectral powers and classification peformance for the EEG (SSVEP) datasets, and showed example time-series data for physiological data. Strong SSVEP responses were observed at stimulation frequencies, and the mean classification performance of the middle frequency band was significantly higher than the low- and high-frequency bands. Other physiological data also showed ~~reasonalbe~~ reasonable results.

Conclusions: Our multi-band and multi-day SSVEP datasets can be used to optimize stimulation frequencies by simultaneously investigating the characteristics of SSVEPs evoked in each of the three frequency bands and solve session-to-session (day-to-day) transfer issues by investigating non-stationarity of SSVEPs measured from different days, respectivley. Also, auxiliary physiological data can be used to explore the relation between SSVEP characteristics and physiological conditions, thereby providing useful information in optimizing experimental paradigms to attain high performance.

Key words: steady-state visual evoked potential (SSVEP); brain-computer interface (BCI); electroenchephalography (EEG); physiologcal data

Data Description

Background and purpose

Brain-computer interface (BCI) is a non-muscular communication method using brain activity, such as electroencephalograhy (EEG), for handicapped individuals who are unable to voluntarily control their bodies [1, 2]. Two approaches have been employed to develop EEG-based BCIs whether external stimuli are used or not [3]; an endogenous BCI uses mental imagery tasks while an exogenous BCI uses external stimlui in order to induce and evoke certain brain patterns, respectivley.

A presentative endogenous BCI paradigm is motor imagery that is defined as mental simulation of motor behaviors, e.g., left/right hand movement [4, 5]. Thanks to event-related (de)synchroniztion (ERD/S) phenomenon, different motor imagery tasks can be discriminated using mahcnine learning techniques, and then it can be used for BCI purpose [6. 7]. So far, a large number of motor imagery BCI datasets have been published [8-14], and they have contributed to significant advancement in the BCI community. Other endogenous BCI datasets are also available, such as slow cortical potential (SCP), readiness potential [8], and mental arithmetic [13, 14].

There are two representative exogenouse BCI paradigms: event-realted potential (ERP) and steady-state visual evoked potential (SSVEP). ERP is a time-locked brain response evoked in response to specific visual, auditory, and tactile stimuli while SSVEP is a period brain response to a visual stimuli modulated at a certain frequency. ERP has been mostly used in developing row/column matrix spellers [15], while SSVEP has been used in developing a variety of BCI applications, such as control of a robotic arm [16], an exoskelton [17], and a functional

electrical stimulation (FES) [18], and spelling of a character [19, 20]. Many ERP BCI datasets have become publicly available since the first ERP BCI dataset was publised in 2003 [8]. However, it was not until 2017 that a freely accessible SSVEP BCI dataset was published for the first time [21], and it was followed by the second one in 2019 even though the SSVEP paradigm has been widely used for BCI purpose due to high performance with little training [22].

Due to the lack of SSVEP BCI datasets compared to those based on other BCI paradigms, it would be benificial for BCI researchers to provide a new SSVEP BCI dataset that can complement the existing SSVEP BCI datasets. The first SSVEP dataset was measured from 35 subjects with a 40-target BCI speller, where SSVEP sitmulation frequencies ranged from 8 to 15.8 Hz with a span of 0.2 Hz [21]. The second SSVEP dataset was acquired from 54 subjects with a 4-class BCI system over two sessions, where 5.45, 6.67, 8.57, and 12 Hz were used as stimluation frequencies [22].

In this study, we proivde a new SSVEP BCI dataset that can contribute to SSVEP-based BCI research in three main aspects. First, our SSVEP dataset consists of three sub-datasets that are measured with three different frequency bands, respectively: low (1 – 12 Hz), middle (12 – 30 Hz), and high (30 – 60 Hz) frequency band. It is well documented that SSVEP is elicited in a wide rage of frequencies, rainging from 1 to 90 Hz [23], and the frequencies can be divided into three sub-frequency bands as mentioned above (low, middle, and high) [24]. The two previous SSVEP datasts were acquired using stimulation frequencies in certain frequency bands, i.e., 8 – 15.8 Hz in the low and middle frequeny bands [21] and 5.45 – 12 Hz in the low frequency band [22]. Considering that choice of the stimulation frequency band is one of the important factors that significantly affect SSVEP-based BCI performance [25], the

characteristics of SSVEPs evoked in each of the three frequency bands should be simultaneously investigated in terms of their signal-to-noise ratio (SNR) and classification performance. In particular, the high frequency band has been recevied increasing attention as an alternative to the low and middle frequency bands due to less visual fatigue despite relatively low perfomance [26]. However, no SSVEP BCI studies have provided availalbe datasets for the high frequency band. Thus, it is necessray to provide an SSVEP dataset recorded using the high frequency band along with those recorded using the low and middle frequency bands to simultaneously investigate the mentioned issues. Our SSVEP dataset can make it possible because it was measured by using the three freuqency bands independently from same subjects. Secondly, we provide a multi-session (day) dataset that was recorded over two different days from same subjects. Thus, our SSVEP dataset can be used to study session-to-session transfer which is an challening issue in BCI research [27-29]. A multi-session SSVEP dataset was also provided in [22], but which was acquried on the same day with a short break (i.e., 3 min). Therefore, our dataset can be more usefully used to get profound insights into the non-stationary nature of EEG, thereby providing useful solutions to overcome session-to-session (day-to-day) transfer issues. Finally, we provide other physiological datasets, which were not presented in the two previous SSVEP datasets [22, 23], along with the EEG dataset to check changes in physiolgical conditions of subjects during the experiment, such as respiration, electrocardiography (ECG), neck electromyography (EMG), head motion and body temperature. The auxiliary physiological data can be used to explore the relationship between SSVEP characteristics (e.g., SNR) and physiological conditions, thereby providing useful information in designing experimental paradigms to attain high performance.

In order to provide a novel SSVEP BCI dataset complementary to the previous two SSVEP

BCI datasets, we designed a 4-class SSVEP paradigm as that used for acquring the second SSVEP BCI dataset [22]. Three sets of four stimluation frequencies were employed for the low, middle, and high frquency bands, respectively. The SSVEP BCI dataset was acquired from thiry subjects from two different days. For data validation, we applied a standard analysis method to our SSVEP dataset, and provide baseline results along with all of the mentioned physiological datasets in this study.

Experimental design

Subject

Thirty subjects (9 females and 21 males; 23.8 ± 1.3 years) were recruited for this study. The number of subjects was decided because a sample size of 30 is enough to apply parametric statistical tests for analysis results. Note that parametric statistical tests provide more statistical power than non-parametric ones, thereby ensuring more reliable validation of our SSVEP dataset. They had no history of psychiatric diseases that might affect research results. Seven of thirty subjects had prior BCI experience, but they participated in endogenous BCI experiments based on a mental arithmetic task. Thus, it is assumed that their prior BCI experience would not significantly affect research results. They were given the details of experimental procedures, and signed an informed consent for study participation and anonymously data open to the public before the experiment. Adequate reimbursement was provided for their participation after the experiment. This study was approved by the Institutional Review Board (IRB) of Kumoh National Institute of Technology (No.6250), and was conducted in accordance with the principles of the declaration of Helsinki.

Stimulator

The SSVEP stimulator was made of two square styrofoams, a black thick paper, an opaque film, four LEDs and an LED controller. We first cut out five parts of a styrofoam, four of which was 3 x 3 cm for LED display, and the other was 9 x 5.5 cm to show an instruction during the experiment (Figure 1(a)). After that, we inserted four LEDs into the four square wholes of 3 x 3 cm (part number: T03WC01; operating current: 20 mA; viewing angle: θ/2 = 100◦; luminous intensity: 2,000 mcd; emitting color: white), and attached another styrofoam to the back of the first styrofoam. The front part of the stimulator was covered with an opaque film to diffuse the light, and then we attached a black paper with five square wholes that were exactly matched to those in the front styrofoam on to the opaque film for better visibility. The stimulator was attached to a 21-inch LCD monitor, and an instruction, which LED the subject should focus on, was presented with an arrow from the monitor through the center square whole of 9 x 5.5 cm. A schematic diagram of the SSVEP stimulator is shown in figure 1(a). The distance between each LED and an instruction arrow presented on the center of the monitor was 17 cm. In order to control the stimulator, we used a LAUNCHXL-F28027 Board powered by C2000 MCU (Texas Instrument). A duty cycle was set at 50%, meaning that an LED has 50% on-time and 50% off-time.

As mentioned above, three different frequency bands (low: 1-12 Hz, middle: 12-30 Hz, and high: 30-60 Hz [30]) were independently used for SSVEP stimulation to get multi-band SSVEP datasets in this study. Three sets of four stimulation frequencies selected for each frequency band were as follows: 5, 5.5, 6, and 6.5 Hz for the low frequency band; 21, 21.5, 22, 22.5 Hz for the middle frequency band; 40, 40.5, 41, and 41.5 Hz for the high frequency band. The four stimulation frequencies for each frequency band were selected in such a way that the harmonic

frequencies of four frequencies in the low frequency band are not overlapped with four frequencies in each of the middle and high frequency bands, and the harmonic frequencies of four frequencies in the middle frequency band are not overlapped with four frequencies in the high frequency band. This was because simultaneous use of harmonic frequencies as different stimulation frequencies could significantly decrease the performance of SSVEP-based BCI systems [31]. Also, alpha frequency band was not considered because its use can produce a considerable number of false positives [30, 32] even though alpha band for SSVEP stimulation shows high signal-to-noise ratio. We assigned four stimulation frequencies to four LEDs, depending on the stimulation frequency band, as shown in figure 1(b).

[Figure 1 here]

Experimental paradigm

During the experiment, the subjects sat on a comfortable arm chair in front of the SSVEP stimulator attached on a 21-inch monitor away from about 1 m, and they were instructed to remain relaxed without any movements. Note that all instructions were presented on the center of the monitor and the subjects could perceive them through the center whole of the stimulator. For each trial, a blank screen was presented for 5 s, and then an arrow indicating one of the four LEDs was presented for 6 s, during which the subject was asked to gaze at a target LED according to the direction of an arrow. After that, a white cross mark ('+') was presented for 6 s as a short break for the next trial. A short beep sound was also presented for every transition of visual stimuli in order to lead to more explicit attention of the subjects. The direction of an arrow was randomly presented 20 times (20 trials) for each direction, resulting in a total of 80 trials, which was repeated for each frequency band (low, middle, and high), respectively. To

avoid excessive fatigue, a break was regularly given to each subject after acquiring every 40 trials (defined as one session) for at least 5 min, and also irregularly whenever the subjects wanted during the experiment. Each subject performed six sessions of an SSVEP experiment (2 sessions x 3 frequency bands), which was conducted twice for different days with an interval of at least one day. The three stimulation frequency bands were alternatively used for counterbalancing between subjects. In particular, all possible order combinations of the three frequency bands were as follows: (low-middle-high), (low-high-middle), (middle-low-high), (middle-high-low), (high-low-middle), (high-middle-low). Each order was randomly assigned to five subjects, respectively (6 orders x 5 subjects = 30 subjects), and a same order was used for two days once an order was assigned to each subject in the first day experiment. The experiment lasted about 2 h, including EEG preparation for each day.

Data recording

EEGs were measured using a BrainAmp EEG amplifier (Brain products, GmbH Ltd, Germany) with a sampling rate of 1,000 Hz, where the ground and reference electrodes were attached on Fpz and FCz, respectively (figure 2). We used thirty-three active electrodes mounted based on the international 10-10 system to measure EEGs (FP1, FP2, AF4, AF3, F5, Fz, FC1, FC5, F6, FC2, FC6, C4, Cz, C3, CP1, CP2, CP6, P8, P4, Pz, POz, PO4, PO8, O2, Oz, O1, PO3, P3, CP5, P7, PO7, T7, and T8), where electrodes were more densely mounted around occipital areas than the others because SSVEP is mainly originated from the occipital lobe. We did not control changes in electrode locations between two different days, but instead we tried to maintain the condition of EEG measurement between two different days for each subject. This was because to naturally make slight changes in electrode

locations, which would happen in daily BCI use, and thereby providing useful dataset to study session-to-session (day-to-day) transfer issues. Note that electrode location change between days is an important factor that cause EEG non-stationarity between different days [33].

[Figure 2 here]

We also measured various bio-signals simultaneously with the EEG data to check changes in physiological conditions, such as respiration, ECG, neck EMG, head motion, and body temperature. In order to measure these physiological data, we attached a respiratory belt to the chest, three ECG sensors on lead-I position (Einthoven's triangle), two EMG sensors on the right and left side of the neck, an inertial measurement unit (IMU) sensor on the top of the head between Cz and CPz, and a temperature sensor under the armpit, respectively. The same amplifier used for measuring EEG was used for recording the physiological data with a same sampling rate of 1,000 Hz, and thus all of the measured data were synchronized. The physiological data can be used for investigating the relation between changes in brain activity and physiological condition as well as for developing artifact correction algorithms. For example, some previous studies used EEG and ECG simultaneously to check mental status and stress level/metal effort [34, 35], and others used motion data to remove motion-related artifacts form EEG [36, 37].

Data format and structure

Because data analysis was performed using Matlab R2013b (MathWorks, Natick, MA, USA), we provide our dataset in the form of Matlab files (.mat). Each data folder named as subject

initial (e.g., S1) has two sub-folders for two sub-datasets measured from two different days (i.e., day1 and day2). Each sub-folder has *cnt* and *mrk* files, which contain continuous time series data for all physiological measurement (*cnt*) and the trigger information of the corresponding data (*mrk*), respectively. The *cnt* and *mrk* files have suffixes corresponding to three frequency bands and session numbers. For example, *cnt*_Low(1) means time series data measured using the low frequency band for SSVEP stimulation in the first session. Thus, the sub-folder of each subject contains the following six pairs of *cnt* and *mrk* files: *cnt*_Low(1), *mrk*_Low(1), *cnt*_Low(2), *mrk*_Low(2), *cnt*_Middle(1), *mrk*_Middle(1), *cnt*_Middle(2), *mrk*_Middle(2), *cnt*_High(1), *mrk*_High(1), *cnt*_High(2), and *mrk*_High (2). All data were down-sampled to 200 Hz when converting the raw data into Matlab compatible files. Table 1 shows all data files provided for each sub-folder.

Table 1. Data format. Each data folder of all subjects has two sub-folders for two sub-datasets measured from two different days, and each sub-folder has six pairs of *cnt* and *mrk* files (shown below) according to stimulation frequency band and session number.

Frequency Band	Stimulation Frequencies	Data format (*.mat)
**Low**	5 Hz	*cnt*_Low(1), *cnt*_Low(2)<br>*mrk*_Low(1), *mrk*_Low(2)
	5.5 Hz	
	6 Hz	
	6.5 Hz	
**Middle**	21 Hz	*cnt*_Middle(1), *cnt*_Middle(2)<br>*mrk*_Middle(1), *mrk*_Middle(2)
	21.5 Hz	
	22 Hz	
	22.5 Hz	
**High**	40 Hz	*cnt*_High(1), *cnt*_High(2)<br>*mrk*_High(1), *mrk*_High(2)
	40.5 Hz	
	41 Hz	
	41.5 Hz	

Questionnaire

We asked subjects to fill out two different questionnaires before and after the experiment. Table 2 presents two sets of questionnaires. Seven questions (from A1 to A7) and other three questions (from B1 to B3) were asked before the experiment to record the demographics and initial physical condition of the subject and after the experiment to check physical conditions of the subject (e.g., drowsiness, concentration, and eye strain), respectively. The answers of the questionnaires are provided with a supplementary file (questionnaires_answers.xlsx). Note that because all subjects were 20s university students and they did not task any medication and drink alcohol 24 h before the experiment, we did not include the related information (A-2: Age Group, A-5: Drinking Alcohol, and A-7: Medicine~~Drug~~) in the supplementary file.

Table 2. Two sets of questionnaires performed before and after the experiment.

**Before Experiment**		
**NUMBER**	**QUESTIONNAIRE**	**ANSWER**
A–1	Gender	Male = 1/ Female = 2
A–2	Age Group	10's = 1/ 20's = 2/ 30's = 3/ More than 40's = 4
A–3	Job	Middle/high school student = 1/ Undergraduate = 2/ Postgraduate = 3/ The others = 4
A–4	Sleeping Hours	Less than 5 h = 1/ 6 h =2/ 7 h = 3/ 8 h = 4/ More than 9 h = 5
A–5	Drinking Alcohol	No = 1/ Yes = 2
A–6	Overall Body Condition	(Good) 1 2 3 4 5 6 7 8 9 10 (Bad)
A–7	Medicine~~Drug~~	No = 1/ Yes = 2
**After Experiment**		
**NUMBER**	**QUESTIONNAIRE**	**ANSWER**

B - 1	Drowsiness	(Good) 1 2 3 4 5 6 7 8 9 10 (Bad)
B – 2	Concentration	(Bad) 1 2 3 4 5 6 7 8 9 10 (Good)
B - 3	Eye Strain	(Good) 1 2 3 4 5 6 7 8 9 10 (Bad)

Data Validation

Methods

Because our main concern is the EEG dataset measured during the SSVEP experiment, we provide detailed analysis results for the EEG dataset while example time series data for the other physiological datasets.

The EEG data were first band-pass filtered with different cutoff frequencies according to the stimulation frequency band: 3 – 9 Hz, 18 - 24 Hz, and 38 – 44 Hz for low-, middle-, and high-frequency band, respectively. From the band-pass filtered data, we extracted 6 s epochs measured while the subjects were focusing on each target LED, and used them for further analysis. In order to visually observe SSVEP responses, spectral powers were estimated for each channel by applying a moving-window technique (2.5 s window size with 90 % overlap). The SSVEP SNR was also calculated by dividing the mean spectral amplitude of 6 adjacent frequencies from the SSVEP amplitude at the stimulation frequency to demonstrate the reliability of our SSVEP dataset [38]. Canonical correlation analysis (CCA) that has been the most widely used method for classification of SSVEP data was used for 4-class classification [3~~0~~9].

Each of six physiological data was linearly detrended to remove baseline drift. Respiration rate and heart rate were estimated from respiration and ECG data, respectively, based on peak information for each frequency band and each session, to check the ranges of respiration and heart rates. The mean and standard deviation values were estimated for each trial for the other

physiological data (EMG1, EMG2, IMU, and body temperature) to see changes in each of the four physiological data.

Results

Figure 3 shows ~~SSVEP~~ topographic maps at the SSVEP frequencies averaged over two days with all subjects for the four stimulation frequencies of each frequency band. As expected, strong SSVEPs are observed around occipital areas for all cases. High spectral powers are also observed around fronto-temporal areas, which would be derived from electrooculography (EOG). As well known, absolute spectral powers decrease from the low frequency band to the high frequency band (see the color bar range in figure 3). Occipital SSVEPs are relatively high as compared to those of the other brain areas in the middle frequency band, showing spatially high SSVEP SNR.

[Figure 3 here]

Figure 4 shows SSVEP SNR topographic maps averaged over two days with all subjects for the four stimulation frequencies of each frequency band. Most channels show SSVEP SNRs higher than 1 for all stimulation frequencies, and in particular parieto-occipital channels show high SSVEP SNRs more than 2, demonstrating the reliability of our SSVEP datasets. All SSVEP SNRs are provided with 12 supplementary files (4 stimulation frequencies x 3 frequency bands) for each day, and each supplementary file contains SSVEP SNRs of each channel and each trial for all subjects.

[Figure 4 here]

Figure ~~4~~ 5 shows grand-average spectral powers estimated using EEGs measured from 13 parieto-occipital channels (Ch_Set4) during visual stimulation for the four stimulation frequencies of three frequency bands. Spectral peaks are observed at stimulation frequencies regardless of frequency band. Note that among 60 sub-datasets (30 subjects x 2 days), 10 datasets are excluded for this analysis because these datasets show extremely large SSVEP amplitudes at non-stimulation frequencies for some trials and thereby distorting the grand-average results (Day 1 and 2 for S2; Day 2 for S10; Day1 and 2 for S11; Day2 for S13; Day1 for S18; Day1 for S20; Day1 and 2 for S29).

[Figure ~~4~~ 5 here]

Changes in classification accuracy are presented for each stimulation frequency band in figure ~~5~~ 6 with respect to channel configuration shown in figure 2. Classification accuracy gradually increases as the number of channels decreases from frontal areas until 8 channels (Ch_Set5) are employed for classification regardless of frequency band, meaning that occipital areas are most associated with visual information processing and thus provide most discriminative information. However, classification performance considerably drops when using only 3 channels (Ch_Set6: O1, O2, and Oz) attached on occipital areas due to fewer information.

[Figure 56 here]

Figure ~~6~~ 7 shows the mean classification accuracies of each frequency band for two different days, which were obtained using the best channel configuration (Ch_Set5) in terms of classification accuracy shown in figure 56. A similar trend is shown for two different days from

the statistical point of view; the mean classification accuracy of the middle frequency band is significantly higher than those of the low- and high-frequency bands, and the mean classification accuracy of the low frequency band does than that of the high frequency band only for the second day (RM-ANOVA: $F(2, 29) = 19.87$, $p < 0.01$; paired t-test Bonferroni corrected $p < 0.05$: middle > low = high for the first day; RM-ANOVA: $F(2, 29) = 23.09$, $p < 0.01$; paired t-test Bonferroni corrected $p < 0.05$: middle > low > high for the second day). No significant difference is observed between two days in terms of stimulation frequency band.

[Figure ~~6~~ 7 here]

Example of six physiological data measured with EEGs are presented in figure ~~7~~8. Because physiological data show high inter- and intra-subject variability, representative examples are provided for each of six physiological data, and detailed results are provided with 6 supplementary figures and 12 supplementary files. The example data were measured from S2 when the subject started to focus~~ed~~ on an LED modulated at 5 Hz ~~for 6 s during~~ from the first trial for 60 s. In particular, ~~two~~ 13 breathings and ~~seven~~ 93 heartbeats are clearly observed for ~~6~~ 60 s from respiration (figure ~~7~~8(a)) and ECG data (figure ~~7~~8(b)), respectively, which fall into the normal ranges of adult respiration rate (12 – 18) [40] and heart rate (60 – 100) [41]. Two EMG (figures ~~7~~8(c) and (d)) and head motion (figure ~~7~~8(e)) data show that no significant movement was made ~~during the first trial~~, and heartbeats are also observed from two EMG data (figures ~~7~~8(c) and (d)). Body temperature also shows little changes ~~monotonically increases, but not significant~~ (figure ~~7~~8(f)). Most subjects showed similar trends for each of physiological data, except few cases (see supplementary figures and files).

[Figure ~~7~~ 8 here]

Re-use potential

Although SSVEP is one of the most widely used BCI paradigms [~~31~~42], publicly available SSVEP BCI datasets have been rarely provided to date. In this study, we provided multi-band and multi-day SSVEP BCI datasets for the first time, and validated their feasibility based on SSVEP spectral power and classification analysis. All results were coincide with those reported in previous studies; SSVEP responses are mainly observed around occipital areas with spectral peaks at stimulation frequencies regardless of stimulation frequency band, and the classification accuracy of the middle frequency band was higher than those of the low- and high-frequency band [25, ~~32~~43]. Our multi-band SSVEP datasets can be used to investigate subject-specific stimulation frequencies by comparing characteristics of SSVEPs evoked in each of the three frequency bands, thereby improving the performance of SSVEP-based BCIs. Also, the multi-day SSVEP datasets can be used to develop advnaced solutions for session-to-session (day-to-day) transfer issues by investigating changes in SSVEP characteristics over different days, thereby enhancing the reliabity of SSVEP-based BCIs.

All other physiological data simultaneously measured with the EEG data also showed reasonable results even though example results were only shown due to high inter- and intra-subject variability. The physiological data can be used not only for investigating the relation between changes in brain activity and physiological condition, but also for developing artifact correction methods for SSVEP. For the latter case, IMU and EMG data can be used in particular to detect head/neck movements that would worsen the quality of EEG data, and then to correct them based on advanced algorithms.

Availability requirements

It will be filled out later after an initial editorial assessment for scope and scale.

Availability of supporting data

The data supporting this paper, including EEG and other physiological datasets and questionnaire results, are available in the GigaScience database, GigaDB.

Declarations

List of Abbreviations

SSVEP: steady-state visual evoked potential; BCI: brain-computer interface; ERP: event-related potential; SNR: signal-to-noise ratio; CCA: canonical correlation analysis; electromyography: EMG; electrocardiography: ECG; inertial measurement unit: IMU; electrooculography: EOG.

Ethical Approval

This study was approved by the Institutional Review Board (IRB) of Kumoh National Institute of Technology (No.6250).

Competing interests

The authors declare that they have no competing interests.

Authors' contributions

G.-Y.C., and H.-J.H., designed the experiment, Y.-J.J., implemented an SSVEP stimulator, G.-Y.C., acquired the data, and G.-Y.C., C.-H.H., Y.-J.J., performed data analysis, and H.-J.H., supervised this study. All authors wrote and reviewed the manuscript.

Acknowledgements

This work was supported by the Institute for Information & Communications Technology Planning & Evaluation (IITP) grant funded by the Korea government (No. 2017-0-00451; Development of BCI based Brain and Cognitive Computing Technology for Recognizing User's Intentions using Deep Learning).

Reference

1. Pfurtscheller G, Flotzinger D and Kalcher J. Brain-computer interface-a new communication device for handicapped persons. J Microcinoyt Appl. 1993;16(3):293-9.
2. Wolpaw JR, Birbaumer N, McFarland DJ, Pfurtscheller G and Vaughan TM. Brain-computer interfaces for communication and control. Clin Neurophysiol. 2002;113(6):767-91.
3. Nicolas-Alonso LF and Gomez-Gil J. Brain computer interfaces, a review. Sensors. 2012;12(2):1211-79.
4. Decety J and Ingvar DH. Brain structures participating in mental simulation of motor behavior: A neuropsychological interpretation. Acta psychol. 1990;73(1):13-34.
5. Jeannerod M and Frak V. Mental imaging of motor activity in humans. Curr Opin Neurobiol. 1999;9(6):735-9.
6. Pfurtscheller G, Brunner C, Schlögl A and Da Silva FL. Mu rhythm (de) synchronization and EEG single-trial classification of different motor imagery tasks. NeuroImage. 2006;31(1):153-9.
7. Pfurtscheller G and Neuper C. Motor imagery and direct brain-computer communication. Proc IEEE. 2001;89(7):1123-34.
8. Blankertz B, Muller K-R, Curio G, Vaughan TM, Schalk G, Wolpaw JR, et al. The BCI competition 2003: progress and perspectives in detection and discrimination of EEG single trials. IEEE Trans Biomed Eng. 2004;51(6):1044-51.
9. Blankertz B, Muller K-R, Krusienski DJ, Schalk G, Wolpaw JR, Schlogl A, et al. The BCI competition III: Validating alternative approaches to actual BCI problems. IEEE Trans Neural Syst Rehabil Eng. 2006;14(2):153-9.
10. Cho H, Ahn M, Ahn S, Kwon M and Jun SC. EEG datasets for motor imagery brain computer interface. Gigascience. 2017.
11. Sajda P, Gerson A, Muller K-R, Blankertz B and Parra L. A data analysis competition to evaluate machine learning algorithms for use in brain-computer interfaces. IEEE Trans Neural Syst Rehabil Eng. 2003;11(2):184-5.
12. Tangermann M, Muller K-R, Aertsen A, Birbaumer N, Braun C, Brunner C, et al. Review of the BCI competition IV. Front Neurosci. 2012;6:55.
13. Shin J, von Luhmann A, Blankertz B, Kim D-W, Jeong J, Hwang H-J and Müller K-R.

Open access dataset for EEG+ NIRS single-trial classification. IEEE Trans Neural Syst Rehabil Eng. 2017;25(10):1735-45.

14. BNCI Horizon 2020 Datasets;. Accessed: 2019-03-25. http://bnci-horizon-2020.eu/database/data-sets.

15. Farwell LA and Donchin E. Talking off the top of your head: toward a mental prosthesis utilizing event-related brain potentials. Elecroen Clin Neuro. 1988;70(6):510-23.

16. Sakurada T, Kawase T, Takano K, Komatsu T and Kansaku K. A BMI-based occupational therapy assist suit: asynchronous control by SSVEP. Front Neurosci. 2013;7:172.

17. Kwak N-S, Müller K-R and Lee S-W. A lower limb exoskeleton control system based on steady state visual evoked potentials. J Neural Eng. 2015;12(5):056009.

18. Gollee H, Volosyak I, McLachlan AJ, Hunt KJ and Gräser A. An SSVEP-based brain-computer interface for the control of functional electrical stimulation. IEEE Trans Biomed Eng. 2010;57(8):1847-55.

19. Hwang H-J, Lim J-H, Jung Y-J, Choi H, Lee SW and Im C-H. Development of an SSVEP-based BCI spelling system adopting a QWERTY-style LED keyboard. J Neurosci Methods. 2012;208(1):59-65.

20. Lim J-H, Lee J-H, Hwang H-J, Kim DH and Im C-H. Development of a hybrid mental spelling system combining SSVEP-based brain-computer interface and webcam-based eye tracking. Biomed Signal Process Control. 2015;21:99-104.

21. Wang Y, Chen X, Gao X and Gao S. A benchmark dataset for SSVEP-based brain-computer interfaces. IEEE Trans Neural Syst Rehabil Eng. 2017;25(10):1746-52.

22. Lee M-H, Kwon O, Kim Y-J, Kim H-K, Lee Y-E, Williamson J, et al. EEG Dataset and OpenBMI Toolbox for Three BCI Paradigms: An Investigation into BCI Illiteracy. GigaScience. 2019.

23. Herrmann CS. Human EEG responses to 1-100 Hz flicker: resonance phenomena in visual cortex and their potential correlation to cognitive phenomena. Exp Brain Res. 2001;137(3-4):346-53.

24. Galloway N. Human brain electrophysiology: Evoked potentials and evoked magnetic fields in science and medicine. Br J Ophthalmol. 1990;74(4):255.

25. Volosyak I, Valbuena D, Luth T, Malechka T and Graser A. BCI demographics II: How many (and what kinds of) people can use a high-frequency SSVEP BCI? IEEE Trans Neural

Syst Rehabil Eng. 2011;19(3):232-9.

26. Sakurada T, Kawase T, Komatsu T and Kansaku K. Use of high-frequency visual stimuli above the critical flicker frequency in a SSVEP-based BMI. Clin Neurophysiol. 2015;126(10):1972-8.

27. Cho H, Ahn M, Kim K and Jun SC. Increasing session-to-session transfer in a brain-computer interface with on-site background noise acquisition. J Neural Eng. 2015;12(6):066009

28. Krauledat M, Tangermann M, Blankertz B and Müller K-R. Towards zero training for brain-computer interfacing. PloS one. 2008;3(8):e2967.

29. Samek W, Meinecke FC and Müller K-R. Transferring subspaces between subjects in brain-computer interfacing. IEEE Trans Biomed Eng. 2013;60(8):2289-98.

30. Lin Z, Zhang C, Wu W and Gao X. Frequency recognition based on canonical correlation analysis for SSVEP-based BCIs. IEEE Trans Biomed Eng. 2006;53(12): 2610-2614.

31. Hwang H-J, Kim S, Choi S, Im C-H. EEG-based brain-computer interfaces: a thorough literature survey. Int J Hum-Comput Interact. 2013;29(12): 814-826.

32. Müller SMT, Diez PF, Bastos-Filho TF, Sarcinelli-Filho M, Mut V, Laciar E, and Avila E. Robotic wheelchair commanded by people with disabilities using low/high-frequency ssvep-based BCI. In World Congress on Medical Physics and Biomedical Engineering, 2015. p. 1177-1180.

30. Zhu D, Bieger J, Molina G G, and Aarts R M. A survey of stimulation methods used in SSVEP-based BCIs. Comput Intell Neurosci, 2010; 1.

31. Hwang H J, Kim D H, Han C H, and Im C H. A new dual-frequency stimulation method to increase the number of visual stimuli for multi-class SSVEP-based brain–computer interface (BCI). Brain Res, 2013; 1515: 66-77.

32. Cheng M, Gao X, Gao S, and Xu D. Design and implementation of a brain-computer interface with high transfer rates. IEEE Trans Biomed Eng. 2002; 49(10): 1181-1186.

33. Park S A, Hwang H J, Lim J H, Choi J H, Jung H K, and Im C H. Evaluation of feature extraction methods for EEG-based brain–computer interfaces in terms of robustness to slight changes in electrode locations. Med Biol Eng Comput, 2013;51(5):571-579.

34. Gonzalez-Franco M, Yuan P, Zhang D, Hong B, and Gao S. Motor imagery based brain-computer interface: A study of the effect of positive and negative feedback. In Proceedings of

2011 Annual International Conference of the IEEE Engineering in Medicine and Biology Society, 2011; 6323-6326.

35. Pfurtscheller G, Solis Escalante T, Barry R J, Klobassa D S, Neuper C, and Mueller-Putz, G. Brisk heart rate and EEG changes during execution and withholding of cue-paced foot motor imagery. Front Hum Neurosci. 2013; 7: 379.

36. Gwin J T, Gramann K, Makeig S, and Ferris D P. Removal of movement artifact from high-density EEG recorded during walking and running. J Neurophysiol. 2010; 103(6):3526-3534.

37. O'Regan S, Faul S, and Marnane W. Automatic detection of EEG artefacts arising from head movements using EEG and gyroscope signals. Med Eng Phys. 2013; 35(7):867-874.

38. Vialatte F-B, Maurice M, Dauwels J, Cichocki A. Steady state visual evoked potentials in the delta range (0.5–5 Hz). In Proceedings of 15th International Conference on Advances in Neuro-information Processing. 2009; 400–407.

~~0~~39. Lin Z, Zhang C, Wu W and Gao X. Frequency recognition based on canonical correlation analysis for SSVEP-based BCIs. IEEE Trans Biomed Eng. 2006;53(12): 2610-2614.

40. Barrett K E, Barman S M, Boitano S and Brooks H. Ganong's review of medical physiology. NY: McGraw-Hill Medical. 2009. p. 23.

41. Aladin A I, Whelton S P, Al-Mallah M H, Blaha M J, Keteyian S J, Juraschek S P, et al. Relation of resting heart rate to risk for all-cause mortality by gender after considering exercise capacity (the Henry Ford exercise testing project). Am J Cardiol. 2014;114(11): 1701-1706.

~~31~~42. Hwang H-J, Kim S, Choi S, Im C-H. EEG-based brain-computer interfaces: a thorough literature survey. Int J Hum-Comput Interact. 2013;29(12): 814-826.

~~32~~43. Müller SMT, Diez PF, Bastos-Filho TF, Sarcinelli-Filho M, Mut V, Laciar E, and Avila E. Robotic wheelchair commanded by people with disabilities using low/high-frequency ssvep-based BCI. In World Congress on Medical Physics and Biomedical Engineering, 2015. p. 1177-1180.

Figures

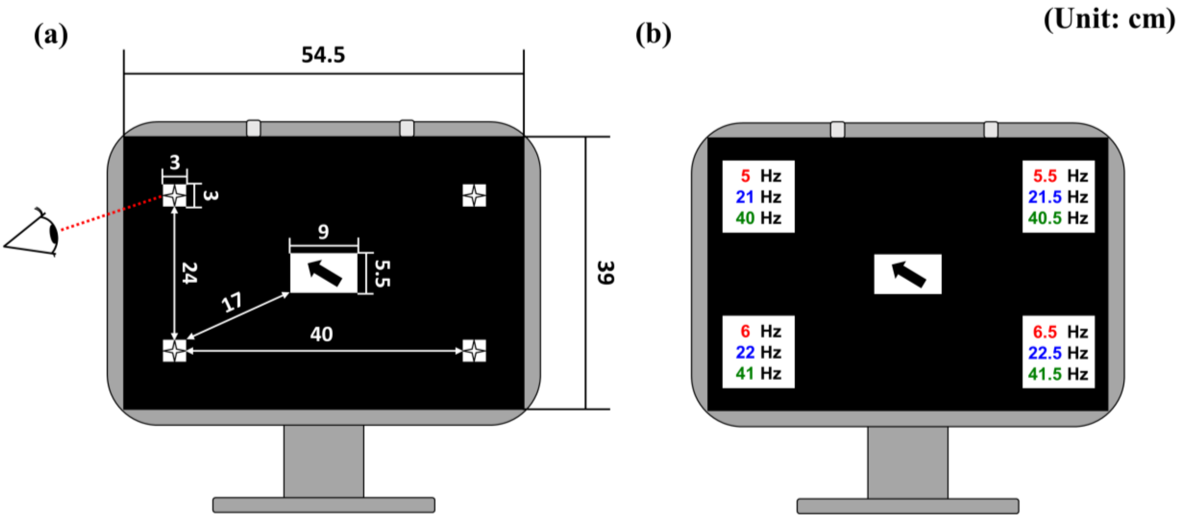

Figure 1. (a) Schematic diagram of the SSVEP stimulator (unit: cm). (b) Placement of four stimulation frequencies for each of three stimulation frequency bands (5, 5.5, 6, and 6.5 Hz for the low frequency band; 21, 21.5, 22, 22.5 Hz for the middle frequency band; 40, 40.5, 41, and 41.5 Hz for the high frequency band).

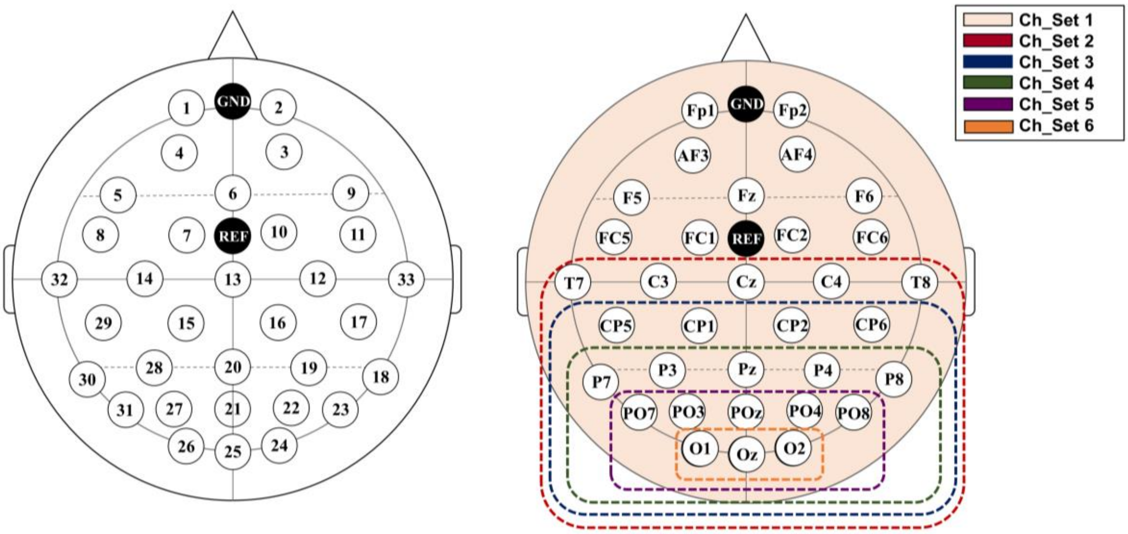

Figure 2. Electrode position used in the experiment with respect to (a) number and (b) position name. Note that six different channel sets are used for data analysis to see the impact of the number of electrodes on classification performance.

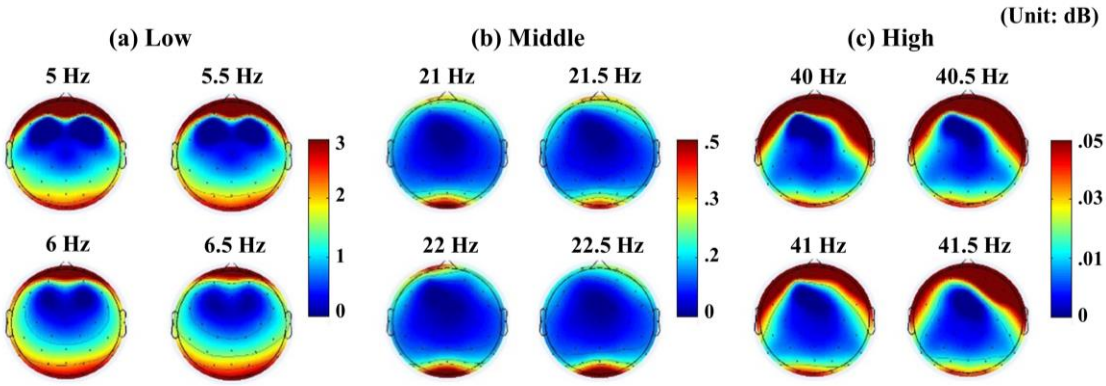

Figure 3. ~~SSVEP topographic maps~~ Topographic maps at the SSVEP frequencies averaged over two days with all subjects for the four stimulation frequencies of (a) the low, (b) middle, and (c) high frequency band.

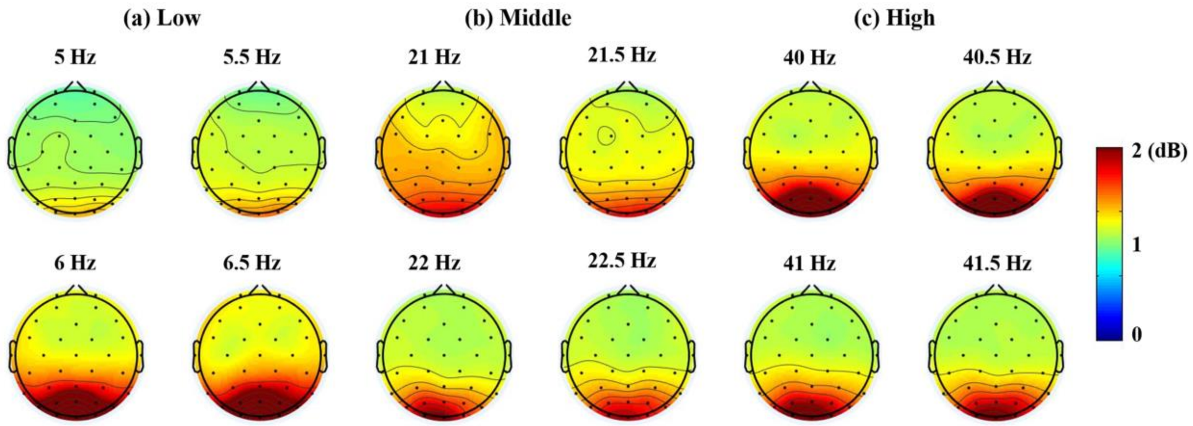

Figure 4. SSVEP SNR topographic maps averaged over two days with all subjects for the four stimulation frequencies of (a) the low, (b) middle, and (c) high frequency band, respectively.

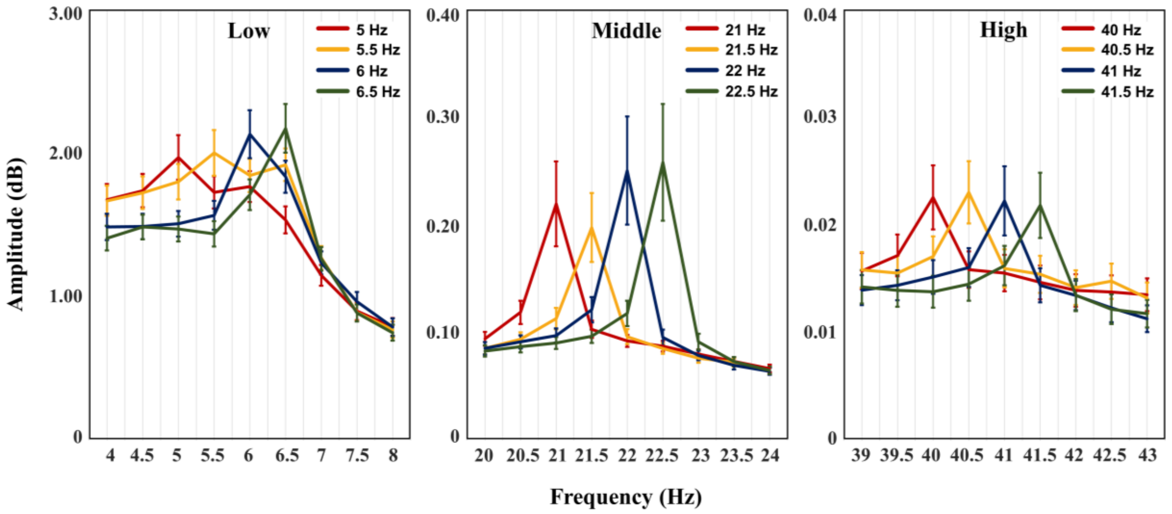

Figure 45. Grand-average SSVEP responses estimated using 13 parieto-occipital channels (Ch_Set4) for each frequency band. Spectral peaks are observed at each of stimulation frequencies. The vertical bars indicate the standard errors of spectral powers for each frequency.

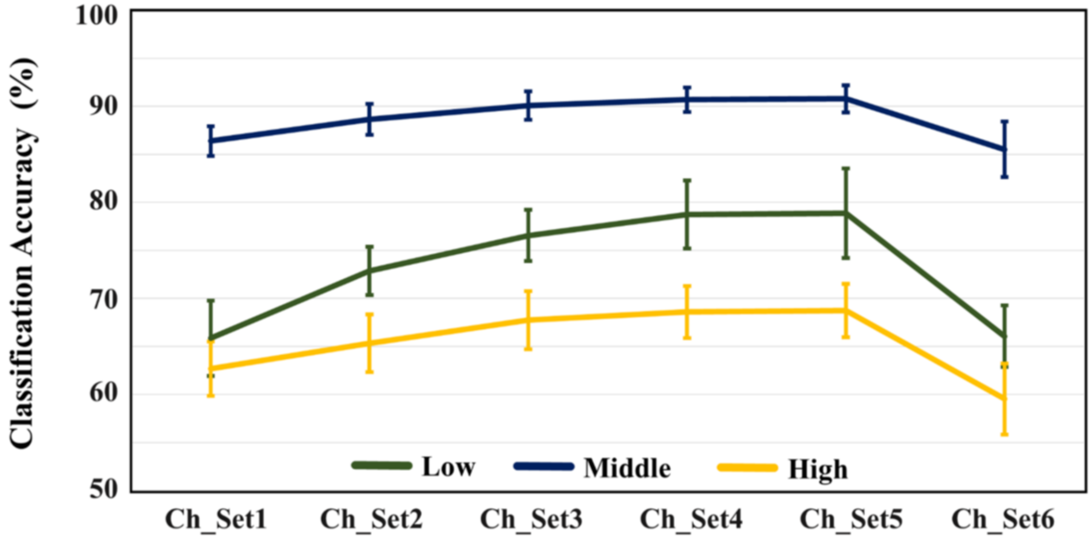

Figure ~~5~~6. Changes in classification accuracy in terms of channel configuration for each stimulation frequency band. Eight channels attached on occipital areas (Ch_Set5) show the highest mean classification accuracy for all of the three frequency bands. The vertical bars indicate the standard deviations of classification accuracies for each channel set.

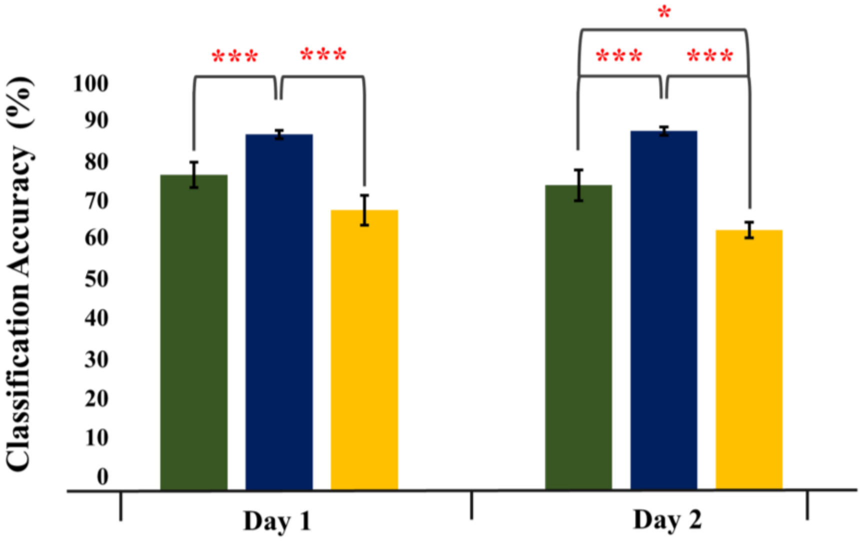

	Day 1	Day 2
Low	0.80 (± 0.03)	0.74 (± 0.04)
Middle	0.90 (± 0.01)	0.91 (± 0.01)
High	0.71 (± 0.04)	0.66 (± 0.02)

Figure ~~6~~7. Mean classification accuracies of three frequency bands for two different days (RM-ANOVA: F(2, 29) = 19.87, p < 0.01; paired t-test Bonferroni corrected p < 0.05: middle > low = high for the first day; RM-ANOVA: F(2, 29) = 23.09, p < 0.01; paired t-test Bonferroni corrected p < 0.05: middle > low > high for the second day). The vertical bars indicated the standard deviations of classification accuracies for each frequency band.

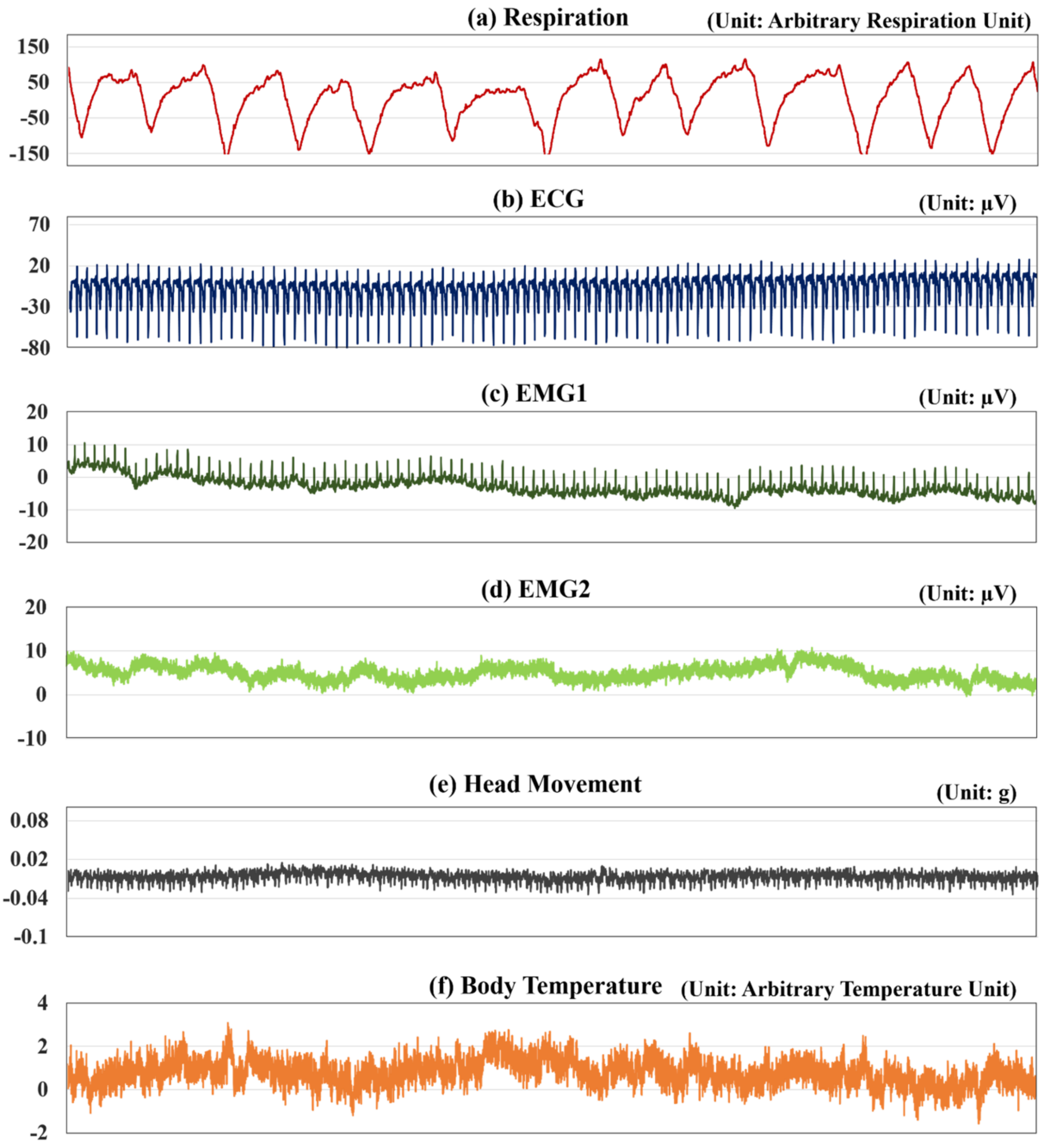

Figure ~~7~~8. Examples of six physiological data with vendor-specific units: (a) respiration [ARU (arbitrary respiration unit)], (b) ECG [µV], (c) EMG1 (left side of the back of the neck) [µV], (d) EMG2 (right side of the back of the neck) [µV], (e) head movement [g ≈ 9.81 m/s2], and (f) body temperature [ARU (arbitrary respiration unit)], respectively.

Click here to access/download
Supplementary Material
Supplementary Figures.docx

Click here to access/download
Supplementary Material
questionnaires_answers.xlsx

Click here to access/download
Supplementary Material
Respiration_Day1.xlsx

Click here to access/download
Supplementary Material
Respiration_Day2.xlsx

Click here to access/download
Supplementary Material
ECG_Day1.xlsx

Click here to access/download
Supplementary Material
ECG_Day2.xlsx

Click here to access/download
Supplementary Material
EMG1_Day1.xlsx

Click here to access/download
Supplementary Material
EMG1_Day2.xlsx

Click here to access/download
Supplementary Material
EMG2_Day1.xlsx

Click here to access/download
Supplementary Material
EMG2_Day2.xlsx

Click here to access/download
Supplementary Material
HeadMovement_Day1.xlsx

Click here to access/download
Supplementary Material
HeadMovement_Day2.xlsx

Click here to access/download
Supplementary Material
Temperature_Day1.xlsx

Click here to access/download
Supplementary Material
Temperature_Day2.xlsx

Click here to access/download
Supplementary Material
SNR_Low(1)_5Hz.xlsx

Click here to access/download
Supplementary Material
SNR_Low(2)_5Hz.xlsx

Click here to access/download
Supplementary Material
SNR_Low(1)_5.5Hz.xlsx

Click here to access/download
Supplementary Material
SNR_Low(2)_5.5Hz.xlsx

Click here to access/download
Supplementary Material
SNR_Low(1)_6Hz.xlsx

Click here to access/download
Supplementary Material
SNR_Low(2)_6Hz.xlsx

Click here to access/download
Supplementary Material
SNR_Low(1)_6.5Hz.xlsx

Click here to access/download
Supplementary Material
SNR_Low(2)_6.5Hz.xlsx

Click here to access/download
Supplementary Material
SNR_Middle(1)_21Hz.xlsx

Click here to access/download
Supplementary Material
SNR_Middle(2)_21Hz.xlsx

Click here to access/download
Supplementary Material
SNR_Middle(1)_21.5Hz.xlsx

Click here to access/download
Supplementary Material
SNR_Middle(2)_21.5Hz.xlsx

Click here to access/download
Supplementary Material
SNR_Middle(1)_22Hz.xlsx

Click here to access/download
Supplementary Material
SNR_Middle(2)_22Hz.xlsx

Click here to access/download
Supplementary Material
SNR_Middle(1)_22.5Hz.xlsx

Click here to access/download
Supplementary Material
SNR_Middle(2)_22.5Hz.xlsx

Click here to access/download
Supplementary Material
SNR_High(1)_40Hz.xlsx

Click here to access/download
Supplementary Material
SNR_High(2)_40Hz.xlsx

Click here to access/download
Supplementary Material
SNR_High(1)_40.5Hz.xlsx

Click here to access/download
Supplementary Material
SNR_High(2)_40.5Hz.xlsx

Click here to access/download
Supplementary Material
SNR_High(1)_41Hz.xlsx

Click here to access/download
Supplementary Material
SNR_High(2)_41Hz.xlsx

Click here to access/download
Supplementary Material
SNR_High(1)_41.5Hz.xlsx

Click here to access/download
Supplementary Material
SNR_High(2)_41.5Hz.xlsx

 Click here to access/download;Personal Cover;Responses to the reviewers.docx

Responses to Reviewers' Comments

Ref#: GIGA-D-19-00116

Title: A multi-day and multi-band dataset for steady-state visual evoked potential-based brain-computer interface

Authors: Ga-Young Choi, Chang-Hee Han, Young-Jin Jung, and Han-Jeong Hwang

Dear Reviewers,

We are very grateful to the reviewers for their constructive comments. We have substantially revised our original manuscript according to the comments and suggestions the reviewers made. Below, we summarize how we have addressed the review comments. The review comments are printed in normal font and responses in blue-bold font. The revised expressions and paragraphs are emphasized with fluorescent color in the revised manuscript.

Reviewer #1:

Comments to the Author

The manuscript reports an EEG-based SSVEP BCI dataset. Compared to existing datasets, the present dataset has its unique features of multi-band (covering typical SSVEP frequency bands), multi-session, and multi-day recordings. The number of participants is reasonable. Data analysis supports the validity of the dataset. Due to the limited number of publicly available SSVEP BCI datasets as compared to the popularity of the SSVEP BCI applications, the reported dataset is timely and could have important contribution to the BCI research community. Therefore, I suggest acceptance of the manuscript, with the following minor comments.

: We appreciate the reviewer's favorable comments. We have revised our original manuscript based on the reviewer's comments.

1. While I agree thirty subjects is reasonable for a dataset, it would be clearer to have some explanations on this. And, whether they had previous (SSVEP) BCI experiences should be reported.

: Thank you for your suggestion. It is well documented that a sample size of 30 is big

enough for the theoretical distribution of the sample mean to be distributed roughly normally [1, 2], and thereby providing us with a rationale to apply parametric statistical tests that have more statistical power than non-parametric ones. Based on the theory, we decided to recruit 30 subjects for this study even though the number of subjects is smaller than the previous two SSVEP studies (35 subjects and 54 subjects). Thus, we believe that thirty subjects are enough to validate analysis results obtained using our SSVEP dataset from a statistical point of view. To provide the mentioned information, we inserted the following sentences in the Subject section:

> The number of subjects was decided because a sample size of 30 is enough to apply parametric statistical tests for analysis results. Note that parametric statistical tests provide more statistical power than non-parametric ones, thereby ensuring more reliable validation of our SSVEP dataset.

Seven of thirty subjects had experience on BCI experiment, but they participated in endogenous BCI experiments, not SSVEP experiments. Thus, we think that their BCI experience would not significantly influence experimental results. To provide the mentioned information, we inserted new sentences in the Subject section, as follows:

> Seven of thirty subjects had prior BCI experience, but they participated in endogenous BCI experiments based on a mental arithmetic task. Thus, it is assumed that their prior BCI experience would not significantly affect research results.

We also included whether each subject had BCI experience or not in the supplementary file (questionnaires_answers.xlsx) that contain the answers of the questionnaires.

2. Please report the duty cycle of the steady-state stimulations: I assume 50% on and 50% off?

: As the reviewer thought, a duty cycle of the stimulation was 50% on and 50% off. We provided this information in the Stimulator section, as follows:

> A duty cycle was set at 50%, meaning that an LED has 50% on-time and 50% off-time.

3. The selection of the frequency bands need further explanation. Especially, why not the alpha band included?

: Thank you for your constructive comment. The selection of the three frequency bands was based on a previous study [3], where the stimulus frequencies were classified into

three frequency bands: low (1-12 Hz), middle (12-30 Hz), and high (30-60 Hz).

Our principle to select stimulation frequencies for each band was to avoid that harmonic frequencies of four stimulation frequencies in the low frequency band are not overlapped with those in the middle and high frequency bands. Also, this principle was applied to select four stimulation frequencies for the middle frequency band; the four stimulation frequencies in the middle frequency are not overlapped with those in the high frequency band. This was because simultaneous use of harmonic frequencies could significantly decrease the performance of SSVEP-based BCI systems [3].

We excluded the alpha band for SSVEP stimulation because its use can produce a considerable number of false positives [4, 5] even though alpha band for SSVEP stimulation shows high signal-to-noise ratio.

In order to meet all the mentioned conditions, the four stimulation frequencies used in this study was selected for each frequency band. To provide the mentioned information, we revised relevant sentences in the Stimulator section, as follows:

> **(original)** As mentioned above, three different frequency bands were independently used for SSVEP stimulation to get multi-band SSVEP datasets in this study. Three sets of four stimulation frequencies selected for each frequency band were as follows: 5, 5.5, 6, and 6.5 Hz for the low frequency band; 21, 21.5, 22, 22.5 Hz for the middle frequency band; 40, 40.5, 41, and 41.5 Hz for the high frequency band. We assigned four stimulation frequencies to four LEDs, depending on the stimulation frequency band, as shown in figure 1(b).

> **(revised)** As mentioned above, three different frequency bands (low: 1-12 Hz, middle: 12-30 Hz, and high: 30-60 Hz [30]) were independently used for SSVEP stimulation to get multi-band SSVEP datasets in this study. Three sets of four stimulation frequencies selected for each frequency band were as follows: 5, 5.5, 6, and 6.5 Hz for the low frequency band; 21, 21.5, 22, 22.5 Hz for the middle frequency band; 40, 40.5, 41, and 41.5 Hz for the high frequency band. The four stimulation frequencies for each frequency band were selected in such a way that the harmonic frequencies of four frequencies in the low frequency band are not overlapped with four frequencies in each of the middle and high frequency bands, and the harmonic frequencies of four frequencies in the middle frequency band are not overlapped with four frequencies in

the high frequency band. This was because simultaneous use of harmonic frequencies as different stimulation frequencies could significantly decrease the performance of SSVEP-based BCI systems [31]. Also, alpha frequency band was not considered because its use can produce a considerable number of false positives [30, 32] even though alpha band for SSVEP stimulation shows high signal-to-noise ratio. We assigned four stimulation frequencies to four LEDs, depending on the stimulation frequency band, as shown in figure 1(b).

4. The rationale for the inclusion of the bio-signals (ECG, EMG etc.) should be given prior to the Results session, preferably with references to previous related studies.

: Thank you for the useful comment. Based on the reviewer's recommendation, we added relevant sentences with proper references in the Data Recording section prior to the Results section in order to provide the rationale for the inclusion of various bio-signals:

The physiological data can be used for investigating the relation between changes in brain activity and physiological condition as well as for developing artifact correction algorithms. For example, some previous studies used EEG and ECG simultaneously to check mental status and stress level/mental effort [34, 35], and others used motion data to remove motion-related artifacts from EEG [36, 37].

5. Since there were two recording days, what measures have the authors taken to control possible position differences of the EEG recording sites across days?

: We did not use a specific measure or method to control possible position changes in EEG recording electrodes between two different days, but instead we tried to maintain EEG measurement condition for each subject across different days as much as possible to avoid significant changes in electrode location. This is because we intended to make natural changes in electrode locations between two different days, which would happen in daily BCI use, thereby providing useful dataset to study session-to-session (day-to-day) transfer issues. Note that electrode location change between different days is one of the main factors that cause the non-stationarity of EEGs between different days. We clarified how we measured EEG data for two different days, in particular, from the electrode location point of view in the Data Recording section, as follows:

We did not control changes in electrode locations between two different days, but instead we tried to maintain the condition of EEG measurement between two different days for each subject. This was because to naturally make slight changes in electrode locations, which would happen in daily BCI use, and thereby providing useful dataset to study session-to-session (day-to-day) transfer issues. Note that electrode location change between different days is an important factor that cause EEG non-stationarity between different days [33].

6. Figure 3: it is misleading to state 'SSVEP topographic maps', as the authors already mentioned in the main text, the frontal activation could be eye movement related activities that happened to be at the steady-state stimulation frequencies. It could be better to state 'Topographic maps at the SSVEP frequencies'.

: Thank you for your kind suggestion. We revised the original manuscript accordingly, as follows:

(original) Figure 3 shows **SSVEP topographic maps** averaged over two days with all subjects for the four stimulation frequencies of each frequency band.

(revised) Figure 3 shows **topographic maps at the SSVEP frequencies** averaged over two days with all subjects for the four stimulation frequencies of each frequency band.

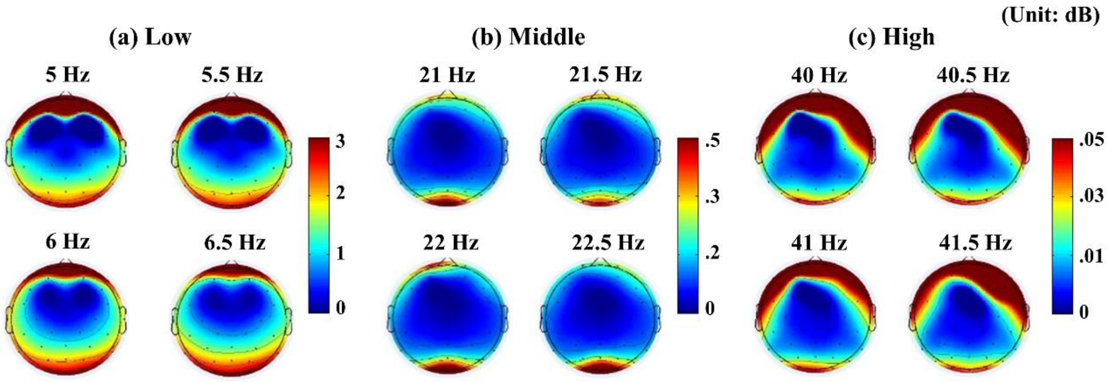

Figure 3. **Topographic maps at the SSVEP frequencies** averaged over two days with all subjects for the four stimulation frequencies of (a) the low, (b) middle, and (c) high frequency band.

Reviewer #2:

Comments to the Author

This data note is about steady-state visual evoked potential (SSVEP) dataset for brain-computer interface (BCI) research field. The dataset includes multiple days recording with multiple frequencies for visual stimulation and physiological data including respiration, electrocardiography, electromyography, head movement, and body temperature. This dataset well aimed challenges in BCI research, such as session variability and subject variability. The dataset is reasonable to be published in GigaScience, however, the manuscript needs major revision to be published as following comments:

: We appreciate the reviewer's favorable comments. We have revised our original manuscript according to the comments and suggestions of the reviewer.

1. There is no validation results for number of bad trials/channels/subjects/day-sessions in the datasets. Even if there are no bad trials/channels/subjects/day-sessions, author should prove the dataset is clean, for an example, by using signal-to-noise ratio (SNR) between background noise and SSVEP in EEG.

: Thank you for your constructive comment. As advised, to quantitatively prove the data quality, we calculated SSVEP SNRs for each channel/trial/subject/day, and provided grand-average topographic maps for each stimulation frequency in terms of SSVEP SNR as Figure 4 in the revised manuscript. As shown in Figure 4, SSVEP SNRs are higher than 1 for most channels in each frequency band, meaning that SSVEP responses are higher than background EEG. In particular, as expected, occipital areas show highest SSVEP SNRs, which are more than 2. We believe that the SSVEP SNR result could prove the reliability of our SSVEP dataset.

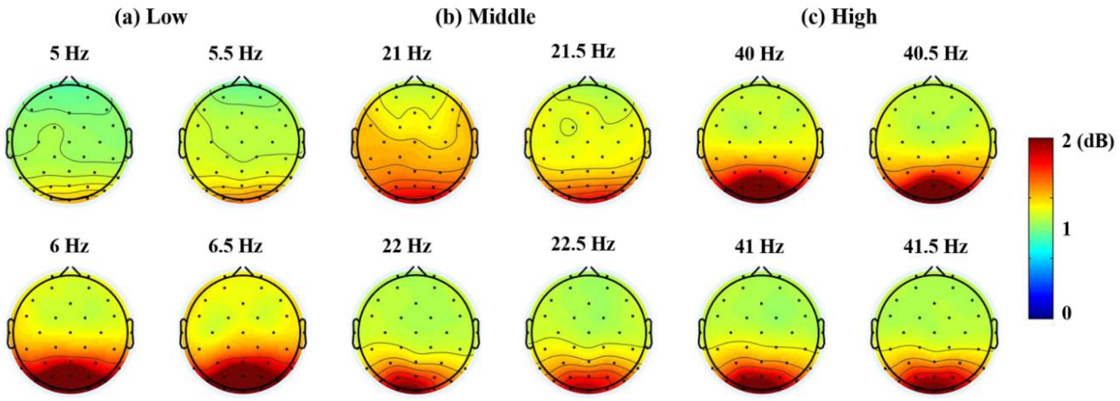

Figure 4. SSVEP SNR topographic maps averaged over two days with all subjects for the four stimulation frequencies of (a) the low, (b) middle, and (c) high frequency band, respectively.

Because it is too vast to report all SSVEP SNRs for each channel/trial/subject/day in the manuscript, we provide them as supplementary files. There are 12 supplementary files (4 stimulation frequencies x 3 frequency bands) for each day, and each file contains SSVEP SNRs of each channel and each trial for all subjects. We did not define bad recordings (e.g., which one is bad channel or trial), but provide SSVEP SNRs as they are because there is no standard rule to define bad recording for SSVEP and the definition of bad recordings would depend on the goal of a research. Thus, we believe that researchers who will use our SSVEP dataset can define bad recordings by themselves based on their research goals. We inserted a new paragraph to provide the mentioned information with the new figure showing SSVEP SNRs in the Results section, as follows:

> Figure 4 shows SSVEP SNR topographic maps averaged over two days with all subjects for the four stimulation frequencies of each frequency band. Most channels show SSVEP SNRs higher than 1 for all stimulation frequencies, and in particular parieto-occipital channels show high SSVEP SNRs more than 2, demonstrating the reliability of our SSVEP datasets. All SSVEP SNRs are provided with 12 supplementary files (4 stimulation frequencies x 3 frequency bands) for each day, and each supplementary file contains SSVEP SNRs of each channel and each trial for all subjects.

We also inserted the following sentence to provide the information how to calculate SSVEP SNRs in the Methods section:

> The SSVEP SNR was also calculated by dividing the mean spectral amplitude of 6 adjacent frequencies from the SSVEP amplitude at the stimulation frequency to demonstrate the reliability of our SSVEP dataset [38].

2. There is no proof/validation for physiological data is reasonable, for example, checking number of in-/ex-hale, heart beating rates, noise level of EMG, and body temperature in normal range. There is no information in the y-axis of Figure 7 about the unit for each physiological measurement, for example, unit of head movement data and body temperature.

: Thanks for the constructive comment. We validated each of physiological data in detail; respiration rate, heart rate, range of EMG, head movement, and body temperature values. Except few cases, most results were reasonable; respiration rate and heart (ECG) rate were in the normal range, and the ranges of EMG, IMU, body temperature values were

also acceptable. All detailed results are provided with 6 supplementary figures and 12 supplementary files (6 biosiganls x 2 days).

1) Respiration rate and heart rate

: we estimated respiration rates and heart rates for each session and each stimulation frequency band, and averaged them for each subject, which was repeated for two different days. As a result, most of respiration rates and heart rates fall in the normal range of 12–18 breaths per minute [reference no. 40 in the manuscript] and 60–100 beats per minute [reference no. 41 in the manuscript].

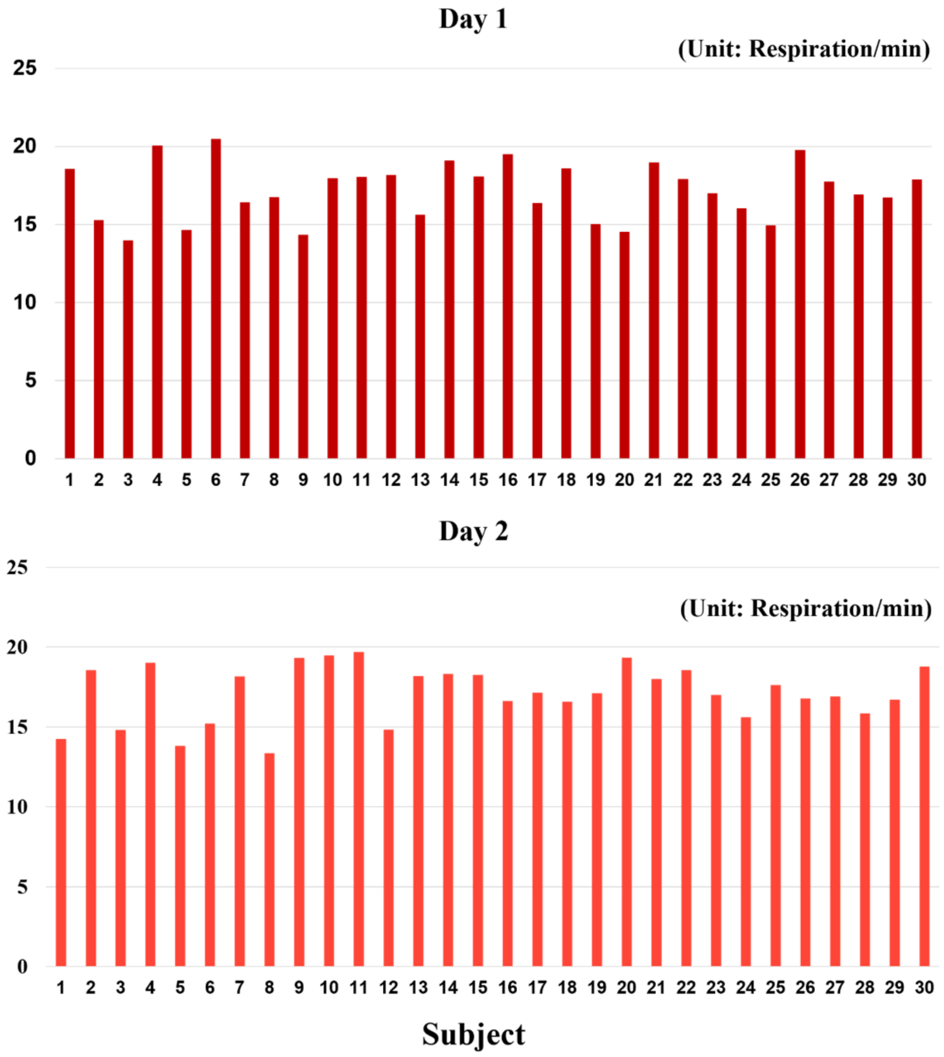

Supplementary Figure 1. Respiration rates of each subject for two days. For detailed information, refer to the corresponding supplementary files (Respiration_Day1.xlxs and Respiration_Day2.xlsx).

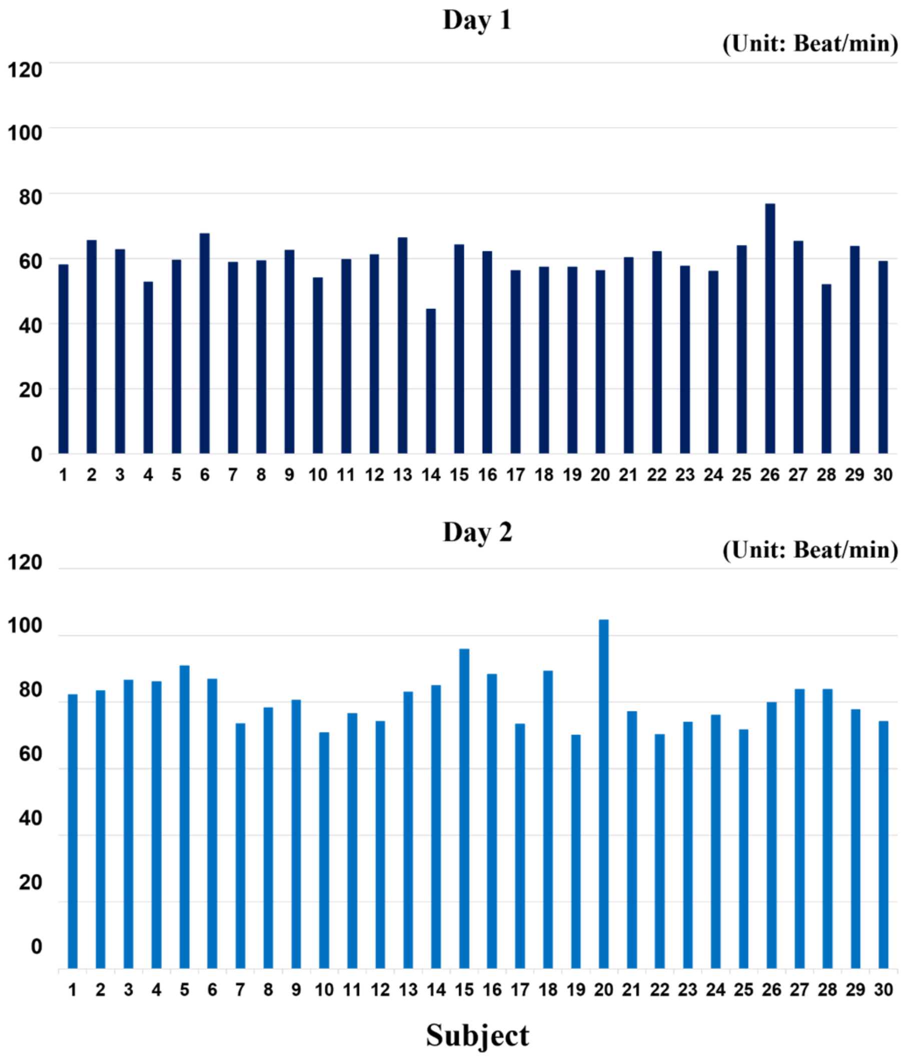

Supplementary Figure 2. Heart rates of each subject for two days. For detailed information, refer to the corresponding supplementary files (ECG_Day1.xlxs and ECG_Day2.xlsx).

2) EMG1 and EMG2 for neck movement

: We calculated the means and standard deviations of each trial for two EMG channels, and then plotted the mean values of all trials with a box-plot to see the range of EMG values. Most subjects showed mean EMG values of lower than 20 µV for most trials. Considering that an amplitude more than 50 µV is regarded as artifacts in terms of EEG [6], our results demonstrates that little neck movement was generally induced during the whole experiment.

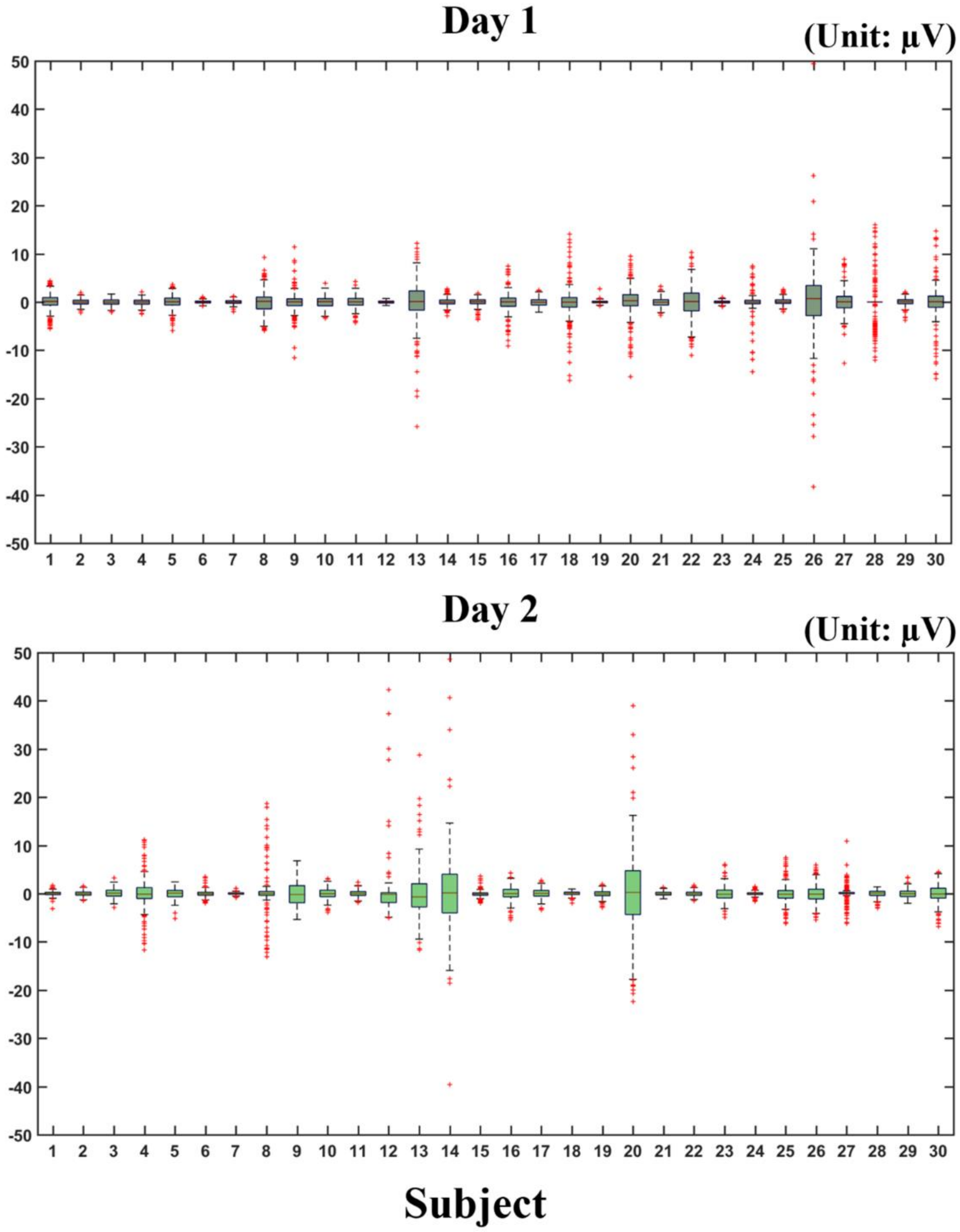

Supplementary Figure 3. Mean EMG values of each trial for each subject estimated from the EMG1 channel. For detailed information, refer to the corresponding supplementary files (EMG1_Day1.xlxs and EMG1_Day2.xlsx).

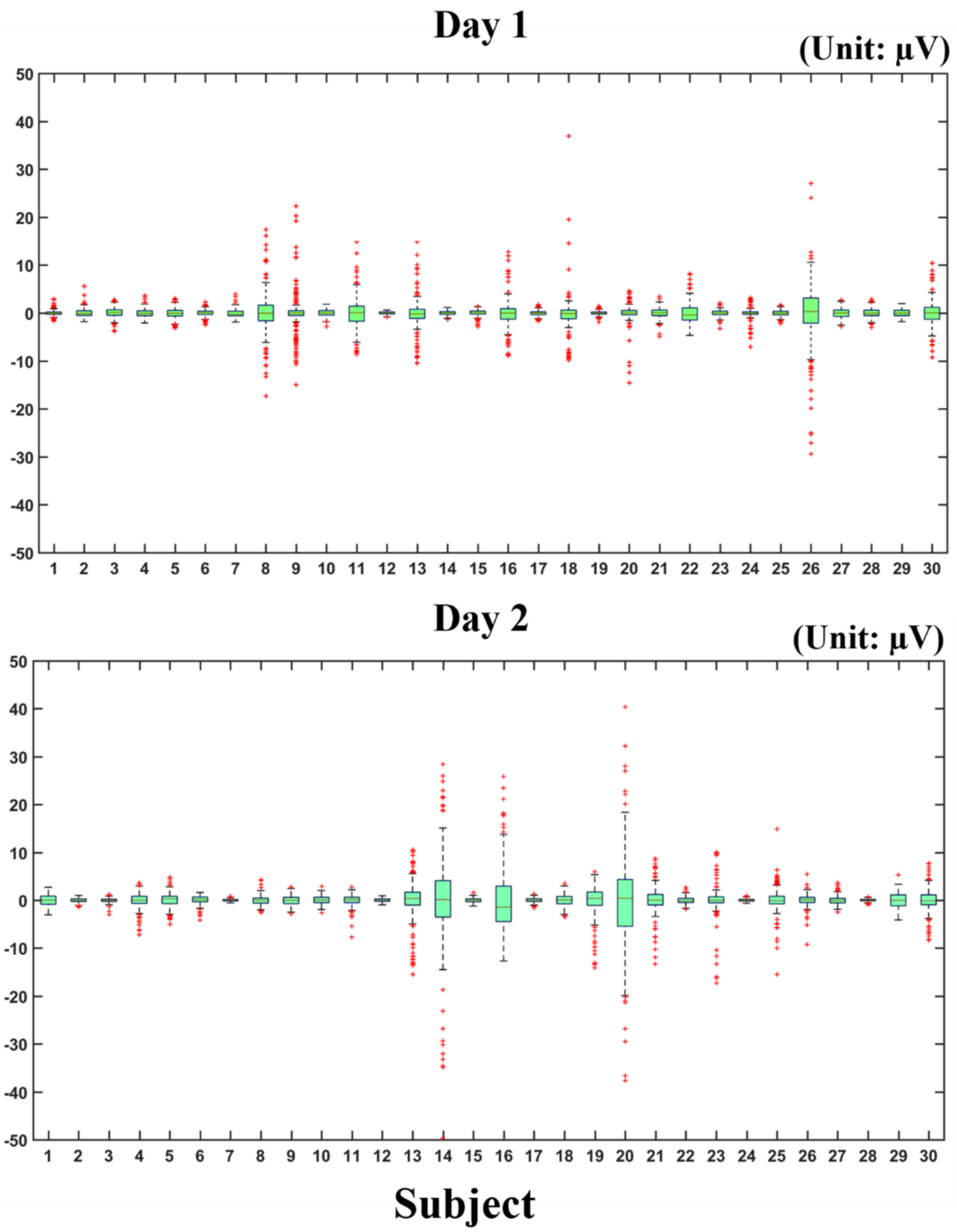

Supplementary Figure 4. Mean EMG values of each trial for each subject estimated from the EMG2 channel. For detailed information, refer to the corresponding supplementary files (EMG2_Day1.xlxs and EMG2_Day2.xlsx).

3) IMU sensor for head movement

: We calculated the means and standard deviations of each trial for the IMU sensor (unit: g ≈ 9.81 m/s^2), and then plotted the mean values of all trials with a

box-plot to see the range of head movement. As shown below, IMU values are barely changed for most subjects, meaning that little head movement is generated during the whole experiment.

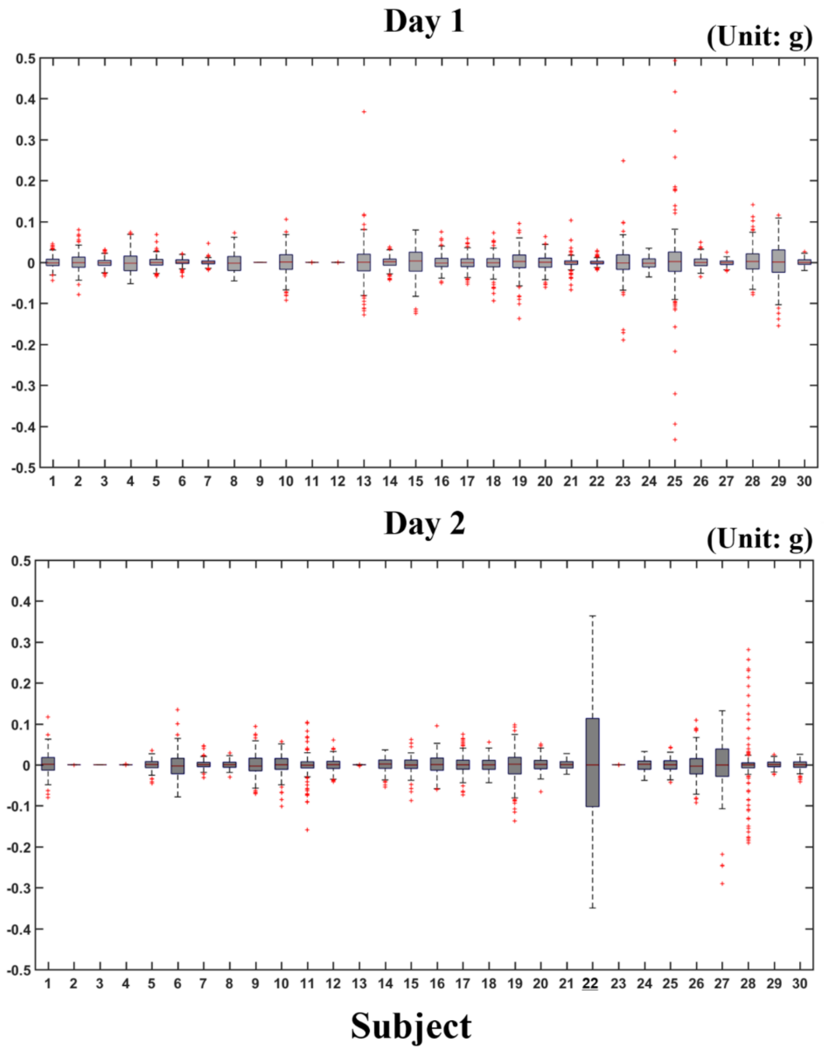

Supplementary Figure 5. Mean IMU values of each trial for each subject for two days. For detailed information, refer to the corresponding supplementary files (HeadMovement_Day1.xlxs and HeadMovement_Day2.xlsx).

4) **Body temperature**

: As the EMG and IMU sensors, we calculated the means and standard deviations of each trial for a temperature sensor, and then plotted the mean values of all trials with a box-plot to see the range of body temperature. Note that relative changes

in body temperature are provided, not absolute changes in degree Celsius due to technical issues that reasonable temperature information in degree Celsius could not be obtained despite of following the instruction provided by the EEG company. We believe that body temperature information can be still usefully used to see the correlation between brain activity and body temperature even though relative changes in temperature are provided. Except some subjects (e.g., subjects 9 and 30 for day 1; subjects 2, 5, and 23 for day 2), most subjects showed relative changes in body temperature less than 50.

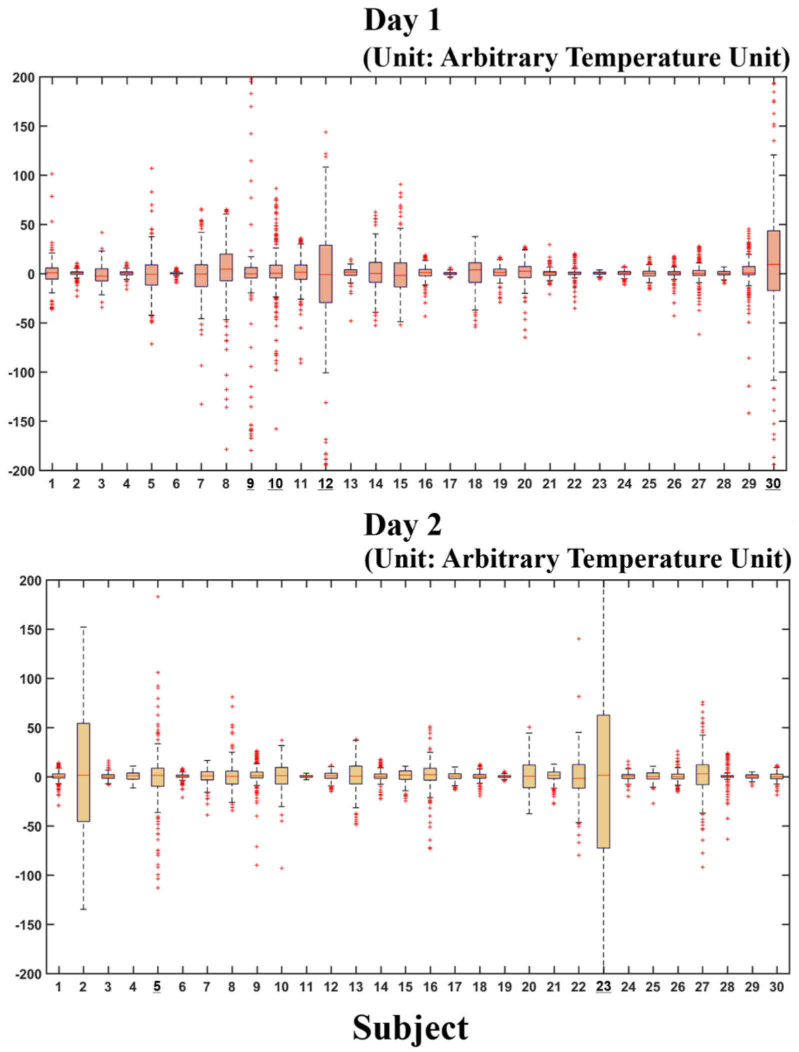

Supplementary Figure 6. Mean body temperatures of each trial for each subject for two days. The subject numbers underlined indicate that there are outliers outside of the range of y-axis limits. For detailed information, refer to the corresponding supplementary files (Temperature_Day1.xlxs and Temperature_Day2.xlsx).

According to the reviewer's advice, we specified the units of each of physiological data in Figure 7 (Figure 8 in the revised manuscript). Also, we extended the time period (x-axis) of the example results from 6 s to 60 s (1 min) to provide more intuitive examples for biosignals (e.g., respiration rate and heart rate). Based on the figure 8 changed, we revised the relevant paragraph, as follows:

> **(original)** Example of six physiological data measured with EEGs are presented in figure 7. Because physiological data show high inter- and intra-subject variability, representative examples are provided for each of six physiological data. The example data were measured from S2 when the subject focused on an LED modulated at 5 Hz for 6 s during the first trial. In particular, two breathings and seven heartbeats are clearly observed for 6 s from respiration (figure 7(a)) and ECG data (figure 7(b)), respectively. Two EMG (figures 7(c) and (d)) and head motion (figure 7(e)) data show that no significant movement was made during the first trial, and heartbeats are also observed from two EMG data (figures 7(c) and (d)). Body temperature monotonically increases, but not significant (figure 7(f)).

> **(revised)** Example of six physiological data measured with EEGs are presented in **figure 8**. Because physiological data show high inter- and intra-subject variability, representative examples are provided for each of six physiological data, and **detailed results are provided with 6 supplementary figures and 12 supplementary files.** The example data were measured from S2 when the subject started to focus on an LED modulated at 5 Hz from the first trial **for 60 s**. In particular, **13 breathings and 93 heartbeats are clearly observed for 60 s from respiration (figure 8(a)) and ECG data (figure 8(b)), respectively, which fall into the normal ranges of adult respiration rate (12 – 18) [40] and heart rate (60 – 100) [41].** Two EMG (figures 8(c) and (d)) and head motion (figure 8(e)) data show that no significant movement was made, and heartbeats are also observed from two EMG data (figures 8(c) and (d)). Body temperature also shows little changes (figure 8(f)). Most subjects showed similar trends for each of physiological data, except few cases (see supplementary figures and files).

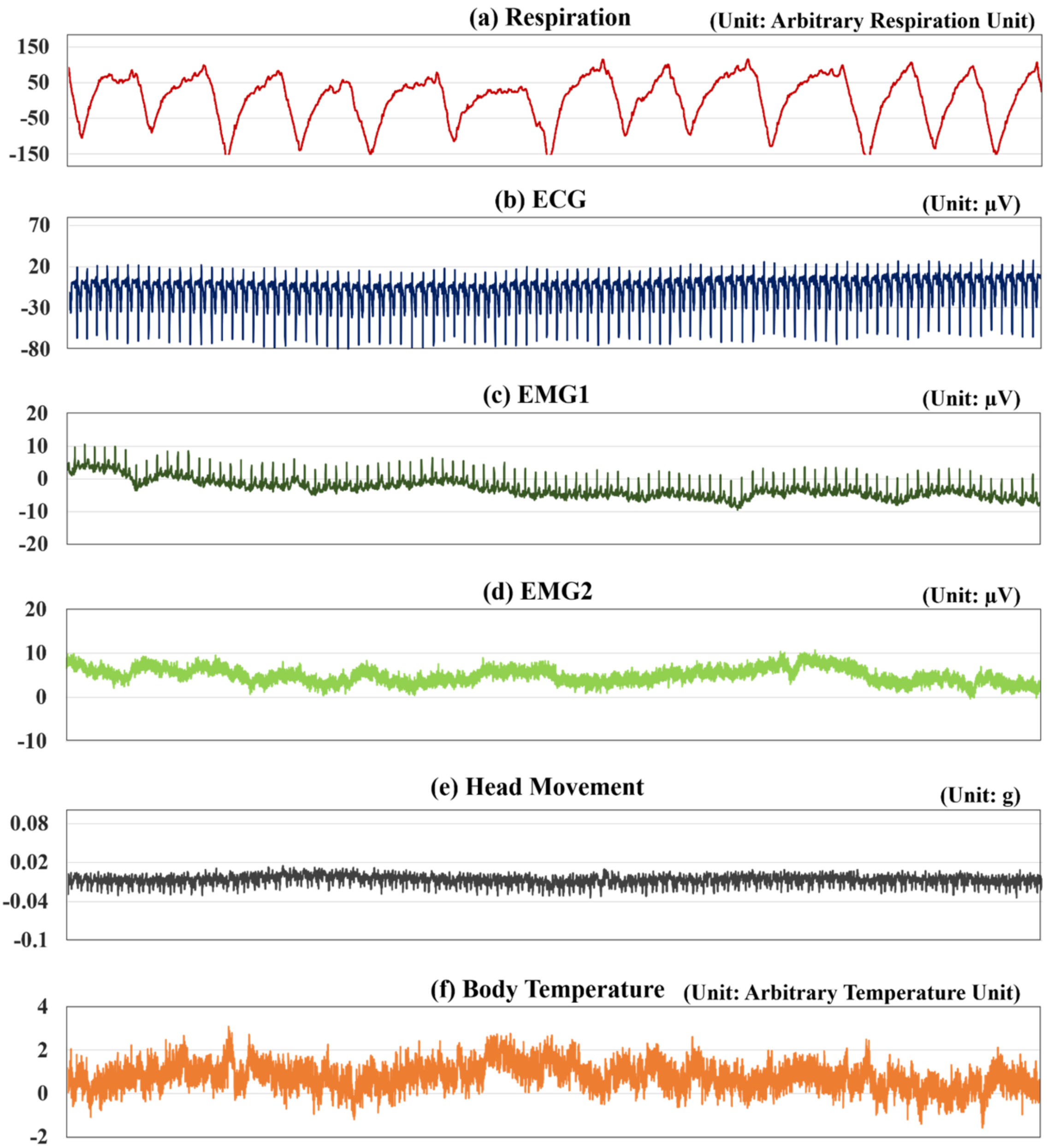

Figure 8. Examples of six physiological data with vendor-specific units: (a) respiration [ARU (arbitrary respiration unit)], (b) ECG [µV], (c) EMG1 (left side of the back of the neck) [µV], (d) EMG2 (right side of the back of the neck) [µV], (e) head movement [g ≈ 9.81 m/s^2], and (f) body temperature [ARU (arbitrary respiration unit)], respectively.

We inserted a new paragraph to explain how to analyze physiological data in the Methods section, as follows:

Each of six physiological data was linearly detrended to remove baseline drift. Respiration rate and heart rate were estimated from respiration and ECG data,

respectively, based on peak information for each frequency band and each session, to check the ranges of respiration and heart rates. The mean and standard deviation values were estimated for each trial for the other physiological data (EMG1, EMG2, IMU, and body temperature) to see changes in each of the four physiological data.

We also revised the Results section based on the revised Figure 8, as follows:

(original) Example of six physiological data measured with EEGs are presented in figure 7. Because physiological data show high inter- and intra-subject variability, representative examples are provided for each of six physiological data. The example data were measured from S2 when the subject focused on an LED modulated at 5 Hz for 6 s during from the first trial. In particular, two breathings and seven heartbeats are clearly observed for 6 s from respiration (figure 7(a)) and ECG data (figure 7(b)), respectively. Two EMG (figures 7(c) and (d)) and head motion (figure 7(e)) data show that no significant movement was made during the first trial, and heartbeats are also observed from two EMG data (figures 7(c) and (d)). Body temperature monotonically increases, but not significant (figure 7(f)).

(revised) Example of six physiological data measured with EEGs are presented **in figure 8**. Because physiological data show high inter- and intra-subject variability, representative examples are provided for each of six physiological data, **and detailed results are provided with 12 supplementary files (e.g., ECG_Day1.xlsx)**. The example data were measured from S2 when the subject started to focus on an LED modulated at 5 Hz **from the first trial for 60 s.** In particular, **13 breathings and 93 heartbeats are clearly observed for 60 s from respiration (figure 8(a)) and ECG data (figure 8(b)), respectively, which fall into the normal ranges of adult respiration rate (12 – 18) [40] and heart rate (60 – 100) [41].** Two EMG (figures 8(c) and (d)) and head motion (figure 8(e)) data show that no significant movement was made, and heartbeats are also observed from two EMG data (figures 8(c) and (d)). Body temperature also shows little changes (figure 8(f)).

Minor comments:

1. For necessary controls, recording date and time for each day-session data should be specified. Further, the EEG cap positions on multiple days cannot be completely same for each subject. Author should mention the reasonable method to minimize the problem in this manuscript.

According to the reviewer's recommendation, we added recording date and time for each subject into the supplementary file (questionnaires_answers.xlsx).

We did not use a specific measure or method to control possible position changes in EEG recording electrodes between two different days, but instead we tried to maintain EEG measurement condition for each subject across different days as much as possible to avoid significant changes in electrode location. This is because we intended to make natural changes in electrode locations between two different days, which would happen in daily BCI use, thereby providing useful dataset to study session-to-session (day-to-day) transfer issues. Note that electrode location change between different days is one of the main factors that cause the non-stationarity of EEGs between different days. We clarified how we measured EEG data for two different days, in particular, from the electrode location point of view in the Data Recording section, as follows:

> We did not control changes in electrode locations between two different days, but instead we tried to maintain the condition of EEG measurement between two different days for each subject. This was because to naturally make slight changes in electrode locations, which would happen in daily BCI use, and thereby providing useful dataset to study session-to-session (day-to-day) transfer issues. Note that electrode location change between different days is an important factor that cause EEG non-stationarity between different days [33].

2. There are some typos and wrong word in the manuscript.

* Findings paragraph in Abstract - 'reasonalbe'
* Page #4 line #22 - 'choie'
* Page #11 line #6 and A-7 in Table 2 - 'Drug'

In general, 'drug' means illegal medicine, such as marijuana, so, 'medicine' would be correct one.

: Thank you for your kind comment. We corrected the mistakes we made.

3. There is no detailed explanation for the unit of color map in Figure 3 & Font sizes are different in Figure 3 (c).

: Thank you for your detailed comment. We inserted the unit of color map in Figure 3 and corrected the figure based on the reviewer's comment.

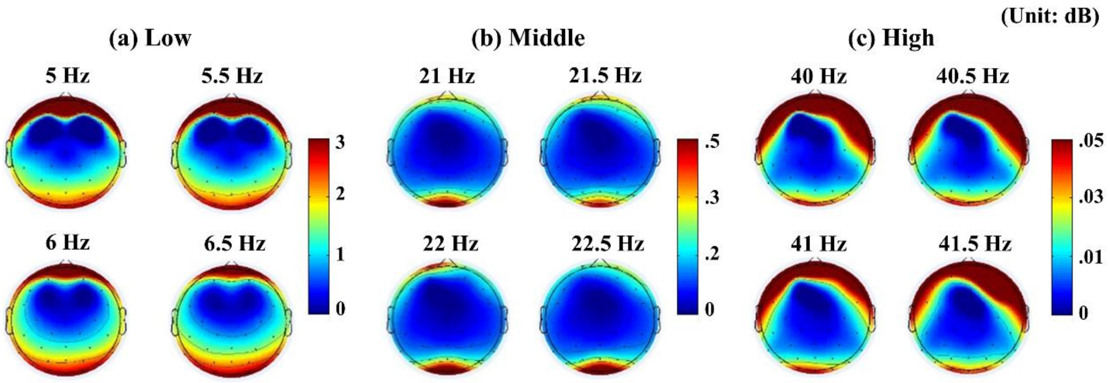

Figure 3. Topographic maps at the SSVEP frequencies averaged over two days with all subjects for the four stimulation frequencies of (a) the low, (b) middle, and (c) high frequency band.

References

1. Hesterberg T C. What teachers should know about the bootstrap: Resampling in the undergraduate statistics curriculum. The Amer Stat, 2015; 69(4):371-386.

2. Moses L E, and Moses L E. Think and explain with statistics. Reading, MA: Addison-Wesley. 1986; 199-203.

3. Hwang H J, Kim D H, Han C H, and Im C H. A new dual-frequency stimulation method to increase the number of visual stimuli for multi-class SSVEP-based brain–computer interface (BCI). Brain Res. 2013; 1515: 66-77.

4. Zhu D, Bieger J, Molina G G, and Aarts R M. A survey of stimulation methods used in SSVEP-based BCIs. Comput Intell Neurosci, 2010; 1.

5. Cheng M, Gao X, Gao S, and Xu D. Design and implementation of a brain-computer interface with high transfer rates. IEEE Trans Biomed Eng. 2002; 49(10):1181-1186.

6. Choi S I, and Hwang H J. Effects of Different Re-referencing Methods on Ear-EEG. Front Neurosci. 2019; 13: 882.
