# Supplementary material for: A multi-day and multi-band dataset for a steady-state visual-evoked potential–based brain-computer interface
Source: Gigascience. 2019 Nov 25;8(11):giz133. doi: 10.1093/gigascience/giz133 (PMC6876666; doi:10.1093/gigascience/giz133)
Supplement: giz133_GIGA-D-19-00116_Revision_2 [file giz133_giga-d-19-00116_revision_2.pdf]

GigaScience

A multi-day and multi-band dataset for a steady-state visual-evoked potential-based brain-computer interface

--Manuscript Draft--

**Manuscript Number:**	GIGA-D-19-00116R2
**Full Title:**	A multi-day and multi-band dataset for a steady-state visual-evoked potential-based brain-computer interface
**Article Type:**	Data Note
**Funding Information:**	Institute for Information and Information & Communications Technology Planning & Evaluation (2017-0-00451) — Prof. Han-Jeong Hwang
**Abstract:**	Background : A steady-state visual-evoked potential (SSVEP) is a brain response to visual stimuli modulated at certain frequencies; it has been widely used in electroencephalography (EEG)-based brain–computer interface (BCI) research. However, there are few published SSVEP datasets for BCIs. In this study, we obtained a new SSVEP dataset based on measurements from 30 subjects, performed on two days; our dataset complements existing SSVEP datasets: i) multi-band SSVEP datasets are provided, and all three possible frequency bands (low, middle, and high) were used for SSVEP stimulation; ii) multi-day datasets are included; and iii) the EEG datasets include simultaneously obtained physiological measurements, such as respiration, electrocardiography, electromyography, head motion (accelerator), and body temperature.<br>Findings : To validate our dataset, we estimated the spectral powers and classification performance for the EEG (SSVEP) datasets, and created an example plot to visualize the physiological time-series data. Strong SSVEP responses were observed at stimulation frequencies, and the mean classification performance of the middle frequency band was significantly higher than the low- and high-frequency bands. Other physiological data also showed reasonable results.<br>Conclusions : Our multi-band, multi-day SSVEP datasets can be used to optimize stimulation frequencies because they enable simultaneous investigation of the characteristics of the SSVEPs evoked in each of the three frequency bands, and solve session-to-session (day-to-day) transfer problems by enabling investigation of the non-stationarity of SSVEPs measured on different days. Additionally, auxiliary physiological data can be used to explore the relationship between SSVEP characteristics and physiological conditions, providing useful information for optimizing experimental paradigms to achieve high performance.
**Corresponding Author:**	Han-Jeong Hwang<br>Kumoh National Institute of Technology<br>Gumi, Gyeongsangbuk-do KOREA, REPUBLIC OF
**Corresponding Author Secondary Information:**	
**Corresponding Author's Institution:**	Kumoh National Institute of Technology
**Corresponding Author's Secondary Institution:**	
**First Author:**	Ga-Young Choi
**First Author Secondary Information:**	
**Order of Authors:**	Ga-Young Choi
	Chang-Hee Han
	Young-Jin Jung
	Han-Jeong Hwang
**Order of Authors Secondary Information:**	

Powered by Editorial Manager® and ProduXion Manager® from Aries Systems Corporation

Response to Reviewers:	Please see the review note. Thanks!
Additional Information:	
Question	Response
Are you submitting this manuscript to a special series or article collection?	No
**Experimental design and statistics**<br><br>Full details of the experimental design and statistical methods used should be given in the Methods section, as detailed in our Minimum Standards Reporting Checklist. Information essential to interpreting the data presented should be made available in the figure legends.<br><br>Have you included all the information requested in your manuscript?	Yes
**Resources**<br><br>A description of all resources used, including antibodies, cell lines, animals and software tools, with enough information to allow them to be uniquely identified, should be included in the Methods section. Authors are strongly encouraged to cite Research Resource Identifiers (RRIDs) for antibodies, model organisms and tools, where possible.<br><br>Have you included the information requested as detailed in our Minimum Standards Reporting Checklist?	Yes
**Availability of data and materials**<br><br>All datasets and code on which the conclusions of the paper rely must be either included in your submission or deposited in publicly available repositories (where available and ethically appropriate), referencing such data using a unique identifier in the references and in the “Availability of Data and Materials” section of your manuscript.	No

Powered by Editorial Manager® and ProduXion Manager® from Aries Systems Corporation

Have you have met the above requirement as detailed in our Minimum Standards Reporting Checklist?	
If not, please give reasons for any omissions below.<br><br>as follow-up to "**Availability of data and materials**<br><br>All datasets and code on which the conclusions of the paper rely must be either included in your submission or deposited in publicly available repositories (where available and ethically appropriate), referencing such data using a unique identifier in the references and in the “Availability of Data and Materials” section of your manuscript.<br><br>Have you have met the above requirement as detailed in our Minimum Standards Reporting Checklist?<br><br>"	As far as I know, we can make our data open to reviewers after an initial check. Thus, I will provide our data after an initial check.

Manuscript with DOI
Click here to view linked References
Click here to access/download;Manuscript;revised_manuscript with DOI.docx

A multi-day and multi-band dataset for a steady-state visual-evoked potential-based brain-computer interface

Ga-Young Choi[1], Chang-Hee Han[2], Young-Jin Jung[3], Han-Jeong Hwang[1,*]

E-mail: cgy326@naver.com, zeros8706@naver.com, microbme@outlook.com, h2j@ kumoh.ac.kr

[1]Department of Medical IT Convergence Engineering, Kumoh National Institute of Technology, Gumi 39177, Republic of Korea

[2]Machine Learning Group, Berlin Institute of Technology (TU Berlin), 10623 Berlin, Germany

[3]Department of Radiological Science, Dongseo University, Busan 47011, Republic of Korea

Number of Pages: 30

Number of Figures: 8

Number of Tables: 2

Corresponding Author Information:

Name: Han-Jeong Hwang

Address: Kumoh National Institute of Technology, 350-27, Gumi-si, Gyeongsangbuk-do, Republic of Korea

Tel.: +82-054-478-7783

E-mail: h2j@kumoh.ac.kr

Abstract

Background: A steady-state visual-evoked potential (SSVEP) is a brain response to visual stimuli modulated at certain frequencies; it has been widely used in electroencephalography (EEG)-based brain–computer interface (BCI) research. However, there are few published SSVEP datasets for BCIs. In this study, we obtained a new SSVEP dataset based on measurements from 30 subjects, performed on two days; our dataset complements existing SSVEP datasets: i) multi-band SSVEP datasets are provided, and all three possible frequency bands (low, middle, and high) were used for SSVEP stimulation; ii) multi-day datasets are included; and iii) the EEG datasets include simultaneously obtained physiological measurements, such as respiration, electrocardiography, electromyography, head motion (accelerator), and body temperature.

Findings: To validate our dataset, we estimated the spectral powers and classification performance for the EEG (SSVEP) datasets, and created an example plot to visualize the physiological time-series data. Strong SSVEP responses were observed at stimulation frequencies, and the mean classification performance of the middle frequency band was significantly higher than the low- and high-frequency bands. Other physiological data also showed reasonable results.

Conclusions: Our multi-band, multi-day SSVEP datasets can be used to optimize stimulation frequencies because they enable simultaneous investigation of the characteristics of the SSVEPs evoked in each of the three frequency bands, and solve session-to-session (day-to-day) transfer problems by enabling investigation of the non-stationarity of SSVEPs measured on different days. Additionally, auxiliary physiological data can be used to explore the relationship between SSVEP characteristics and physiological conditions, providing useful information for optimizing experimental paradigms to achieve high performance.

Key words: steady-state visual-evoked potential (SSVEP); brain-computer interface (BCI); electroencephalography (EEG); physiological data

Data Description

Background and purpose

A brain–computer interface (BCI) is a non-muscular communication method that utilizes brain activity, such as the electroencephalogram (EEG), to assist handicapped individuals who are unable to voluntarily control their bodies [1, 2]. Two approaches have been employed to develop EEG-based BCIs; the difference between these two approaches is the presence of external stimuli [3]. Endogenous BCIs use mental imagery tasks to induce certain brain patterns, whereas exogenous BCIs use external stimuli to evoke certain brain patterns.

A representative endogenous BCI paradigm is motor imagery, which is defined as the mental simulation of motor behaviors, e.g., left/right hand movement [4, 5]. Owing to the event-related (de)synchronization phenomenon, different motor imagery tasks can be discriminated by using machine learning techniques; the discrimination results can then be used for BCI applications [6, 7]. To date, a large number of motor imagery BCI datasets have been published [8-14], and they have significantly contributed to the advancement of BCI research. Other endogenous types of BCI datasets are also available, such as slow cortical potential, readiness potential [8], and mental arithmetic datasets [13, 14].

There are two representative exogenous BCI paradigms: event-related potentials (ERPs) and steady-state visual-evoked potentials (SSVEPs). An ERP is a time-locked brain response that is evoked in response to specific visual, auditory, and/or tactile stimuli, whereas an SSVEP is a period brain response to a visual stimulus modulated at a certain frequency. ERPs have mostly been used in the development of row/column matrix spellers [15], whereas SSVEPs have been used in the development of a variety of BCI applications, such as robotic

arm control [16], exoskeletons [17], and functional electrical stimulation [18], and word spellers [19, 20]. Many ERP BCI datasets have become publicly available since the first ERP BCI dataset was published in 2003 [8]. However, it was not until 2017 that a freely accessible SSVEP BCI dataset was published for the first time [21]; it was followed by the second dataset in 2019, although the SSVEP paradigm has been widely used in BCI applications because high performance can be achieved with minimal training [22].

Because the number of SSVEP BCI datasets is small compared with the number of datasets based on other BCI paradigms, it would be beneficial for BCI researchers to provide a new SSVEP BCI dataset that can complement the existing SSVEP BCI datasets. The first SSVEP dataset was created from the data of 35 subjects who used a 40-target BCI speller; the SSVEP stimulation frequencies ranged from 8 to 15.8 Hz, with a span of 0.2 Hz [21]. The second SSVEP dataset was acquired based on the data from 54 subjects who used a 4-class BCI system over two sessions; 5.45, 6.67, 8.57, and 12 Hz were used as stimulation frequencies [22].

In this study, we created a new SSVEP BCI dataset that can contribute to SSVEP-based BCI research in three ways. First, our SSVEP dataset consists of three sub-datasets, each with a different frequency band: low (1–12 Hz), middle (12–30 Hz), and high (30–60 Hz) frequency bands. It is well documented that SSVEPs are elicited over a wide range of frequencies, from 1 to 90 Hz [23], and that the frequencies can be divided into three sub-frequency bands (i.e., low, middle, and high), as mentioned above [24]. The two previous SSVEP datasets were acquired by applying stimulation frequencies in certain frequency bands, i.e., 8–15.8 Hz in the low and middle frequency bands [21], and 5.45–12 Hz in the low frequency band [22]. Considering that the choice of the stimulation frequency band is an

important factor that significantly affects SSVEP-based BCI performance [25], the characteristics of the SSVEPs evoked in each of the three frequency bands should be investigated in coincidence with the corresponding signal-to-noise ratio (SNR) and classification performance. In particular, the high frequency band is currently receiving increasing attention as an alternative to the low and middle frequency bands, despite it being associated with relatively low performance, because it results in less visual fatigue [26]. However, no SSVEP BCI studies have provided available datasets for the high frequency band. Thus, it is necessary to have access to an SSVEP dataset that includes high frequency band data, in addition to low and middle frequency band data, to investigate the above-mentioned peculiarities. Our SSVEP dataset satisfies this requirement because it includes data for each of the three frequency bands that were independently acquired from the same subjects. Secondly, we provide a multi-session (multi-day) dataset that was recorded over two different days from the same subjects. Thus, our SSVEP dataset can be used to study session-to-session transfer, which is a challenging problem in BCI research [27-29]. A multi-session SSVEP dataset was also provided in [22]; however, it was acquired on the same day, with only a short break (i.e., 3 min.). Therefore, our dataset can offer more profound insight into the non-stationary nature of EEG signals, and thereby provide useful solutions to session-to-session (day-to-day) transfer problems. Lastly, we provide other physiological data for the dataset, i.e., data that have not been included in the two previously published SSVEP datasets [22, 23], in addition to the EEG dataset, to evaluate changes to the physiological condition of subjects during the experiment, such as respiration, electrocardiography (ECG), neck electromyography (EMG), head motion, and body temperature. The auxiliary physiological data can be used to explore the relationships between SSVEP characteristics (e.g., SNR) and various physiological variables, thereby providing information that can be used to design

experimental paradigms to achieve high performance.

To create a novel SSVEP BCI dataset that is complementary to the two currently available SSVEP BCI datasets, we designed a 4-class SSVEP paradigm that is similar to that used to acquire the second SSVEP BCI dataset [22]. Three sets of four stimulation frequencies were employed for the low, middle, and high frequency bands, respectively. The SSVEP BCI dataset was created using data that were collected from 30 subjects on two different days. For data validation, we applied a standard analysis method to our SSVEP dataset, and analyzed the baseline results in consideration of all of the above-mentioned physiological data that were obtained in this study.

Experimental design

Subject

A total of 30 subjects (9 females and 21 males; 23.8 ± 1.3 years) were recruited for this study. The number of subjects was decided to be 30 because a sample size of 30 is sufficient to apply parametric statistical tests for analysis of the results. Note that parametric statistical tests provide more statistical power than non-parametric ones, and thereby ensure more reliable validation of our SSVEP dataset. No subject had any history of psychiatric disease that could have affected the research results. Seven of the 30 subjects had prior BCI experience, but they participated in endogenous BCI experiments that required them to perform a mental arithmetic task. Thus, it was assumed that their prior BCI experience would not significantly affect the research results. Before the experiment, they were given the details of the experimental procedures, and signed a form providing informed consent for study

participation and the anonymous release of their data to the public. Adequate reimbursement was provided for their participation after the experiment. This study was approved by the Institutional Review Board (IRB) of Kumoh National Institute of Technology (No. 6250) and was conducted in accordance with the principles of the declaration of Helsinki.

Stimulator

The SSVEP stimulator was made of two square pieces of styrofoam, a sheet of thick black paper, an opaque film, four LEDs, and an LED controller. We first cut one of the styrofoam pieces to make five sections, four of which were 3 cm × 3 cm and purposed for the LED display, and the other was 9 cm × 5.5 cm and purposed to show instructions during the experiment (Figure 1(a)). After that, we inserted four LEDs into the four square holes that were punctured through the 3 cm × 3 cm sections (part number: T03WC01; operating current: 20 mA; viewing angle: $\theta/2 = 100°$; luminous intensity: 2,000 mcd; emitting color: white), and attached another styrofoam piece to the back of the sectioned styrofoam. The front part of the stimulator was covered with an opaque film to diffuse the light, and then we attached a piece of black paper with five square holes, which were exactly matched to those punctured through the front styrofoam piece, to the opaque film for better visibility. The stimulator was attached to a 21-in. LCD monitor, and an instruction, i.e., on which LED the subject should focus, was presented with an arrow from the monitor through the center square hole of the 9 cm × 5.5 cm styrofoam piece. A schematic diagram of the SSVEP stimulator is shown in Figure 1(a). The distance between each LED and instruction arrow presented at the center of the monitor was 17 cm. To control the stimulator, we used a LAUNCHXL-F28027 Board powered by C2000 MCU (Texas Instruments). The duty cycle was set at 50%, meaning that the LED had 50% on-time and 50% off-time.

As mentioned above, three different frequency bands (low: 1–12 Hz, middle: 12–30 Hz, and high: 30–60 Hz [30]) were individually applied for SSVEP stimulation to obtain multi-band SSVEP datasets in this study. Three sets of four stimulation frequencies were implemented for each frequency band, as follows: 5, 5.5, 6, and 6.5 Hz for the low frequency band; 21, 21.5, 22, and 22.5 Hz for the middle frequency band; and 40, 40.5, 41, and 41.5 Hz for the high frequency band. The four stimulation frequencies for each frequency band were selected such that the harmonic frequencies of the four frequencies in the low frequency band would not overlap with any of the four frequencies in the middle or high frequency bands, and the harmonic frequencies of the four frequencies in the middle frequency band would not overlap with the four frequencies in the high frequency band. This was done because simultaneous implementation of the harmonic frequencies as different stimulation frequencies can significantly decrease the performance of SSVEP-based BCI systems [31]. Additionally, the alpha frequency band was not considered because its use can produce a considerable number of false positives [30, 32], even though employing the alpha band for SSVEP stimulation tends to yield a high SNR. We assigned four stimulation frequencies to four LEDs, depending on the stimulation frequency band, as shown in figure 1(b).

[Figure 1 here]

Experimental paradigm

During the experiment, the subjects sat in a comfortable arm chair that was distanced 1 m from the SSVEP stimulator, which was attached to a 21-in. monitor, and they were instructed to remain relaxed without any movement. Note that all instructions were presented at the center of the monitor, and the subjects could view them through the center hole of the stimulator. For each trial, a blank screen was presented for 5 s, and then an arrow indicating

one of the four LEDs was presented for 6 s; during this time, the subject was asked to gaze at the target LED, as instructed by the direction of an arrow. Subsequently, a white cross mark (‘+’) was presented for 6 s to indicate a short break before the next trial. A short beep sound was also presented with every visual stimulus transition in order to explicitly capture the attention of the subjects. The direction of each arrow was randomly presented 20 times (20 trials) for each direction, resulting in a total of 80 trials; this was repeated for each frequency band (i.e., low, middle, and high). To prevent excessive fatigue, a minimum 5-min break was allotted to each subject after every 40 trials (40 trials equate to one session); irregular breaks were also allowed as requested by the subjects during the experiment. Each subject performed six sessions of the SSVEP experiment (i.e., two sessions × three frequency bands) twice on different days, with an interval of at least one day. The order of the stimulation frequency band trials was varied for counterbalancing between subjects. In particular, all possible order combinations of the three frequency bands were as follows: (low–middle–high), (low–high–middle), (middle–low–high), (middle–high–low), (high–low–middle), and (high–middle–low). Each order combination was randomly assigned to five subjects (6 combinations × 5 subjects = 30 subjects), and the same order was used on both days once it was assigned to the subject on the first day of the experiment. The entire experiment lasted approximately 2 h each day, including the time for EEG preparation.

Data recording

The EEG signals were measured by using a BrainAmp EEG amplifier (Brain products, GmbH Ltd., Germany) with a sampling rate of 1,000 Hz; the ground and reference electrodes were respectively attached at Fpz and FCz sites (Figure 2). We used 33 active

electrodes, which were mounted according to the International 10-10 system, to measure EEG signals (FP1, FP2, AF4, AF3, F5, Fz, FC1, FC5, F6, FC2, FC6, C4, Cz, C3, CP1, CP2, CP6, P8, P4, Pz, POz, PO4, PO8, O2, Oz, O1, PO3, P3, CP5, P7, PO7, T7, and T8); electrodes were more densely mounted around occipital areas, relative to other areas, because the SSVEPs mainly originated from the occipital lobe. We did not control for changes to electrode locations between the two days; we instead tried to maintain the conditions of EEG measurement between the two days for each subject. This is because slight changes to electrode locations are inevitable, as it would happen with daily BCI use; thus, our dataset can effectively address session-to-session (day-to-day) transfer problems. Note that electrode location change between days is an important factor in EEG non-stationarity between days [33].

[Figure 2 here]

We also measured various physiological signals as the EEG signals were measured, i.e., respiration, ECG, neck EMG, head motion, and body temperature, to investigate physiological changes. To measure these physiological signals, we attached a respiratory belt to the chest, three ECG sensors on lead-I position (Einthoven's triangle), two EMG sensors on the right and left sides of the neck, an inertial measurement unit (IMU) sensor on the top of the head between Cz and CPz, and a temperature sensor in the armpit. The same amplifier that was used for measuring EEG signals was used to record the physiological signals at the same sampling rate of 1,000 Hz; thus, all of the measured data were synchronized. The physiological data can be used to investigate the relationships between changes in brain activity and various physiological variables, as well as to develop artifact correction algorithms. For example, some researchers previously simultaneously used EEG and ECG to

evaluate the psychological state and stress level/mental effort of subjects [34, 35], whereas others used motion data to remove motion-related artifacts from EEG data [36, 37].

Data format and structure

Because data analysis was performed using Matlab R2013b (MathWorks, Natick, MA, USA), we provide our dataset in the form of Matlab files (.mat). Each data folder has two sub-folders, each containing a sub-dataset corresponding to one of the two experimental days (i.e., day1 and day2). Each sub-folder has *cnt* and *mrk* files, which contain continuous time-series data for all physiological measurements (*cnt*) and the corresponding data with the trigger information (*mrk*), respectively. The *cnt* and *mrk* files have suffixes corresponding to three frequency bands and session numbers. For example, *cnt*_Low(1) denotes time-series data that were obtained by using the low frequency band for SSVEP stimulation in the first session. Thus, the sub-folder for each subject contains the following six pairs of *cnt* and *mrk* files: *cnt*_Low(1), *mrk*_Low(1), *cnt*_Low(2), *mrk*_Low(2), *cnt*_Middle(1), *mrk*_Middle(1), *cnt*_Middle(2), *mrk*_Middle(2), *cnt*_High(1), *mrk*_High(1), *cnt*_High(2), and *mrk*_High (2). All data were down-sampled to 200 Hz when the raw data were converted to Matlab-compatible files. Table 1 lists all of the data files provided for each sub-folder.

Table 1. Data format

**Frequency Band**	**Stimulation Frequency (Hz)**	**Data format (*.mat)**
**Low**	5	*cnt*_Low(1), *cnt*_Low(2)
	5.5	*mrk*_Low(1), *mrk*_Low(2)
	6	

	6.5	
**Middle**	21	
	21.5	*cnt*_Middle(1), *cnt*_Middle(2)
	22	*mrk*_Middle(1), *mrk*_Middle(2)
	22.5	
**High**	40	
	40.5	*cnt*_High(1), *cnt*_High(2)
	41	*mrk*_High(1), *mrk*_High(2)
	41.5	

Each data folder for each subject has two sub-folders that contain two sub-datasets that correspond to measurements taken on two different days, and each sub-folder has six pairs of *cnt* and *mrk* files (shown below) that have been labeled according to the stimulation frequency band and session number.

Questionnaire

We asked subjects to fill out two different questionnaires before and after the experiment. Table 2 presents two sets of questionnaires. Seven (A1 to A7) and three (B1 to B3) questions were asked before the experiment to record the demographics and initial physical condition of the subject, and after the experiment to check the physical condition of the subject (i.e., the level of drowsiness, concentration, and eye strain), respectively. The answers to the questionnaires have been provided in a supplementary file (questionnaires_answers.xlsx). Note that, because all subjects were university students in their twenties who did not take any medication or drink alcohol 24 h before the experiment, we did not include the related information (i.e., A-2: Age Group, A-5: Drinking Alcohol, and A-7: Medicine) in the

supplementary file.

Table 2. Two sets of questionnaires answered before and after the experiment.

Before Experiment		
**NUMBER**	**QUESTIONNAIRE**	**ANSWER**
A–1	Gender	Male = 1/ Female = 2
A–2	Age Group	10s = 1/ 20s = 2/ 30s = 3/ >40s = 4
A–3	Job	Middle/high school student = 1/ Undergraduate = 2/ Postgraduate = 3/ Others = 4
A–4	Sleeping Hours	Less than 5 h = 1/ 6 h =2/ 7 h = 3/ 8 h = 4/ >9 h = 5
A–5	Alcohol Consumption	No = 1/ Yes = 2
A–6	Overall Body Condition	(Good) 1 2 3 4 5 6 7 8 9 10 (Bad)
A–7	Medicine	No = 1/ Yes = 2
**After Experiment**		
**NUMBER**	**QUESTIONNAIRE**	**ANSWER**
B–1	Drowsiness	(Good) 1 2 3 4 5 6 7 8 9 10 (Bad)
B–2	Concentration	(Bad) 1 2 3 4 5 6 7 8 9 10 (Good)
B–3	Eye Strain	(Good) 1 2 3 4 5 6 7 8 9 10 (Bad)

Data Validation

Methods

Because our main concern was the EEG dataset measured during the SSVEP experiment, we provide detailed results of analysis for the EEG dataset, and example time-series data for the other physiological datasets.

The EEG data were first band-pass-filtered with different cutoff frequencies according to the stimulation frequency band, as follows: 3–9, 18–24, and 38–44 Hz for the low, middle, and

high frequency bands, respectively. From the band-pass filtered data, we extracted 6-s epochs that were measured while the subjects were focusing on each of the target LEDs, and used them for further analysis. To visualize the SSVEP responses, spectral powers were estimated for each channel by applying a moving-window technique (2.5-s window size with 90% overlap). The SSVEP SNR was also calculated by dividing the SSVEP amplitude at the stimulation frequency by the mean spectral amplitude of six adjacent frequencies to demonstrate the reliability of our SSVEP dataset [38].

$$\mathrm{SNR} = \frac{n \times y(f)}{\sum_{k=1}^{n/2} [y(f+0.5 \times k)+y(f-0.5 \times k)]} \quad (1)$$

where *n* is the number of adjacent points (six in this study), *y* is the spectral amplitude, and *f* is the stimulation frequency. Canonical correlation analysis (CCA), which is the most widely used method for classifying SSVEP data, was used for 4-class classification [39].

Each of the six types of physiological data was linearly detrended to remove baseline drift. The respiratory rate and heart rate were respectively estimated using the respiration and ECG data based on the peak information for each frequency band and each session to evaluate the ranges of the respiratory and heart rates. The mean and standard deviation values were estimated for each trial for the other types of physiological signals (i.e., EMG1, EMG2, IMU, and body temperature) to evaluate changes in each set of physiological data.

Results

Figure 3 shows topographic maps corresponding to the SSVEP frequencies, as averaged using the data collected over two days for all subjects and the four stimulation frequencies in each frequency band. As expected, strong SSVEPs were observed near occipital areas in all

cases. High spectral powers were also observed near fronto-temporal areas, which would be derived from electrooculography (EOG). As is well known, absolute spectral powers decrease from the low frequency band to the high frequency band (see the color bar range in Figure 3). The occipital SSVEPs were high relative to those observed in the other brain areas when the middle frequency band was applied; a spatially high SSVEP SNR was observed.

[Figure 3 here]

Figure 4 shows SSVEP SNR topographic maps that were averaged using the single-day data for the four stimulation frequencies of each frequency band for all subjects. Most channels achieved SSVEP SNRs that were higher than 1 for all stimulation frequencies, with parieto-occipital channels achieving high SSVEP SNRs that exceed 2, demonstrating the reliability of our SSVEP datasets. Additionally, the Day-1 and Day-2 SSVEP topographic maps appear to be very similar, corresponding to a high cross-correlation ($r > 0.99$) for all comparison cases. This result demonstrates a small discrepancy between the electrode locations on the first and second days. All SSVEP SNRs are provided with 12 supplementary files (4 stimulation frequencies × 3 frequency bands) for each day, and each supplementary file contains the SSVEP SNR data for each channel and trial for all subjects. The cross-correlation analysis results for each subject are also provided for the four stimulation frequencies in each frequency band with a supplementary file (SNR_CrossCorrelation.xlsx).

[Figure 4 here]

Figure 5 shows the grand-average spectral powers, as estimated by using the EEG data measured from 13 parieto-occipital channels (Ch_Set4) during visual stimulation for the four stimulation frequencies in the three frequency bands. Spectral peaks can be observed at the

stimulation frequencies, regardless of the frequency band. Note that, among the 60 sub-datasets (30 subjects × 2 d), 10 datasets were excluded for this analysis because these datasets contained data showing extremely large SSVEP amplitudes at non-stimulation frequencies for some trials, and thus distorted the grand-average results (excluded datasets: Day 1 and 2 for S2; Day 2 for S10; Day1 and 2 for S11; Day2 for S13; Day1 for S18; Day1 for S20; Day1 and 2 for S29).

[Figure 5 here]

The classification accuracy results are presented for each stimulation frequency band in Figure 6 with respect to the channel configuration shown in Figure 2. The classification accuracy gradually increased as the number of channels employed for classification was reduced to eight channels in frontal areas (Ch_Set5), regardless of the frequency band; this means that occipital areas are most associated with visual information processing, and thus provide the most discriminative information. However, the classification performance considerably deteriorated when only three electrodes (Ch_Set6: O1, O2, and Oz) were attached above occipital areas, because less information was obtained.

[Figure 6 here]

Figure 7 shows the mean classification accuracies for each frequency band on each experimental day; the results were obtained by using the best channel configuration (Ch_Set5) in terms of classification accuracy, as shown in Figure 6. A similar statistical trend is shown for each experimental day; the mean classification accuracy for the middle frequency band was significantly higher than those for the low and high frequency bands, and the mean classification accuracy for the low frequency band was only found to be higher than

that for the high frequency band on the second day (RM-ANOVA: $F(2, 29) = 19.87$, $p < 0.01$; paired t-test Bonferroni corrected $p < 0.05$: middle > low = high on the first day; RM-ANOVA: $F(2, 29) = 23.09$, $p < 0.01$; paired t-test Bonferroni corrected $p < 0.05$: middle > low > high on the second day). No significant difference was observed between the two days with respect to the stimulation frequency band.

[Figure 7 here]

Examples of the six types of physiological signals that were measured along with the EEG signals are presented in Figure 8. Because the physiological data show high inter- and intra-subject variability, representative examples are provided for each of the six types of physiological data; detailed results are provided as six supplementary figures, and in 12 supplementary files. The example data were measured from S2 during their first trial, when the subject started to focus on an LED that was modulated at 5 Hz; the duration was 60 s. In particular, 13 breaths and 93 heartbeats were clearly observed over the 60-s period in the respiratory (Figure 8(a)) and ECG data (Figure 8(b)), respectively; these numbers are within the normal ranges for the adult respiratory rate (12–18) [40] and heart rate (60–100) [41]. The two sets of example EMG data (Figures 8(c) and (d)) and example head motion (Figure 8(e)) data show that no significant movement was made; heartbeats were also observed in both sets of EMG data (Figures 8(c) and (d)). Most subjects showed similar trends for each corresponding type of physiological signal, with the exception of a few cases (see supplementary figures and files).

[Figure 8 here]

Re-use potential

Although the SSVEP is one of the most widely used BCI paradigms [42], publicly available SSVEP BCI datasets are still scarce. In this study, we created multi-band and multi-day SSVEP BCI datasets for the first time and validated their feasibility through SSVEP spectral power and classification analyses. All of the results were found to be consistent with those reported in previous studies; particularly, SSVEP responses were mainly observed near occipital areas, with spectral peaks occurring at the stimulation frequencies regardless of the stimulation frequency band; additionally, the classification accuracy for the middle frequency band was higher than those for the low and high frequency band [25, 43]. Our multi-band SSVEP datasets can be used to investigate subject-specific stimulation frequencies because they enable comparison of the characteristics of the SSVEPs evoked in each of the three frequency bands, which can thus be used to improve the performance of SSVEP-based BCIs. Additionally, the multi-day SSVEP datasets can be used to develop advanced solutions for session-to-session (day-to-day) transfer problems because they provide data that can be used to investigate how SSVEP characteristics can differ on different days, the analysis of which can be used to enhance the reliability of SSVEP-based BCIs.

All other physiological signals that were simultaneously measured with the EEG signals also yielded reasonable results, even though only a representative example of each type of signal result was shown because there was high inter- and intra-subject variability. The physiological data can be used not only to investigate the relationship between brain activity and various physiological variables, but also to develop artifact correction methods for SSVEPs. Particularly for the latter case, IMU and EMG data can be used to detect head/neck

movements that would degrade the quality of EEG data, and then to correct them based on advanced algorithms.

Availability requirements

It will be filled out later after an initial editorial assessment for scope and scale.

Availability of supporting data

The data supporting this paper, including the EEG and other physiological datasets, and the questionnaire results, are available in the *GigaScience* database, GigaDB [44].

Declarations

List of Abbreviations

SSVEP: steady-state visual-evoked potential; BCI: brain-computer interface; ERP: event-related potential; SNR: signal-to-noise ratio; CCA: canonical correlation analysis; electromyography: EMG; electrocardiography: ECG; inertial measurement unit: IMU; electrooculography: EOG.

Ethical Approval

This study was approved by the Institutional Review Board (IRB) of Kumoh National Institute of Technology (No. 6250).

Competing interests

The authors declare that they have no competing interests.

Funding

This work was supported by the Institute for Information & Communications Technology Planning & Evaluation (IITP) grant funded by the Korea government (No. 2017-0-00451; Development of BCI based Brain and Cognitive Computing Technology for Recognizing User's Intentions using Deep Learning).

Authors' contributions

G.-Y.C., and H.-J.H., designed the experiment, Y.-J.J., implemented an SSVEP stimulator, G.-Y.C., acquired the data, and G.-Y.C., C.-H.H., Y.-J.J., performed data analysis, and H.-J.H., supervised this study. All authors wrote and reviewed the manuscript.

References

1. Pfurtscheller G, Flotzinger D and Kalcher J. Brain-computer interface-a new communication device for handicapped persons. J Microcinoyt Appl. 1993;16(3):293-9.

2. Wolpaw JR, Birbaumer N, McFarland DJ, Pfurtscheller G and Vaughan TM. Brain-computer interfaces for communication and control. Clin Neurophysiol. 2002;113(6):767-91.

3. Nicolas-Alonso LF and Gomez-Gil J. Brain computer interfaces, a review. Sensors. 2012;12(2):1211-79.

4. Decety J and Ingvar DH. Brain structures participating in mental simulation of motor behavior: A neuropsychological interpretation. Acta psychol. 1990;73(1):13-34.

5. Jeannerod M and Frak V. Mental imaging of motor activity in humans. Curr Opin Neurobiol. 1999;9(6):735-9.

6. Pfurtscheller G, Brunner C, Schlögl A and Da Silva FL. Mu rhythm (de) synchronization and EEG single-trial classification of different motor imagery tasks. NeuroImage. 2006;31(1):153-9.

7. Pfurtscheller G and Neuper C. Motor imagery and direct brain-computer communication. Proc IEEE. 2001;89(7):1123-34.

8. Blankertz B, Muller K-R, Curio G, Vaughan TM, Schalk G, Wolpaw JR, et al. The BCI competition 2003: progress and perspectives in detection and discrimination of EEG single trials. IEEE Trans Biomed Eng. 2004;51(6):1044-51.

9. Blankertz B, Muller K-R, Krusienski DJ, Schalk G, Wolpaw JR, Schlogl A, et al. The BCI competition III: Validating alternative approaches to actual BCI problems. IEEE Trans Neural Syst Rehabil Eng. 2006;14(2):153-9.

10. Cho H, Ahn M, Ahn S, Kwon M and Jun SC. EEG datasets for motor imagery brain computer interface. Gigascience. 2017.

11. Sajda P, Gerson A, Muller K-R, Blankertz B and Parra L. A data analysis competition to evaluate machine learning algorithms for use in brain-computer interfaces. IEEE Trans Neural Syst Rehabil Eng. 2003;11(2):184-5.

12. Tangermann M, Muller K-R, Aertsen A, Birbaumer N, Braun C, Brunner C, et al. Review of the BCI competition IV. Front Neurosci. 2012;6:55.

13. Shin J, von Luhmann A, Blankertz B, Kim D-W, Jeong J, Hwang H-J and Müller K-R. Open access dataset for EEG+ NIRS single-trial classification. IEEE Trans Neural Syst

Rehabil Eng. 2017;25(10):1735-45.

14. BNCI Horizon 2020 Datasets;. Accessed: 2019-03-25. http://bnci-horizon-2020.eu/database/data-sets.

15. Farwell LA and Donchin E. Talking off the top of your head: toward a mental prosthesis utilizing event-related brain potentials. Elecroen Clin Neuro. 1988;70(6):510-23.

16. Sakurada T, Kawase T, Takano K, Komatsu T and Kansaku K. A BMI-based occupational therapy assist suit: asynchronous control by SSVEP. Front Neurosci. 2013;7:172.

17. Kwak N-S, Müller K-R and Lee S-W. A lower limb exoskeleton control system based on steady state visual evoked potentials. J Neural Eng. 2015;12(5):056009.

18. Gollee H, Volosyak I, McLachlan AJ, Hunt KJ and Gräser A. An SSVEP-based brain-computer interface for the control of functional electrical stimulation. IEEE Trans Biomed Eng. 2010;57(8):1847-55.

19. Hwang H-J, Lim J-H, Jung Y-J, Choi H, Lee SW and Im C-H. Development of an SSVEP-based BCI spelling system adopting a QWERTY-style LED keyboard. J Neurosci Methods. 2012;208(1):59-65.

20. Lim J-H, Lee J-H, Hwang H-J, Kim DH and Im C-H. Development of a hybrid mental spelling system combining SSVEP-based brain-computer interface and webcam-based eye tracking. Biomed Signal Process Control. 2015;21:99-104.

21. Wang Y, Chen X, Gao X and Gao S. A benchmark dataset for SSVEP-based brain-computer interfaces. IEEE Trans Neural Syst Rehabil Eng. 2017;25(10):1746-52.

22. Lee M-H, Kwon O, Kim Y-J, Kim H-K, Lee Y-E, Williamson J, et al. EEG Dataset and OpenBMI Toolbox for Three BCI Paradigms: An Investigation into BCI Illiteracy. GigaScience. 2019.

23. Herrmann CS. Human EEG responses to 1-100 Hz flicker: resonance phenomena in visual cortex and their potential correlation to cognitive phenomena. Exp Brain Res. 2001;137(3-4):346-53.

24. Galloway N. Human brain electrophysiology: Evoked potentials and evoked magnetic fields in science and medicine. Br J Ophthalmol. 1990;74(4):255.

25. Volosyak I, Valbuena D, Luth T, Malechka T and Graser A. BCI demographics II: How many (and what kinds of) people can use a high-frequency SSVEP BCI? IEEE Trans Neural Syst Rehabil Eng. 2011;19(3):232-9.

26. Sakurada T, Kawase T, Komatsu T and Kansaku K. Use of high-frequency visual stimuli above the critical flicker frequency in a SSVEP-based BMI. Clin Neurophysiol. 2015;126(10):1972-8.
27. Cho H, Ahn M, Kim K and Jun SC. Increasing session-to-session transfer in a brain-computer interface with on-site background noise acquisition. J Neural Eng. 2015;12(6):066009
28. Krauledat M, Tangermann M, Blankertz B and Müller K-R. Towards zero training for brain-computer interfacing. PloS one. 2008;3(8):e2967.
29. Samek W, Meinecke FC and Müller K-R. Transferring subspaces between subjects in brain-computer interfacing. IEEE Trans Biomed Eng. 2013;60(8):2289-98.
30. Lin Z, Zhang C, Wu W and Gao X. Frequency recognition based on canonical correlation analysis for SSVEP-based BCIs. IEEE Trans Biomed Eng. 2006;53(12): 2610-2614.
31. Hwang H-J, Kim S, Choi S, Im C-H. EEG-based brain-computer interfaces: a thorough literature survey. Int J Hum-Comput Interact. 2013;29(12): 814-826.
32. Müller SMT, Diez PF, Bastos-Filho TF, Sarcinelli-Filho M, Mut V, Laciar E, and Avila E. Robotic wheelchair commanded by people with disabilities using low/high-frequency ssvep-based BCI. In World Congress on Medical Physics and Biomedical Engineering, 2015. p. 1177-1180.
30. Zhu D, Bieger J, Molina G G, and Aarts R M. A survey of stimulation methods used in SSVEP-based BCIs. Comput Intell Neurosci, 2010; 1.
31. Hwang H J, Kim D H, Han C H, and Im C H. A new dual-frequency stimulation method to increase the number of visual stimuli for multi-class SSVEP-based brain–computer interface (BCI). Brain Res, 2013; 1515: 66-77.
32. Cheng M, Gao X, Gao S, and Xu D. Design and implementation of a brain-computer interface with high transfer rates. IEEE Trans Biomed Eng. 2002; 49(10): 1181-1186.
33. Park S A, Hwang H J, Lim J H, Choi J H, Jung H K, and Im C H. Evaluation of feature extraction methods for EEG-based brain–computer interfaces in terms of robustness to slight changes in electrode locations. Med Biol Eng Comput, 2013;51(5):571-579.
34. Gonzalez-Franco M, Yuan P, Zhang D, Hong B, and Gao S. Motor imagery based brain-computer interface: A study of the effect of positive and negative feedback. In Proceedings of 2011 Annual International Conference of the IEEE Engineering in Medicine and Biology Society, 2011; 6323-6326.

35. Pfurtscheller G, Solis Escalante T, Barry R J, Klobassa D S, Neuper C, and Mueller-Putz, G. Brisk heart rate and EEG changes during execution and withholding of cue-paced foot motor imagery. Front Hum Neurosci. 2013; 7: 379.

36. Gwin J T, Gramann K, Makeig S, and Ferris D P. Removal of movement artifact from high-density EEG recorded during walking and running. J Neurophysiol. 2010; 103(6):3526-3534.

37. O'Regan S, Faul S, and Marnane W. Automatic detection of EEG artefacts arising from head movements using EEG and gyroscope signals. Med Eng Phys. 2013; 35(7):867-874.

38. Vialatte F-B, Maurice M, Dauwels J, Cichocki A. Steady state visual evoked potentials in the delta range (0.5–5 Hz). In Proceedings of 15th International Conference on Advances in Neuro-information Processing. 2009; 400–407.

39. Lin Z, Zhang C, Wu W and Gao X. Frequency recognition based on canonical correlation analysis for SSVEP-based BCIs. IEEE Trans Biomed Eng. 2006;53(12): 2610-2614.

40. Barrett K E, Barman S M, Boitano S and Brooks H. Ganong's review of medical physiology. NY: McGraw-Hill Medical. 2009. p. 23.

41. Aladin A I, Whelton S P, Al-Mallah M H, Blaha M J, Keteyian S J, Juraschek S P, et al. Relation of resting heart rate to risk for all-cause mortality by gender after considering exercise capacity (the Henry Ford exercise testing project). Am J Cardiol. 2014;114(11): 1701-1706.

42. Hwang H-J, Kim S, Choi S, Im C-H. EEG-based brain-computer interfaces: a thorough literature survey. Int J Hum-Comput Interact. 2013;29(12): 814-826.

43. Müller SMT, Diez PF, Bastos-Filho TF, Sarcinelli-Filho M, Mut V, Laciar E, and Avila E. Robotic wheelchair commanded by people with disabilities using low/high-frequency ssvep-based BCI. In World Congress on Medical Physics and Biomedical Engineering, 2015. p. 1177-1180.

44. Choi G-Y; Han C-H; Jung Y-J; Hwang H-J: Supporting data for "A multi-day and multi-band dataset for steady-state visual evoked potential-based brain-computer interface" GigaScience Database. 2019. http://dx.doi.org/10.5524/100660

Figures

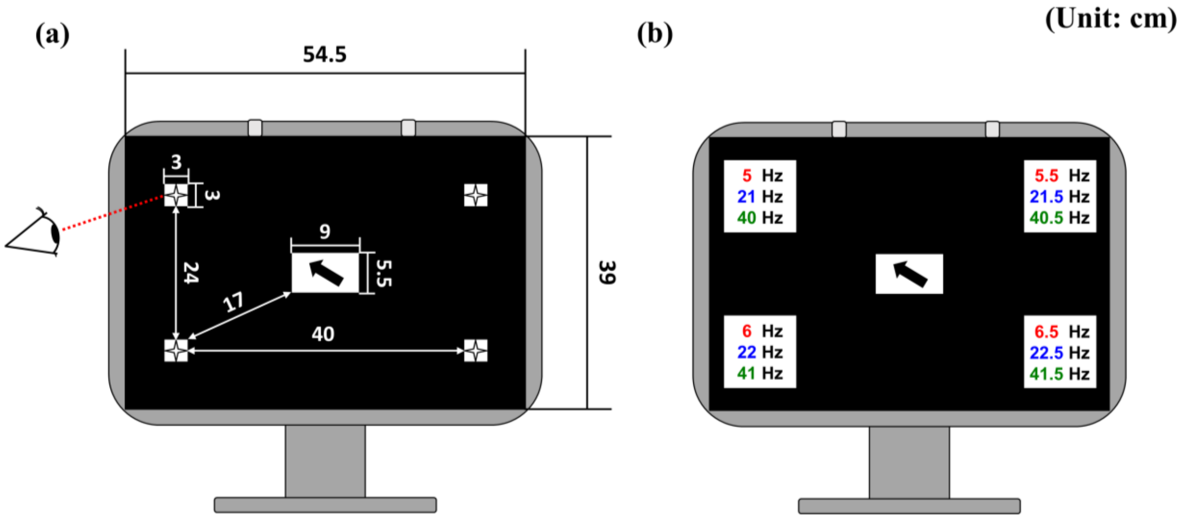

Figure 1. (a) Schematic diagram of the SSVEP stimulator (unit: cm). (b) Placement of the four stimulation frequencies for each of the three stimulation frequency bands (5, 5.5, 6, and 6.5 Hz for the low frequency band; 21, 21.5, 22, and 22.5 Hz for the middle frequency band; 40, 40.5, 41, and 41.5 Hz for the high frequency band).

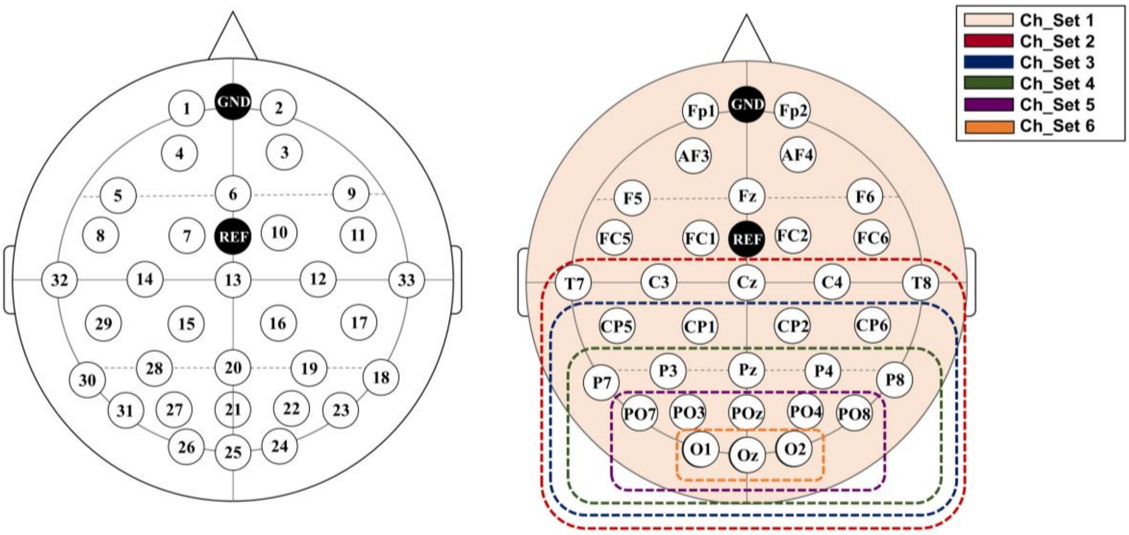

Figure 2. Electrode positions used in the experiment with respect to the (a) number and (b) position name. Note that six different channel sets were used for data analysis to evaluate the impact of the number of electrodes on classification performance.

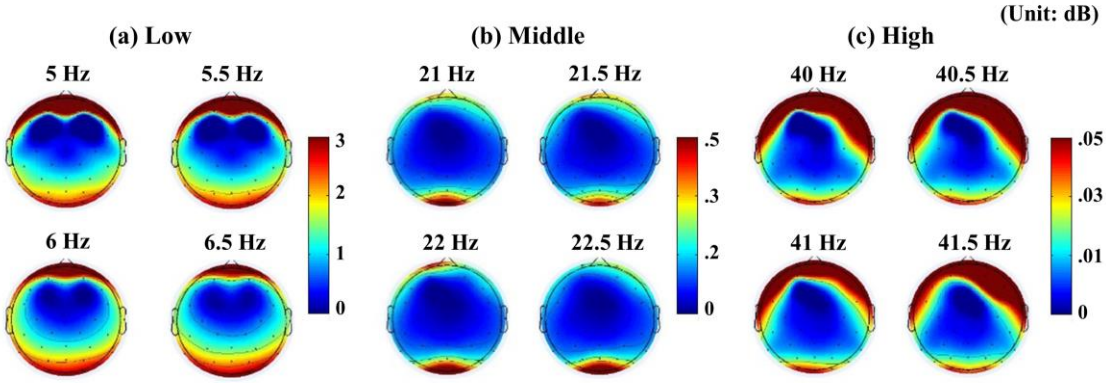

Figure 3. Topographic maps at the SSVEP frequencies averaged over two days with all subjects for the four stimulation frequencies of (a) the low, (b) middle, and (c) high frequency band.

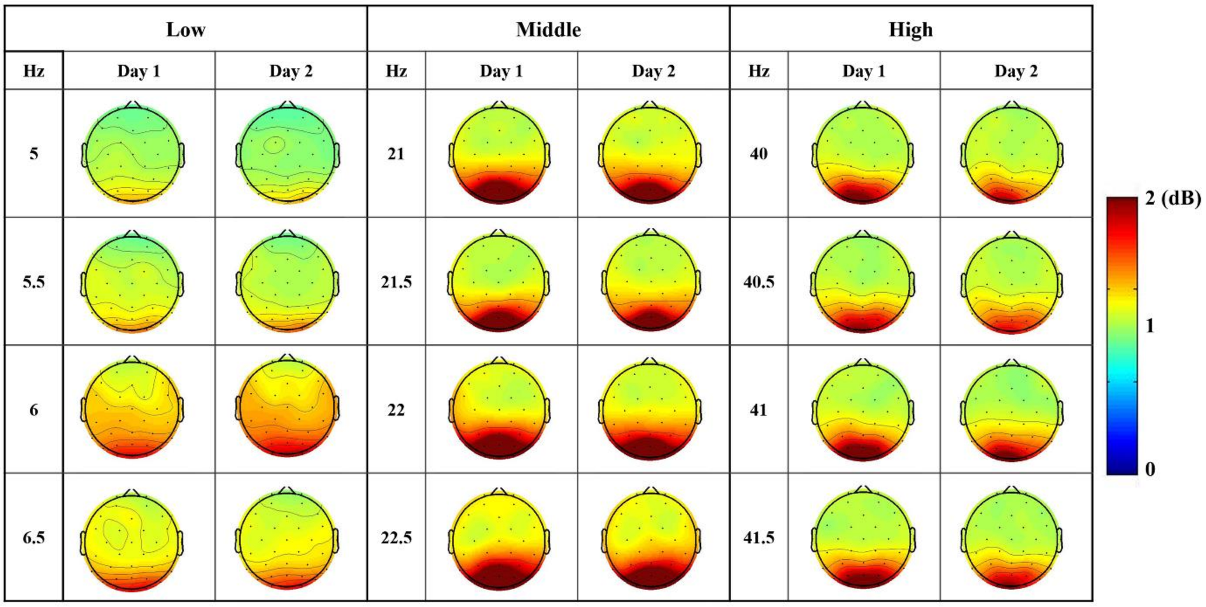

Figure 4. SSVEP SNR topographic maps averaged for each day with all subjects for the four stimulation frequencies of the low, middle, and high frequency band, respectively. The cross-correlation coefficients are higher than 0.99 for all cases.

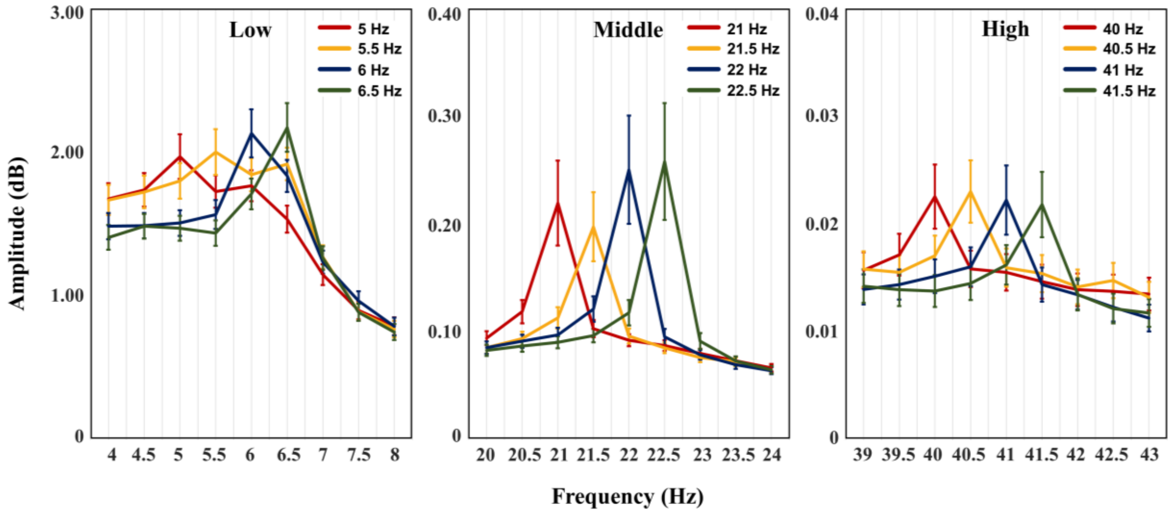

Figure 5. Grand-average SSVEP responses estimated based on the data from 13 parieto-occipital channels (Ch_Set4) for each frequency band. Spectral peaks are observed at each stimulation frequency. The vertical bars indicate the standard errors of the spectral powers for each frequency.

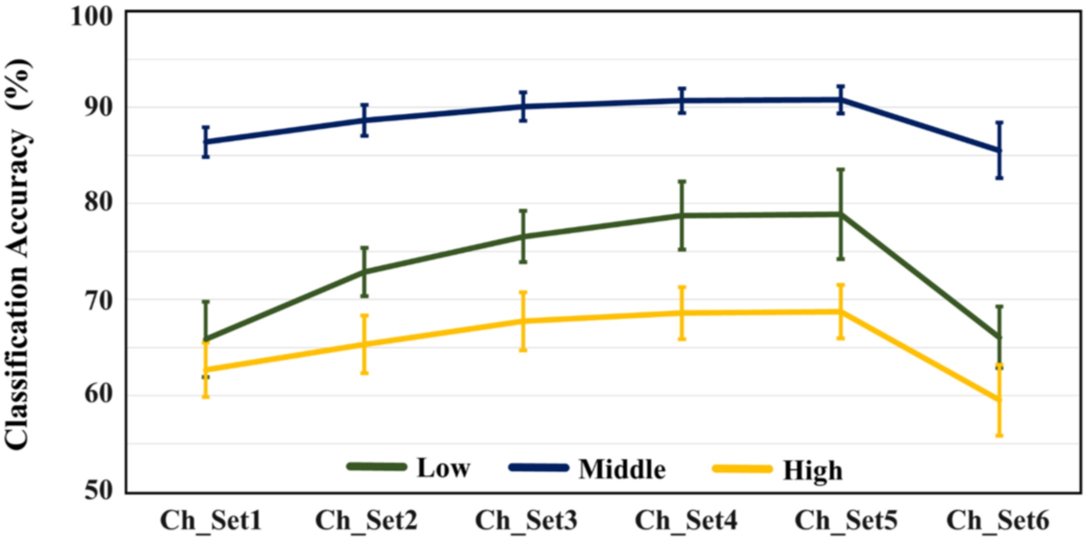

Figure 6. Changes in classification accuracy in terms of channel configuration for each stimulation frequency band. Eight channels attached to occipital areas (Ch_Set5) achieved the highest mean classification accuracy for all three frequency bands. The vertical bars indicate the standard deviations of classification accuracies for each channel set.

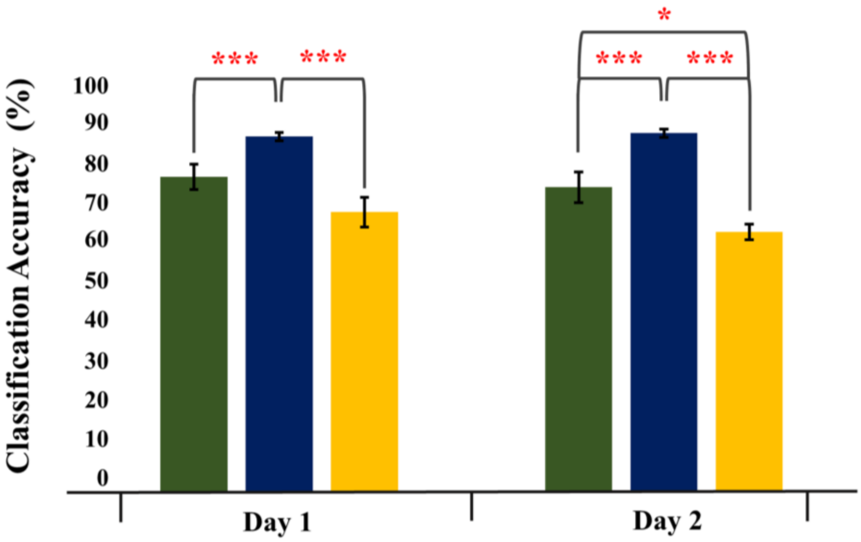

	Day 1	Day 2
Low	0.80 (± 0.03)	0.74 (± 0.04)
Middle	0.90 (± 0.01)	0.91 (± 0.01)
High	0.71 (± 0.04)	0.66 (± 0.02)

Figure 7. Mean classification accuracies for the three frequency bands for each day (RM-ANOVA: $F(2, 29) = 19.87$, $p < 0.01$; paired t-test Bonferroni corrected $p < 0.05$: middle > low = high for the first day; RM-ANOVA: $F(2, 29) = 23.09$, $p < 0.01$; paired t-test Bonferroni corrected $p < 0.05$: middle > low > high for the second day). The vertical bars indicate the standard deviations of classification accuracies for each frequency band.

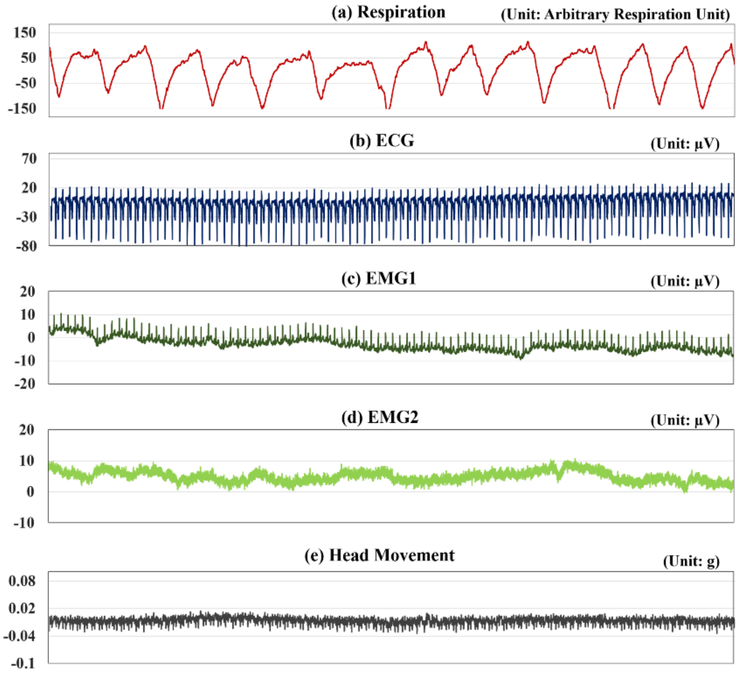

Figure 8. Examples of six types of physiological measurement data, with vendor-specific units: (a) respiration [ARU (arbitrary respiration unit)], (b) ECG [µV], (c) EMG1 (left, posterior side of the neck) [µV], (d) EMG2 (right posterior of the neck) [µV], and (e) head movement [g ≈ 9.81 m/s2].

Click here to access/download
Supplementary Material
questionnaires_answers.xlsx

Click here to access/download
Supplementary Material
Respiration_Day1.xlsx

Click here to access/download
Supplementary Material
Respiration_Day2.xlsx

Click here to access/download
Supplementary Material
ECG_Day1.xlsx

Click here to access/download
Supplementary Material
ECG_Day2.xlsx

Click here to access/download
Supplementary Material
EMG1_Day1.xlsx

Click here to access/download
Supplementary Material
EMG1_Day2.xlsx

Click here to access/download
Supplementary Material
EMG2_Day1.xlsx

Click here to access/download
Supplementary Material
EMG2_Day2.xlsx

Click here to access/download
Supplementary Material
HeadMovement_Day1.xlsx

Click here to access/download
Supplementary Material
HeadMovement_Day2.xlsx

Click here to access/download
Supplementary Material
SNR_Low(1)_5Hz.xlsx

Click here to access/download
Supplementary Material
SNR_Low(2)_5Hz.xlsx

Click here to access/download
Supplementary Material
SNR_Low(1)_5.5Hz.xlsx

Click here to access/download
Supplementary Material
SNR_Low(2)_5.5Hz.xlsx

Click here to access/download
Supplementary Material
SNR_Low(1)_6Hz.xlsx

Click here to access/download
Supplementary Material
SNR_Low(2)_6Hz.xlsx

Click here to access/download

Supplementary Material

SNR_Low(1)_6.5Hz.xlsx

Click here to access/download
Supplementary Material
SNR_Low(2)_6.5Hz.xlsx

Click here to access/download
Supplementary Material
SNR_Middle(1)_21Hz.xlsx

Click here to access/download
Supplementary Material
SNR_Middle(2)_21Hz.xlsx

Click here to access/download
Supplementary Material
SNR_Middle(1)_21.5Hz.xlsx

Click here to access/download
Supplementary Material
SNR_Middle(2)_21.5Hz.xlsx

Click here to access/download
Supplementary Material
SNR_Middle(1)_22Hz.xlsx

Click here to access/download
Supplementary Material
SNR_Middle(2)_22Hz.xlsx

Click here to access/download
Supplementary Material
SNR_Middle(1)_22.5Hz.xlsx

Click here to access/download
Supplementary Material
SNR_Middle(2)_22.5Hz.xlsx

Click here to access/download
Supplementary Material
SNR_High(1)_40Hz.xlsx

Click here to access/download
Supplementary Material
SNR_High(2)_40Hz.xlsx

Click here to access/download
Supplementary Material
SNR_High(1)_40.5Hz.xlsx

Click here to access/download
Supplementary Material
SNR_High(2)_40.5Hz.xlsx

Click here to access/download
Supplementary Material
SNR_High(1)_41Hz.xlsx

Click here to access/download
Supplementary Material
SNR_High(2)_41Hz.xlsx

Click here to access/download
Supplementary Material
SNR_High(1)_41.5Hz.xlsx

Click here to access/download
Supplementary Material
SNR_High(2)_41.5Hz.xlsx

Click here to access/download
Supplementary Material
SNR_CrossCorrelation.xlsx

Click here to access/download
Supplementary Material
Supplementary Figures.docx

Personal Cover Click here to access/download;Personal Cover;responses to the reviewers.docx

Responses to Reviewers' Comments

Ref#: GIGA-D-19-00116R1

Title: A multi-day and multi-band dataset for steady-state visual evoked potential-based brain-computer interface

Authors: Ga-Young Choi, Chang-Hee Han, Young-Jin Jung, and Han-Jeong Hwang

Dear Reviewers,

We are very grateful to the reviewers for the constructive comments. We have substantially revised our revised manuscript according to the comments and suggestions the reviewer made. Below, we summarize how we have addressed the review comments. The review comments are printed in normal font and responses in blue-bold font. The revised expressions and paragraphs are emphasized with fluorescent color in the revised manuscript.

Reviewer #2:

Comments to the Author

First of all, thank you for the revision which has clarified most comments raised, however, there are still few things to take care of.

: Thank you for the favorable comment, and giving us an opportunity to enhance the quality of our manuscript. We tried to do our best to address all the issues raised by the reviewer during the second revision period.

1. For the electrode location issue, it is better to show individual topographic maps of SNR for two different days, or to calculate similarity score (inner product or correlation) between two topographic maps of SNR. Then, authors can prove there are minor changes in electrode locations.

: Thank you for your constructive comment. As advised, to prove minor changes in electrode locations between two different days, we calculated cross-correlation coefficients for the four stimulation frequencies in each frequency band. To this end, the mean SSVEP-SNR values of each channel were estimated independently for each

stimulation frequency and each day, and then cross-correlation coefficients between the mean SSVEP-SNR values of two days were estimated for each stimulation frequency. As a result, 92.5 % of comparison cases showed cross-correlation coefficients higher than 0.9, and other cases mostly showed more than 0.8 (the minimum coefficient was 0.69), demonstrating no significant electrode location change between two different days in genenal. We provide the detailed cross-correlation results as a supplementary file (SNR_CrossCorrelation.xlsx).

We also revised the original Figure 4 showing SSVEP SNR topographic maps averaged over two days for all subjects in such a way that grand-average SSVEP SNR topographic maps are presented for each day. Note that Cross-correlation coefficients are higher than 0.99 for all comparison cases shown in Figure 4.

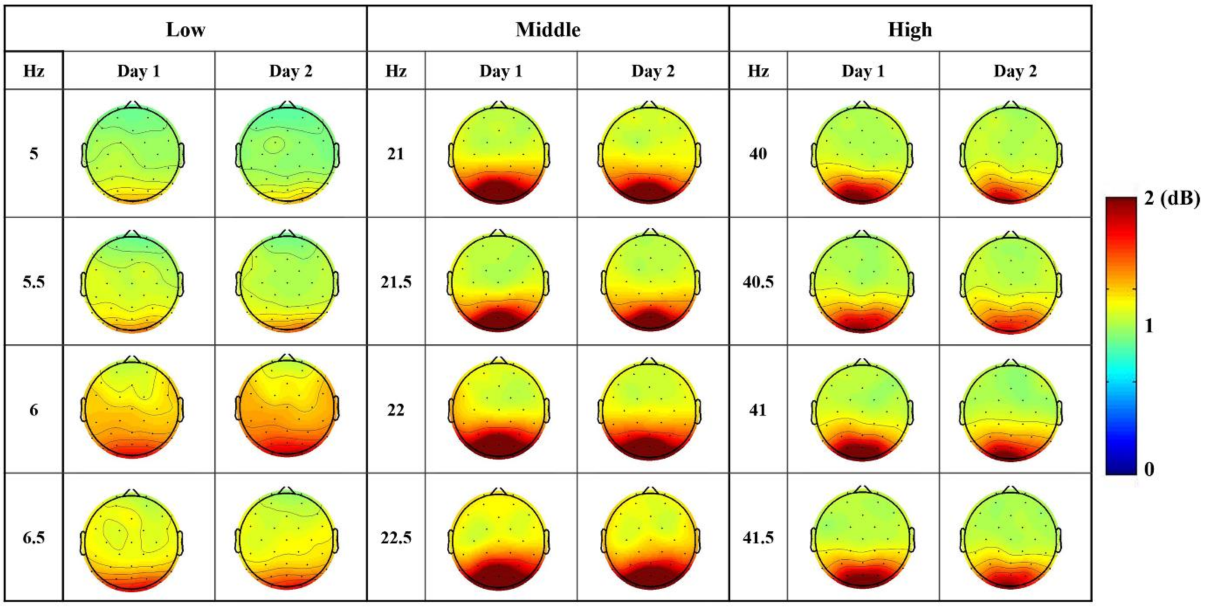

Figure 4. SSVEP SNR topographic maps averaged for each day with all subjects for the four stimulation frequencies of the low, middle, and high frequency band, respectively. The cross-correlation coefficients are higher than 0.99 for all cases.

The following sentences are also added for the new results in the result section:

i) Additionally, the Day-1 and Day-2 SSVEP topographic maps appear to be very similar, corresponding to a high cross-correlation (r > 0.99) for all comparison cases. This result demonstrates a small discrepancy between the electrode locations on the first and second days.

ii) The cross-correlation analysis results for each subject are also provided for the four stimulation frequencies in each frequency band with a supplementary file

(SNR_CrossCorrelation.xlsx).

2. Please, clarify the formula of SNR for Figure 4 in the manuscript.

: According to the reviewer's recommendation, we added the SNR formula as follows:

$$\mathrm{SNR} = \frac{n \times y(f)}{\sum_{k=1}^{n/2}[y(f+0.5\times k)+y(f-0.5\times k)} \qquad (1)$$

where n is the number of adjacent points (six in this study), y is the spectral amplitude, and f is the stimulation frequency.

3. The caption in the Figure 8(f) has a mistake. It should be 'ATU (arbitrary temperature unit)', not ARU (arbitrary respiration unit)'.

: Thank you for the kind comment, but note that we excluded all the information related to temperature based on the reviewer's advice mentioned in the next comment.

4. It is still difficult to accept the temperature data. Without Celsius/Fahrenheit unit, author cannot say that the temperature data is scientifically reasonable. I do not agree that relative changes of ATU are still useful. Readers do not know how the acquisition company scaled or calculated the temperature values. For example, subject number 2 and 23 have relatively bigger ATU than other subjects in supp. Figure 6. Without Celsius/Fahrenheit unit, authors cannot say that these data is in normal range, even other subjects. Readers who interested in temperature will keep ask about the temperature data how to convert the ATU into Celsius/Fahrenheit. Thus, it is better to remove the temperature data from the manuscript or to convert the ATU into Celsius/Fahrenheit unit.

: Thank you for the useful comment. We decided to exclude the temperature data to prevent potential confusion according to the reviewer's comment. Thus, we removed an example result of body temperature shown in Figure 8, and the Supplementary Figure 6 showing the mean body temperatures of each trial and its corresponding supplementary files (Temperature_Day1.xlsx and Temperature_Day2.xlsx).

5. The English in the manuscript should be carefully improved by native speaker, especially

for captions of the figures (including supplementary materials) in the manuscripts.

: According to the reviewer’s recommendation, we have undergone English proofreading by a native speaker for the revised manuscript including supplementary materials.
