# Supplementary material for: A multi-day and multi-band dataset for a steady-state visual-evoked potential–based brain-computer interface
Source: Gigascience. 2019 Nov 25;8(11):giz133. doi: 10.1093/gigascience/giz133 (PMC6876666; doi:10.1093/gigascience/giz133)
Supplement: giz133_Supplemental_Files [file giz133_supplemental_files.zip › Supplementary Figures.docx]

**A multi-day and multi-band dataset for steady-state visual evoked potential based Brain-Computer Interface**

Ga-Young Choi^1^, Chang-Hee Han^2^, Young-Jin Jung^3^, Han-Jeong Hwang^1,*^

**E-mail:** [cgy326@naver.com](mailto:cgy326@naver.com), [zeros8706@naver.com](mailto:zeros8706@naver.com), [microbme@outlook.com](mailto:microbme@outlook.com), h2j@ kumoh.ac.kr

^1^Department of Medical IT Convergence Engineering, Kumoh National Institute of Technology, Gumi 39177, Republic of Korea

^2^Machine Learning Group, Berlin Institute of Technology (TU Berlin), 10623 Berlin, Germany

^3^Department of Radiological Science, Dongseo University, Busan 47011, Republic of Korea

**Number of Figures: 5**

**Corresponding Author Information:**

**Name:** Han-Jeong Hwang

**Address:** Kumoh National Institute of Technology, 350-27, Gumi-si, Gyeongsangbuk-do, Republic of Korea

**Tel.:** +82-054-478-7783

**E-mail:** h2j@kumoh.ac.kr


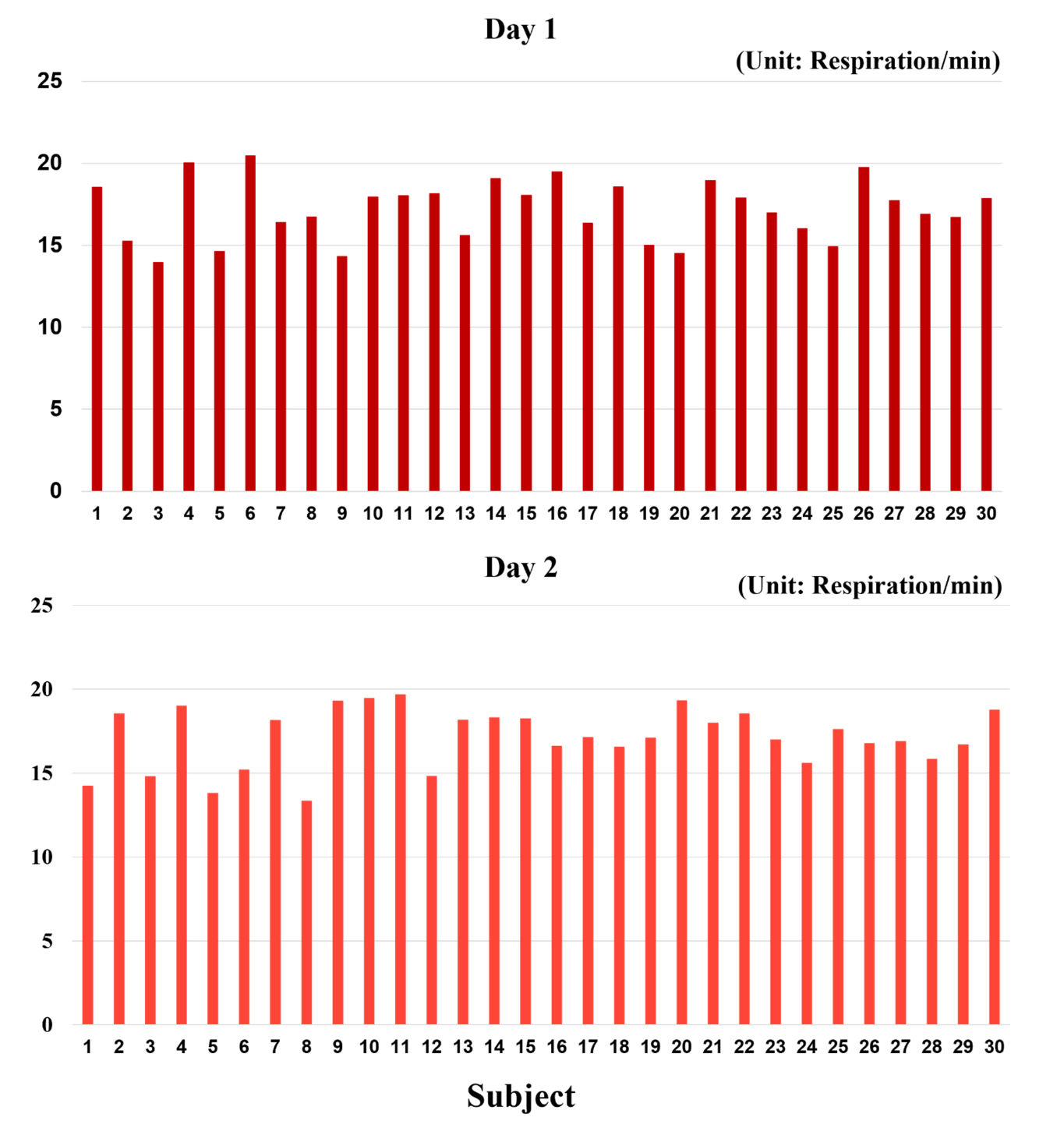


**Supplementary Figure 1**. Respiratory rates of each subject recorded on two different days. For detailed information, refer to the corresponding supplementary files (Respiration_Day1.xlxs and Respiration_Day2.xlsx).


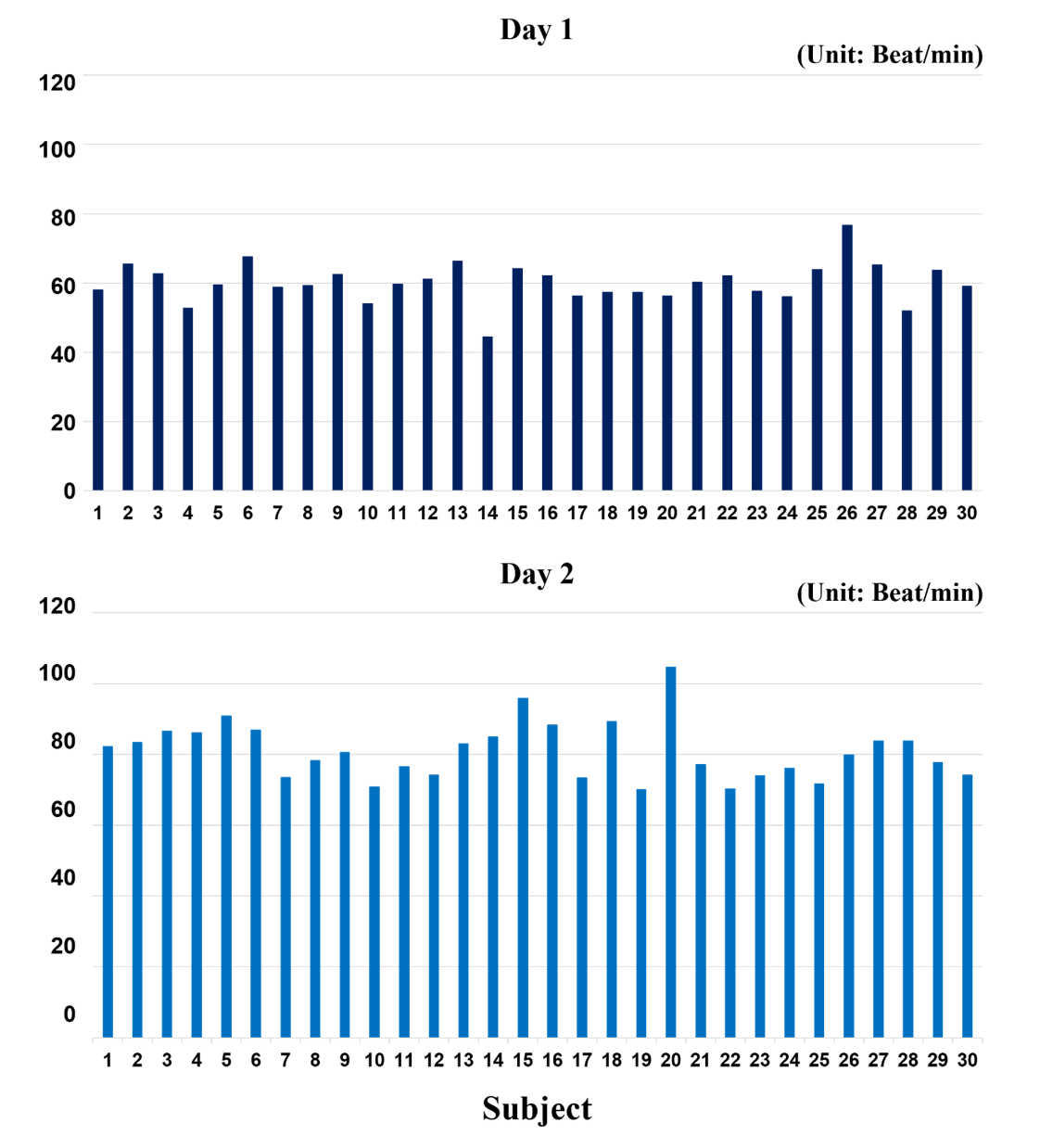


**Supplementary Figure 2.** Heart rates of each subject recorded on two different days. For detailed information, refer to the corresponding supplementary files (ECG_Day1.xlxs and ECG_Day2.xlsx).


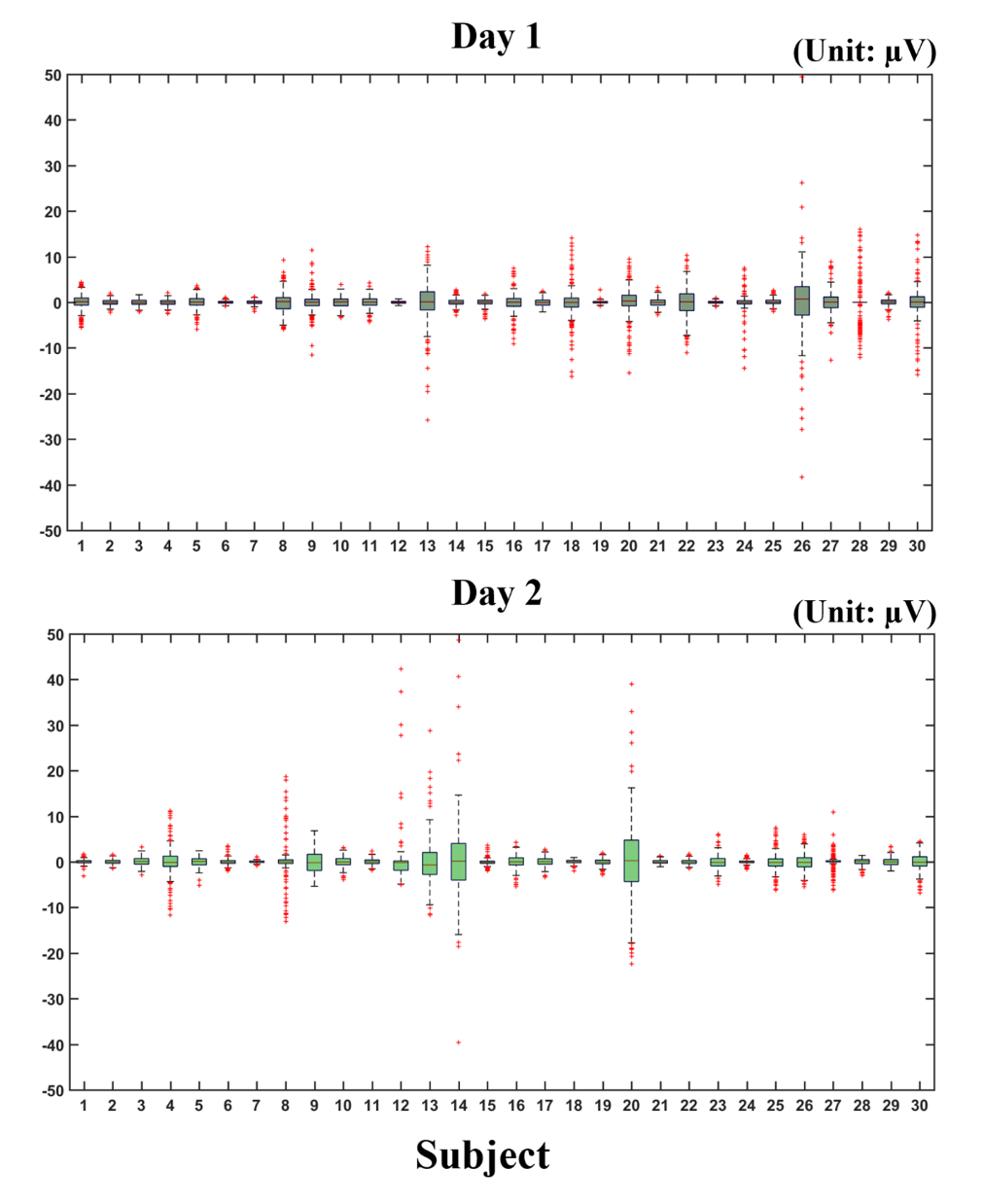


**Supplementary Figure 3.** Mean trial EMG values for each subject estimated using the EMG1 channel data. For detailed information, refer to the corresponding supplementary files (EMG1_Day1.xlxs and EMG1_Day2.xlsx).


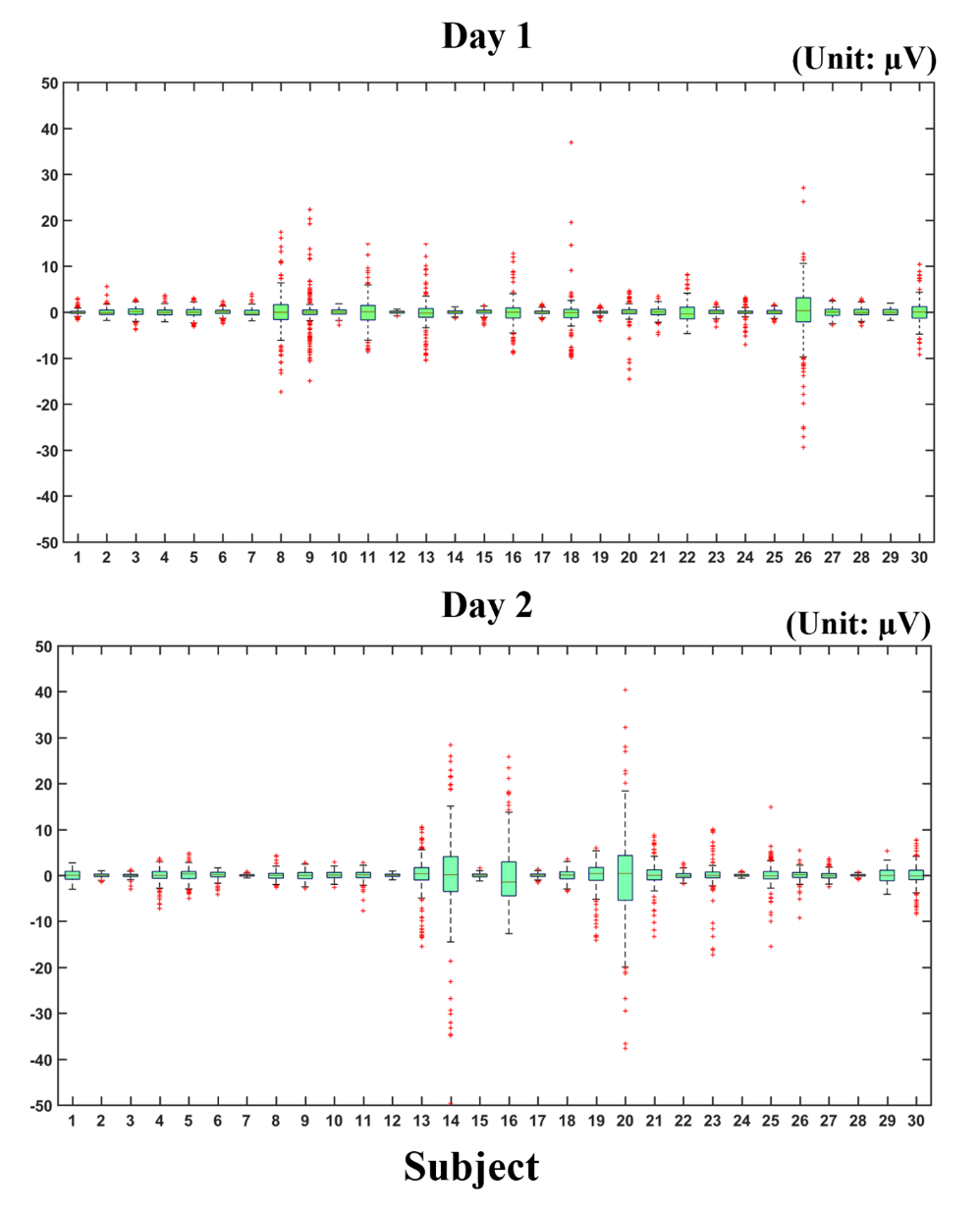


**Supplementary Figure 4.** Mean trial EMG values for each subject estimated using the EMG2 channel data. For detailed information, refer to the corresponding supplementary files (EMG2_Day1.xlxs and EMG2_Day2.xlsx).


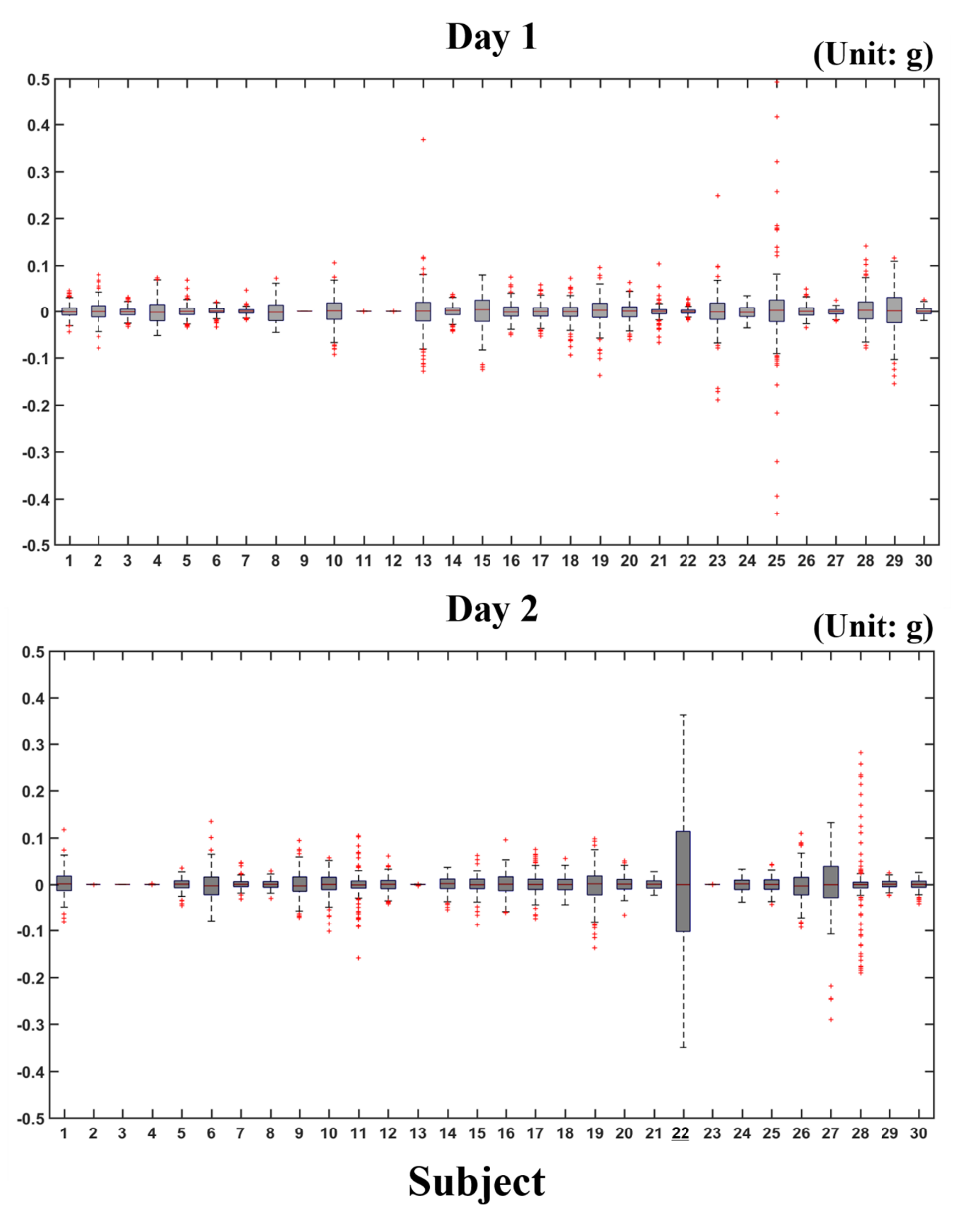


**Supplementary Figure 5.** Mean trial IMU values for each subject, and each day. For detailed information, refer to the corresponding supplementary files (HeadMovement_Day1.xlxs and HeadMovement_Day2.xlsx).
