# Supplementary material for: A multi-day and multi-band dataset for a steady-state visual-evoked potential–based brain-computer interface
Source: Gigascience. 2019 Nov 25;8(11):giz133. doi: 10.1093/gigascience/giz133 (PMC6876666; doi:10.1093/gigascience/giz133)
Supplement: giz133_Response_to_Reviewer_Comments_Original_Submission [file giz133_response_to_reviewer_comments_original_submission.pdf]

Author's Response To Reviewer Comments

Close

We responded to all comments the reviewers made in the Personal Cover file.

Close
