# Supplementary material for: A multi-day and multi-band dataset for a steady-state visual-evoked potential–based brain-computer interface
Source: Gigascience. 2019 Nov 25;8(11):giz133. doi: 10.1093/gigascience/giz133 (PMC6876666; doi:10.1093/gigascience/giz133)
Supplement: giz133_Reviewer_1_Report_Original_Submission — Dan Zhang -- 5/15/2019 Reviewed [file giz133_reviewer_1_report_original_submission.pdf]

Reviewer Report

Title: A multi-day and multi-band dataset for a steady-state visual-evoked potential-based brain-computer interface

Version: Original Submission **Date:** 5/15/2019

Reviewer name: Dan Zhang

Reviewer Comments to Author:

The manuscript reports an EEG-based SSVEP BCI dataset. Compared to existing datasets, the present dataset has its unique features of multi-band (covering typical SSVEP frequency bands), multi-session, and multi-day recordings. The number of participants is reasonable. Data analysis supports the validity of the dataset. Due to the limited number of publicly available SSVEP BCI datasets as compared to the popularity of the SSVEP BCI applications, the reported dataset is timely and could have important contribution to the BCI research community. Therefore, I suggest acceptance of the manuscript, with the following minor comments.

1. While I agree thirty subjects is reasonable for a dataset, it would be clearer to have some explanations on this. And, whether they had previous (SSVEP) BCI experiences should be reported.
2. Please report the duty cycle of the steady-state stimulations: I assume 50% on and 50% off?
3. The selection of the frequency bands need further explanation. Especially, why not the alpha band included?
4. The rationale for the inclusion of the bio-signals (ECG, EMG etc.) should be given prior to the Results session, preferably with references to previous related studies.
5. Since there were two recording days, what measures have the authors taken to control possible position differences of the EEG recording sites across days?
6. Figure 3: it is misleading to state 'SSVEP topographic maps', as the authors already mentioned in the main text, the frontal activation could be eye movement related activities that happened to be at the steady-state stimulation frequencies. It could be better to state 'Topographic maps at the SSVEP frequencies'.

Level of Interest

Please indicate how interesting you found the manuscript: Choose an item.

Quality of Written English

Please indicate the quality of language in the manuscript: Choose an item.

Declaration of Competing Interests

Please complete a declaration of competing interests, considering the following questions:

- Have you in the past five years received reimbursements, fees, funding, or salary from an organisation that may in any way gain or lose financially from the publication of this manuscript, either now or in the future?
- Do you hold any stocks or shares in an organisation that may in any way gain or lose financially from the publication of this manuscript, either now or in the future?
- Do you hold or are you currently applying for any patents relating to the content of the manuscript?
- Have you received reimbursements, fees, funding, or salary from an organization that holds or has applied for patents relating to the content of the manuscript?
- Do you have any other financial competing interests?
- Do you have any non-financial competing interests in relation to this paper?

If you can answer no to all of the above, write 'I declare that I have no competing interests' below. If your reply is yes to any, please give details below.

I declare that I have no competing interests.

I agree to the open peer review policy of the journal. I understand that my name will be included on my report to the authors and, if the manuscript is accepted for publication, my named report including any attachments I upload will be posted on the website along with the authors' responses. I agree for my report to be made available under an Open Access Creative Commons CC-BY license (http://creativecommons.org/licenses/by/4.0/). I understand that any comments which I do not wish to be included in my named report can be included as confidential comments to the editors, which will not be published.

Choose an item.

To further support our reviewers, we have joined with Publons, where you can gain additional credit to further highlight your hard work (see: https://publons.com/journal/530/gigascience). On publication of this paper, your review will be automatically added to Publons, you can then choose whether or not to claim your Publons credit. I understand this statement.

Yes Choose an item.
