# Supplementary material for: A multi-day and multi-band dataset for a steady-state visual-evoked potential–based brain-computer interface
Source: Gigascience. 2019 Nov 25;8(11):giz133. doi: 10.1093/gigascience/giz133 (PMC6876666; doi:10.1093/gigascience/giz133)
Supplement: giz133_Reviewer_2_Report_Original_Submission — Hohyun Cho -- 5/28/2019 Reviewed [file giz133_reviewer_2_report_original_submission.pdf]

Reviewer Report

Title: A multi-day and multi-band dataset for a steady-state visual-evoked potential-based brain-computer interface

Version: Original Submission **Date:** 5/28/2019

Reviewer name: Hohyun Cho

Reviewer Comments to Author:

This data note is about steady-state visual evoked potential (SSVEP) dataset for brain-computer interface (BCI) research field. The dataset includes multiple days recording with multiple frequencies for visual stimulation and physiological data including respiration, electrocardiography, electromyography, head movement, and body temperature. This dataset well aimed challenges in BCI research, such as session variability and subject variability. The dataset is reasonable to be published in GigaScience, however, the manuscript needs major revision to be published as following comments;
1. There is no validation results for number of bad trials/channels/subjects/day-sessions in the datasets. Even if there are no bad trials/channels/subjects/day-sessions, author should prove the dataset is clean, for an example, by using signal-to-noise ratio (SNR) between background noise and SSVEP in EEG.
2. There is no proof/validation for physiological data is reasonable, for example, checking number of in-/ex-hale, heart beating rates, noise level of EMG, and body temperature in normal range.
There is no information in the y-axis of Figure 7 about the unit for each physiological measurement, for example, unit of head movement data and body temperature.
Minor comments are as below;
1. For necessary controls, recording date and time for each day-session data should be specified. Further, the EEG cap positions on multiple days cannot be completely same for each subject. Author should mention the reasonable method to minimize the problem in this manuscript.
2. There are some typos and wrong word in the manuscript.
* Findings paragraph in Abstract - 'reasonalbe'
* Page #4 line #22 - 'choie'
* Page #11 line #6 and A-7 in Table 2 - 'Drug'
In general, 'drug' means illegal medicine, such as marijuana, so, 'medicine' would be correct one.
3. There is no detailed explanation for the unit of color map in Figure 3.
4. Font sizes are different in Figure 3 (c).

Level of Interest

Please indicate how interesting you found the manuscript: Choose an item.

Quality of Written English

Please indicate the quality of language in the manuscript: Choose an item.

Declaration of Competing Interests

Please complete a declaration of competing interests, considering the following questions:

- Have you in the past five years received reimbursements, fees, funding, or salary from an organisation that may in any way gain or lose financially from the publication of this manuscript, either now or in the future?
- Do you hold any stocks or shares in an organisation that may in any way gain or lose financially from the publication of this manuscript, either now or in the future?
- Do you hold or are you currently applying for any patents relating to the content of the manuscript?
- Have you received reimbursements, fees, funding, or salary from an organization that holds or has applied for patents relating to the content of the manuscript?
- Do you have any other financial competing interests?
- Do you have any non-financial competing interests in relation to this paper?

If you can answer no to all of the above, write 'I declare that I have no competing interests' below. If your reply is yes to any, please give details below.

I declare that I have no competing interests.

I agree to the open peer review policy of the journal. I understand that my name will be included on my report to the authors and, if the manuscript is accepted for publication, my named report including any attachments I upload will be posted on the website along with the authors' responses. I agree for my report to be made available under an Open Access Creative Commons CC-BY license (http://creativecommons.org/licenses/by/4.0/). I understand that any comments which I do not wish to be included in my named report can be included as confidential comments to the editors, which will not be published.

Choose an item.

To further support our reviewers, we have joined with Publons, where you can gain additional credit to further highlight your hard work (see: https://publons.com/journal/530/gigascience). On publication of this paper, your review will be automatically added to Publons, you can then choose whether or not to claim your Publons credit. I understand this statement.

Yes Choose an item.
