# Supplementary material for: A multi-day and multi-band dataset for a steady-state visual-evoked potential–based brain-computer interface
Source: Gigascience. 2019 Nov 25;8(11):giz133. doi: 10.1093/gigascience/giz133 (PMC6876666; doi:10.1093/gigascience/giz133)
Supplement: giz133_Reviewer_2_Report_Revision_1 — Hohyun Cho -- 9/11/2019 Reviewed [file giz133_reviewer_2_report_revision_1.pdf]

Reviewer Report

Title: A multi-day and multi-band dataset for a steady-state visual-evoked potential-based brain-computer interface

Version: Revision 1 **Date:** 9/11/2019

Reviewer name: Hohyun Cho

Reviewer Comments to Author:

First of all, thank you for the revision which has clarified most comments raised, however, there are still few things to take care of.
1. For the electrode location issue, it is better to show individual topographic maps of SNR for two different days, or to calculate similarity score (inner product or correlation) between two topographic maps of SNR. Then, authors can prove there are minor changes in electrode locations.
2. Please, clarify the formula of SNR for Figure 4 in the manuscript.
3. The caption in the Figure 8(f) has a mistake. It should be 'ATU (arbitrary temperature unit)', not 'ARU (arbitrary respiration unit).'
4. It is still difficult to accept the temperature data. Without celsius/fahrenheit unit, author cannot say that the temperature data is scientifically reasonable.
I do not agree that relative changes of ATU are still useful.
Readers do not know how the acquisition company scaled or calculated the temperature values.
For example, subject number 2 and 23 have relatively bigger ATU than other subjects in supp. figure 6.
Without celsius/fahrenheit unit, authors cannot say that theses data is in normal range, even other subjects.
Readers who interested in temperature will keep ask about the temperature data how to convert the ATU into celsius/fahrenheit.
Thus, it is better to remove the temperature data from the manuscript or to convert the ATU into celsius/fahrenheit unit.
5. The English in the manuscript should be carefully improved by native speaker, especially for captions of the figures (including supplementary materials) in the manuscript.

Level of Interest

Please indicate how interesting you found the manuscript: Choose an item.

Quality of Written English

Please indicate the quality of language in the manuscript: Choose an item.

Declaration of Competing Interests

Please complete a declaration of competing interests, considering the following questions:

- Have you in the past five years received reimbursements, fees, funding, or salary from an organisation that may in any way gain or lose financially from the publication of this manuscript, either now or in the future?
- Do you hold any stocks or shares in an organisation that may in any way gain or lose financially from the publication of this manuscript, either now or in the future?
- Do you hold or are you currently applying for any patents relating to the content of the manuscript?
- Have you received reimbursements, fees, funding, or salary from an organization that holds or has applied for patents relating to the content of the manuscript?
- Do you have any other financial competing interests?
- Do you have any non-financial competing interests in relation to this paper?

If you can answer no to all of the above, write 'I declare that I have no competing interests' below. If your reply is yes to any, please give details below.

I declare that I have no competing interests.

I agree to the open peer review policy of the journal. I understand that my name will be included on my report to the authors and, if the manuscript is accepted for publication, my named report including any attachments I upload will be posted on the website along with the authors' responses. I agree for my report to be made available under an Open Access Creative Commons CC-BY license (http://creativecommons.org/licenses/by/4.0/). I understand that any comments which I do not wish to be included in my named report can be included as confidential comments to the editors, which will not be published.

Choose an item.

To further support our reviewers, we have joined with Publons, where you can gain additional credit to further highlight your hard work (see: https://publons.com/journal/530/gigascience). On publication of this paper, your review will be automatically added to Publons, you can then choose whether or not to claim your Publons credit. I understand this statement.

Yes Choose an item.
