# Supplementary material for: A multi-day and multi-band dataset for a steady-state visual-evoked potential–based brain-computer interface
Source: Gigascience. 2019 Nov 25;8(11):giz133. doi: 10.1093/gigascience/giz133 (PMC6876666; doi:10.1093/gigascience/giz133)
Supplement: giz133_Supplemental_Files [file giz133_supplemental_files.zip › responses to the reviewers.docx]

**Responses to Reviewers’ Comments**

Ref#: GIGA-D-19-00116R1

Title: A multi-day and multi-band dataset for steady-state visual evoked potential-based brain-computer interface

Authors: Ga-Young Choi, Chang-Hee Han, Young-Jin Jung, and Han-Jeong Hwang

**Dear Reviewers,**

**We are very grateful to the reviewers for the constructive comments. We have substantially revised our revised manuscript according to the comments and suggestions the reviewer made. Below, we summarize how we have addressed the review comments. The review comments are printed in normal font and responses in blue-bold font. The revised expressions and paragraphs are emphasized with fluorescent color in the revised manuscript.**

**Reviewer #2:**

**Comments to the Author**

First of all, thank you for the revision which has clarified most comments raised, however, there are still few things to take care of.

**: Thank you for the favorable comment, and giving us an opportunity to enhance the quality of our manuscript. We tried to do our best to address all the issues raised by the reviewer during the second revision period.**

1. For the electrode location issue, it is better to show individual topographic maps of SNR for two different days, or to calculate similarity score (inner product or correlation) between two topographic maps of SNR. Then, authors can prove there are minor changes in electrode locations.

**: Thank you for your constructive comment. As advised, to prove minor changes in electrode locations between two different days, we calculated cross-correlation coefficients for the four stimulation frequencies in each frequency band. To this end, the mean SSVEP-SNR values of each channel were estimated independently for each stimulation frequency and each day, and then cross-correlation coefficients between the mean SSVEP-SNR values of two days were estimated for each stimulation frequency. As a result, 92.5 % of comparison cases showed cross-correlation coefficients higher than 0.9, and other cases mostly showed more than 0.8 (the minimum coefficient was 0.69), demonstrating no significant electrode location change between two different days in genenal. We provide the detailed cross-correlation results as a supplementary file (SNR_CrossCorrelation.xlsx).**

**We also revised the original Figure 4 showing SSVEP SNR topographic maps averaged over two days for all subjects in such a way that grand-average SSVEP SNR topographic maps are presented for each day. Note that Cross-correlation coefficients are higher than 0.99 for all comparison cases shown in Figure 4.**

Figure 4. SSVEP SNR topographic maps averaged for each day with all subjects for the four stimulation frequencies of the low, middle, and high frequency band, respectively. The cross-correlation coefficients are higher than 0.99 for all cases**.**

**The following sentences are also added for the new results in the result section:**

i) Additionally, the Day-1 and Day-2 SSVEP topographic maps appear to be very similar, corresponding to a high cross-correlation (r > 0.99) for all comparison cases. This result demonstrates a small discrepancy between the electrode locations on the first and second days.

ii) The cross-correlation analysis results for each subject are also provided for the four stimulation frequencies in each frequency band with a supplementary file (SNR_CrossCorrelation.xlsx).

2. Please, clarify the formula of SNR for Figure 4 in the manuscript.

**: According to the reviewer’s recommendation, we added the SNR formula as follows:**

$SNR= \frac{n \times y(f)}{\sum_{k=1}^{n/2} [y\left( f+0.5 \times k \right)+y(f-0.5\times k)}$	(1)

where *n* is the number of adjacent points (six in this study), *y* is the spectral amplitude, and *f* is the stimulation frequency.

3. The caption in the Figure 8(f) has a mistake. It should be ‘ATU (arbitrary temperature unit)’, not ARU (arbitrary respiration unit)’.

**: Thank you for the kind comment, but note that we excluded all the information related to temperature based on the reviewer’s advice mentioned in the next comment.**

4. It is still difficult to accept the temperature data. Without Celsius/Fahrenheit unit, author cannot say that the temperature data is scientifically reasonable. I do not agree that relative changes of ATU are still useful. Readers do not know how the acquisition company scaled or calculated the temperature values. For example, subject number 2 and 23 have relatively bigger ATU than other subjects in supp. Figure 6. Without Celsius/Fahrenheit unit, authors cannot say that these data is in normal range, even other subjects. Readers who interested in temperature will keep ask about the temperature data how to convert the ATU into Celsius/Fahrenheit. Thus, it is better to remove the temperature data from the manuscript or to convert the ATU into Celsius/Fahrenheit unit.

**: Thank you for the useful comment. We decided to exclude the temperature data to prevent potential confusion according to the reviewer’s comment. Thus, we removed an example result of body temperature shown in Figure 8, and the Supplementary Figure 6 showing the mean body temperatures of each trial and its corresponding supplementary files (Temperature_Day1.xlsx and Temperature_Day2.xlsx).**

5. The English in the manuscript should be carefully improved by native speaker, especially for captions of the figures (including supplementary materials) in the manuscripts.

**: According to the reviewer’s recommendation, we have undergone English proofreading by a native speaker for the revised manuscript including supplementary materials.**
